# KCND3 potassium channel gene variant confers susceptibility to electrocardiographic early repolarization pattern

**DOI:** 10.1172/jci.insight.131156

**Published:** 2019-12-05

**Authors:** Alexander Teumer, Teresa Trenkwalder, Thorsten Kessler, Yalda Jamshidi, Marten E. van den Berg, Bernhard Kaess, Christopher P. Nelson, Rachel Bastiaenen, Marzia De Bortoli, Alessandra Rossini, Isabel Deisenhofer, Klaus Stark, Solmaz Assa, Peter S. Braund, Claudia Cabrera, Anna F. Dominiczak, Martin Gögele, Leanne M. Hall, M. Arfan Ikram, Maryam Kavousi, Karl J. Lackner, Lifelines Cohort Study, Christian Müller, Thomas Münzel, Matthias Nauck, Sandosh Padmanabhan, Norbert Pfeiffer, Tim D. Spector, Andre G. Uitterlinden, Niek Verweij, Uwe Völker, Helen R. Warren, Mobeen Zafar, Stephan B. Felix, Jan A. Kors, Harold Snieder, Patricia B. Munroe, Cristian Pattaro, Christian Fuchsberger, Georg Schmidt, Ilja M. Nolte, Heribert Schunkert, Peter P. Pramstaller, Philipp S. Wild, Pim van der Harst, Bruno H. Stricker, Renate B. Schnabel, Nilesh J. Samani, Christian Hengstenberg, Marcus Dörr, Elijah R. Behr, Wibke Reinhard

**Affiliations:** 1 Institute for Community Medicine, University Medicine Greifswald, Greifswald, Germany.; 2 German Center for Cardiovascular Research (DZHK), partner site Greifswald, Greifswald, Germany.; 3 Klinik für Herz-und Kreislauferkrankungen, Deutsches Herzzentrum München, School of Medicine, Technical University of Munich, Munich, Germany.; 4 Genetics Research Centre, Institute of Molecular and Clinical Sciences, Saint George’s University of London, London, United Kingdom.; 5 Department of Epidemiology, Erasmus MC University Medical Center Rotterdam, Rotterdam, Netherlands.; 6 Medizinische Klinik I, St. Josefs-Hospital, Wiesbaden, Germany.; 7 Department of Cardiovascular Sciences, BHF Cardiovascular Research Centre, Leicester, United Kingdom.; 8 National Institute for Health Research (NIHR) Leicester Biomedical Research Centre, University of Leicester, Leicester, United Kingdom.; 9 Cardiology Clinical Academic Group, Institute of Molecular and Clinical Sciences, Saint George’s, University of London, London, United Kingdom.; 10 Eurac Research, Institute for Biomedicine, affiliated with the University of Lübeck, Bolzano, Italy.; 11 Department of Genetic Epidemiology, University Regensburg, Regensburg, Germany.; 12 Department of Cardiology, University of Groningen, University Medical Center Groningen, Groningen, Netherlands.; 13 Clinical Pharmacology, William Harvey Research Institute, and; 14 NIHR Barts Cardiovascular Biomedical Research Centre, Barts and The London School of Medicine, Queen Mary University of London, London, United Kingdom.; 15 Centre for Translational Bioinformatics, William Harvey Research Institute, Barts and the London, London, United Kingdom, and School of Medicine and Dentistry, Charterhouse Square, London, United Kingdom.; 16 Institute of Cardiovascular and Medical Sciences, College of Medical, Veterinary and Life Sciences, University of Glasgow, Glasgow, Scotland, United Kingdom.; 17 Institute of Clinical Chemistry and Laboratory Medicine, University Medical Center of the Johannes Gutenberg, University Mainz, Mainz, Germany.; 18 DZHK, partner site Rhine-Main, Mainz, Germany.; 19 The Lifelines Cohort Study detailed in the Supplemental Acknowledgments.; 20 University Heart & Vascular Center Hamburg, Hamburg, Germany.; 21 DZHK, partner site Hamburg/Kiel/Lübeck, Hamburg, Germany.; 22 Cardiology I, Center for Cardiology, University Medical Center of the Johannes Gutenberg, University Mainz, Mainz, Germany.; 23 Institute of Clinical Chemistry and Laboratory Medicine, University Medicine Greifswald, Greifswald, Germany.; 24 Department of Ophthalmology, University Medical Center of the Johannes Gutenberg, University Mainz, Mainz, Germany.; 25 Department of Twin Research and Genetic Epidemiology, King’s College London, London, United Kingdom.; 26 Interfaculty Institute for Genetics and Functional Genomics and; 27 Department of Internal Medicine B, University Medicine Greifswald, Greifswald, Germany.; 28 Department of Medical Informatics, Erasmus MC University Medical Center Rotterdam, Rotterdam, Netherlands.; 29 Department of Epidemiology, University of Groningen, University Medical Center Groningen, Groningen, Netherlands.; 30 Innere Medizin I, Klinikum rechts der Isar, Technical University Munich, Munich, Germany.; 31 DZHK, partner site Munich Heart Alliance, Munich, Germany.; 32 Preventive Cardiology and Preventive Medicine, Center for Cardiology, University Medical Center of the Johannes Gutenberg, University Mainz, Mainz, Germany.; 33 Center for Thrombosis and Hemostasis, University Medical Center of the Johannes Gutenberg, University Mainz, Mainz, Germany.; 34 Department of Cardiology, Division of Heart and Lungs, University Medical Center Utrecht, Utrecht, Netherlands.; 35 Division of Cardiology, Department of Internal Medicine II, Medical University of Vienna, Vienna, Austria.; 36 Cardiology Clinical Academic Group, Institute of Molecular and Clinical Sciences, Saint George’s University of London, London, United Kingdom.; 37 Saint George’s University Hospitals NHS Foundation Trust, London, United Kingdom.

**Keywords:** Cardiology, Genetics, Arrhythmias, Genetic variation, Ion channels

## Abstract

**BACKGROUND:**

The presence of an early repolarization pattern (ERP) on the surface ECG is associated with risk of ventricular fibrillation and sudden cardiac death. Family studies have shown that ERP is a highly heritable trait, but molecular genetic determinants are unknown.

**METHODS:**

To identify genetic susceptibility loci for ERP, we performed a GWAS and meta-analysis in 2,181 cases and 23,641 controls of European ancestry.

**RESULTS:**

We identified a genome-wide significant (*P* < 5 × 10^–8^) locus in the potassium voltage-gated channel subfamily D member 3 (*KCND3*) gene that was successfully replicated in additional 1,124 cases and 12,510 controls. A subsequent joint meta-analysis of the discovery and replication cohorts identified rs1545300 as the lead SNP at the *KCND3* locus (OR 0.82 per minor T allele, *P* = 7.7 × 10^–^12) but did not reveal additional loci. Colocalization analyses indicate causal effects of *KCND3* gene expression levels on ERP in both cardiac left ventricle and tibial artery.

**CONCLUSIONS:**

In this study, we identified for the first time to our knowledge a genome-wide significant association of a genetic variant with ERP. Our findings of a locus in the *KCND3* gene provide insights not only into the genetic determinants but also into the pathophysiological mechanism of ERP, discovering a promising candidate for functional studies.

**FUNDING:**

This project was funded by the German Center for Cardiovascular Research (DZHK Shared Expertise SE081 – STATS). For detailed funding information per study, see the Supplemental Acknowledgments.

## Abstract 2

A large scale GWAS identifies KCND3 as a gene associated with the early repolarization pattern.

## Graphical abstract

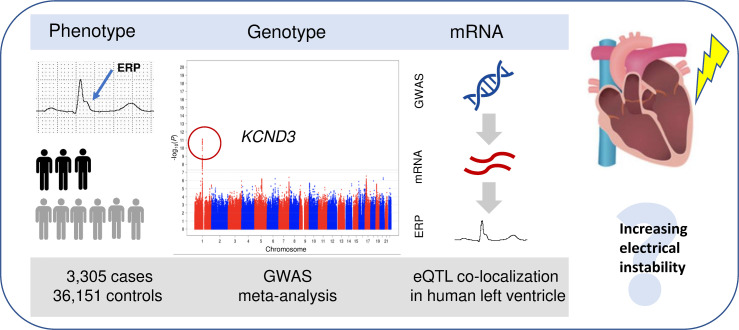

## Introduction

The early repolarization pattern (ERP) is a common ECG finding characterized by an elevation at the QRS-ST junction (J point) of at least 0.1 mV in 2 adjacent ECG leads. The prevalence of ERP in the general population ranges from 2% to 13% and is more common in young athletic men (1–5). The classical notion of ERP being a benign ECG phenotype was challenged in 2008 by a landmark study by Haïssaguerre and colleagues showing an association of ERP with increased risk of ventricular fibrillation and sudden cardiac death (6): the early repolarization syndrome (ERS) (7). Since then, several studies have demonstrated an elevated risk of cardiovascular and all-cause mortality in individuals with ERP, underscoring its arrhythmogenic potential (2, 8, 9). Although the mechanistic basis for malignant arrhythmias in ERS is unclear, it has been suggested that they occur as a result of an augmented transmural electrical dispersion of repolarization (10). Ex vivo studies point toward a central role of the cardiac transient outward potassium current (I_to_) in the development of both ERP and ERS (11). Furthermore, candidate genetic association studies have highlighted a role for several genes encoding cardiac ion channels in the development of ERP and ERS (12–15). These genes include gain-of-function variants in I_K_-ATP channels (*KCNJ8*, *ABCC9*) and loss-of-function variants in cardiac L-type calcium channels (*CACNA1C*, *CACNB2b*, *CACNA2D1*) and sodium channels (*SCN5A*, *SCN10A*) (16). Interestingly, coexistence of 2 genetic variants in different ion channel genes with opposing effects can be observed leading to phenotypic incomplete penetrance of ERP (15). However, data from functional studies confirming causality are scarce (17).

Studies among first-degree relatives of patients with sudden arrhythmic death syndrome show that ERP is more prevalent in family members than in controls, indicating that ERP is an important potentially inheritable proarrhythmic trait (18, 19). Moreover, in family studies, the heritability estimate for the presence of ERP was *h*^2^ = 0.49 (20). However, estimates for common SNP heritability from unrelated individuals are lower (21). This may explain why the only GWAS on ERP to date failed to identify genetic variants reaching genome-wide significance (22) and indicates the need for larger GWAS with more power.

In order to identify genetic variations that convey susceptibility to ERP, we performed a GWAS and meta-analysis in individuals with European ancestry, comprising 2,181 ERP cases and 23,641 controls from 8 cohorts that formed the discovery stage. The findings were taken forward to a replication stage in 1,124 cases and 12,510 controls from an 4 additional cohorts. To maximize statistical power for locus discovery, we subsequently performed a combined discovery and replication cohort GWAS meta-analysis of 3,305 ERP cases and 36,151 controls.

## Results

Clinical characteristics of the study cohorts are depicted in Table 1. The proportion of ERP based on the definition by Haïssaguerre and Macfarlane (6, 23) ranged from 6% to 14%, which is in line with the previously reported prevalence in the general population (2–4).

### Variants associated with ERP.

**Figure 1:**
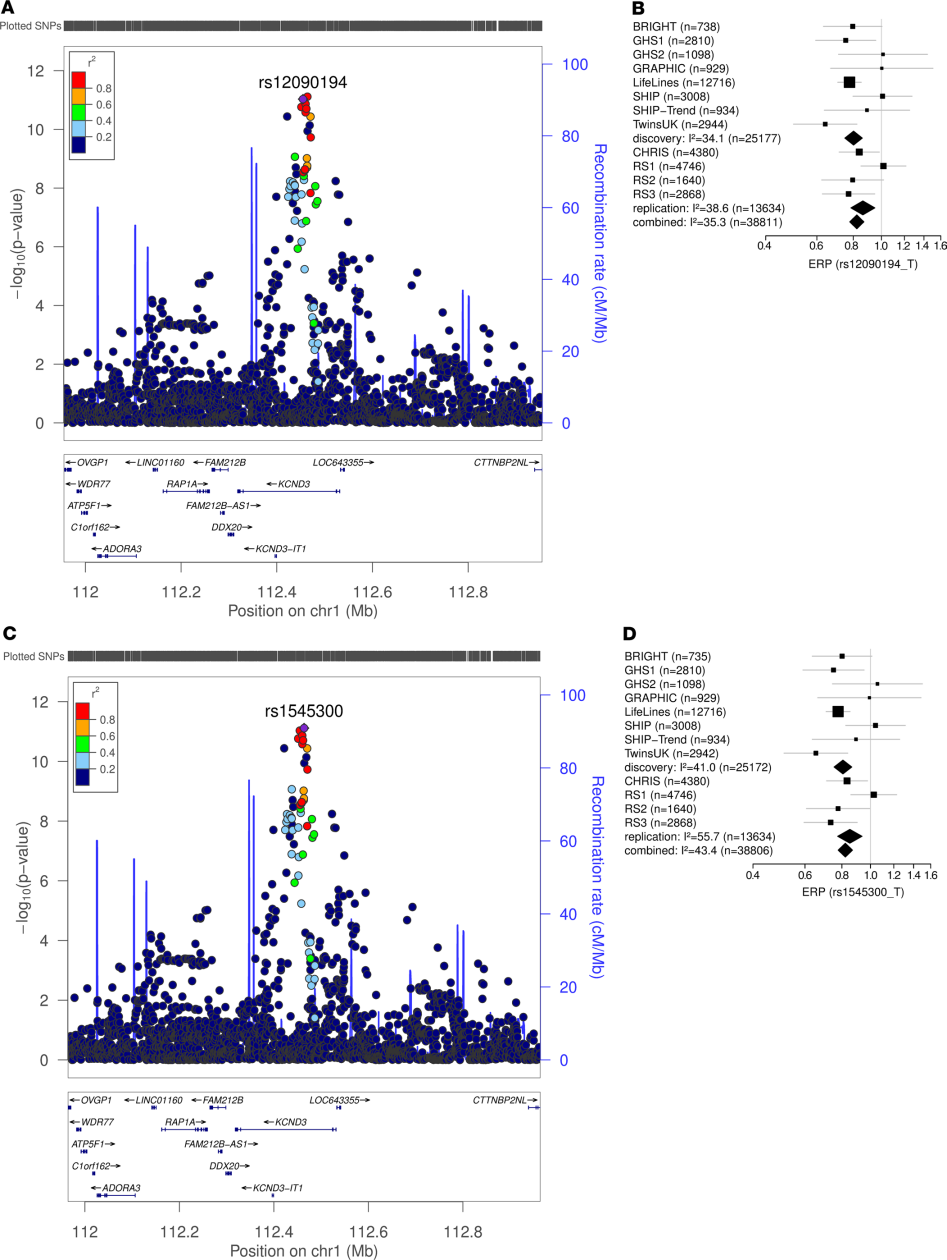
GWAS results for the *KCND3* locus. The results of the combined early repolarization pattern (ERP) GWAS results for the *KCND3* locus are shown for the replicated discovery-stage lead SNP rs12090194 in *n* = 38,811 individuals (**A** and **B**) and for the combined GWAS lead SNP rs1545300 in *n* = 38,806 individuals (**C** and **D**). The regional association plots (**A** and **C**) show the association results in a ±500-kb region around the lead SNP. SNPs are plotted on the *x* axis according to their chromosomal position with the –log_10_(*P* value) of the GWAS association on the *y* axis. Correlation with the lead SNP (purple) is estimated based on the 1000 Genomes reference samples. Plots were generated using the LocusZoom website (54). Genetic positions refer to GRCh37/hg19 coordinates. Forest plots of the respective lead SNPs are provided in **B** and **D**, with ORs and their 95% confidence intervals plotted on the *x* axis. I^2^ is the percentage of total variation across studies that is due to heterogeneity.

In the first stage, we performed a GWAS meta-analysis in up to 2,181 cases and 23,641 controls from 8 discovery cohorts. In total, 6,976,246 SNPs passed quality control (see Methods). We identified 19 variants spanning 49 kb in potassium voltage-gated channel subfamily D member 3 (*KCND3*) as well as rs139772527 (effect allele frequency [EAF] 1.4%, OR = 2.57, *P* = 2.0 × 10^–8^) near hemoglobin subunit zeta (*HBZ*) as being genome-wide significantly associated (*P* < 5 × 10^–8^) with ERP. The SNP with the lowest *P* value in the region (the lead SNP) at *KCND3* was the intronic rs12090194 (EAF 32.5%, OR = 0.80, *P* = 4.6 × 10^–10^), and this SNP was replicated in an independent sample of 1,124 cases and 12,510 controls from 4 additional cohorts (*P*_replication_ = 2.5 × 10^–3^, *P*_combined_ = 9.3 × 10^–12^; Table 2). The SNP rs139772527 near *HBZ* did not fulfil the criteria for replication (*P*_replication_ = 0.28, *P*_combined_ = 1.4 × 10^–6^; Table 2), as described in the Methods. The subsequent combined meta-analysis of all 12 cohorts, including up to 39,456 individuals, revealed only the locus at *KCND3* to be genome-wide significantly associated with ERP (Supplemental Figure 1; supplemental material available online with this article; https://doi.org/10.1172/jci.insight.131156DS1). The lead SNP of the combined GWAS meta-analysis was rs1545300 (EAF 31.9%, OR = 0.82, *P* = 7.7 × 10^–12^), followed by the discovery-stage lead SNP, rs12090194, which was in strong linkage disequilibrium (LD) with rs1545300 (*r*^2^ = 0.96, D′ = 1) (Figure 1). Both SNPs were imputed at very high confidence (imputation quality score >0.97) in all cohorts. The quantile-quantile plots did not show any inflation (individual study λ_GC_ between 0.81 and 1.03; median, 0.91; overall meta-analysis λ_GC_ = 1.02; LD score regression intercept, 1.01; see Methods) (Supplemental Figure 2). The result of the combined GWAS meta-analysis was used for the subsequent analyses. Summary statistics based conditional analysis to select independent hits did not reveal any secondary signals. The association results for each stage of the lead SNPs with *P* < 1 × 10^–6^ in the discovery meta-analysis are provided in Supplemental Table 1.

### Statistical fine mapping of the associated locus.

**Figure 2:**
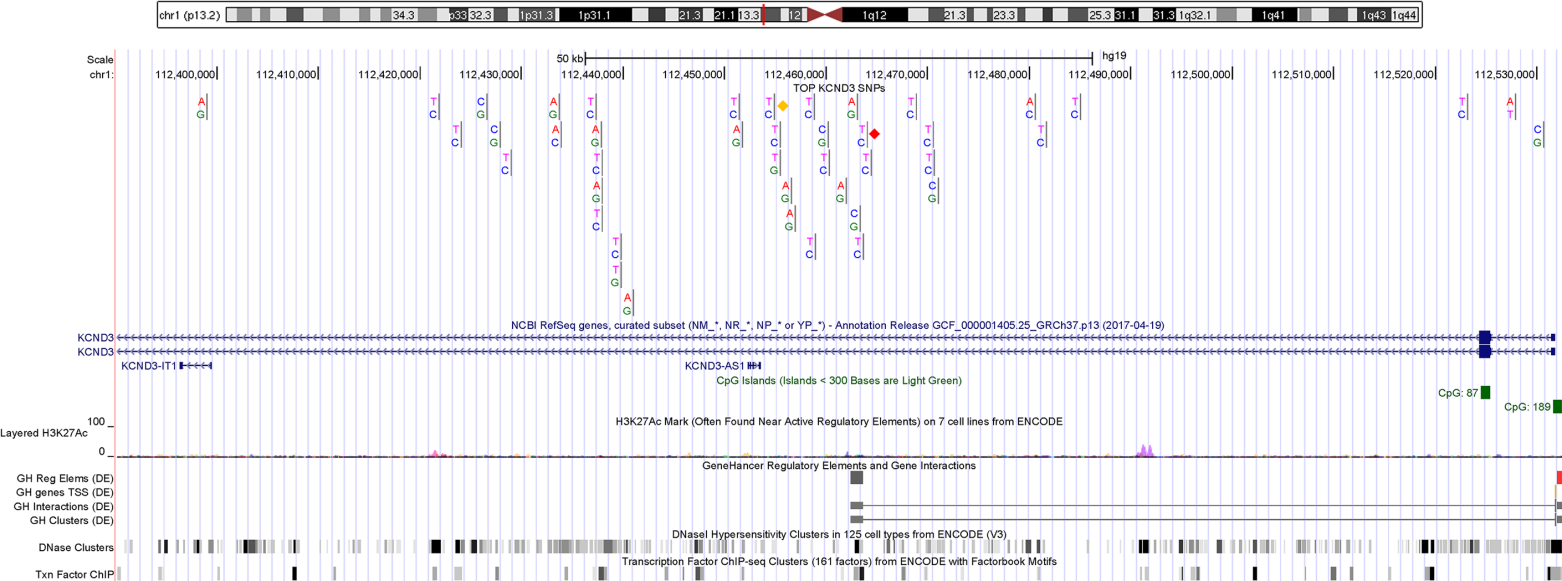
Location of the significantly (*P* < 5 × 10^–8^) associated SNPs within the *KCND3* gene. The top 43 SNPs with a genome-wide significance visualized by UCSC Genome Browser (55). All 43 SNPs mapped into the *KCND3* gene. The 2 leads SNPs, rs1545300 and rs12090194, of the discovery and combined meta-analyses are reported with a red and an orange diamond, respectively. The H3K27Ac mark track (Layered H3K27Ac) shows the levels of enrichment of the H3K27Ac histone mark. Chemical modifications (e.g., methylation and acylation) to the histone proteins present in chromatin influence gene expression by changing how accessible the chromatin is to transcription. The H3K27Ac histone mark is thought to enhance transcription, possibly by blocking the spread of the repressive histone mark H3K27Me3. The GeneHancer (GH) track set shows human regulatory elements, i.e., enhancers (gray) and promoters (red) containing tracks representing regulatory elements (Reg Elems), gene transcription start sites (TSS), associations between regulatory elements and genes (Interactions), and clustered interactions (Clusters). A gray box in the DNaseI hypersensitivity clusters track (DNase Clusters) indicates the extent of the hypersensitive region with darkness proportional to the maximum signal strength observed in any cell line. A gray box in the transcription factor ChIP-seq clusters track (Txn Factor ChIP) indicates a cluster of transcription factor occupancy, with the darkness of the box being proportional to the maximum signal strength observed in any cell line contributing to the cluster.

All significantly associated SNPs of the combined GWAS meta-analysis were located within *KCND3* and were intronic (Table 3 and Figure 2). We used these results to assess whether a single SNP or set of variants drive the association signal in *KCND3* (credible set). The 99% credible set was computed based on approximate Bayes factors for each SNP, resulting for each in a set of SNPs that with 99% posterior probability contained the variant(s) driving the association signal. For the associated locus at *KCND3* the credible set spanned 49 kb and contained 19 variants. The 2 lead SNPs, rs1545300 and rs12090194, had a posterior probability of 21% and 19%, respectively, whereas the former candidate SNP, rs17029069 (22), had a posterior probability of 2% (Supplemental Table 2).

To test whether the association in *KCND3* might be driven by heart rate or RR interval, we performed a sensitivity analysis in the 1,253 ERP cases and 11,463 controls of the Lifelines cohort, adjusting the genetic association of rs1545300 additionally for these 2 traits in separate models. The effect estimates were virtually unchanged (OR = 0.78), with *P* = 1.2 × 10^–7^ for both adjustments. In addition, we assessed whether the association of rs1545300 might be related to a specific ERP subtype, i.e., ST segment or ERP localization. In all subtype-stratified analyses, the 95% confidence intervals of the effect sizes overlapped with the overall results, which did not point to a subtype driven signal (Supplemental Table 3).

### Expression quantitative trait locus and colocalization.

We searched the Genotype-Tissue Expression (GTEx) project database (24) to look for tissue-specific expression quantitative trait loci (eQTLs), including all genes in the vicinity of ±1 Mb of the lead SNP, rs1545300, and found an association with *KCND3* expression levels in the tibial artery (*P* = 3.0 × 10^–6^, *n* = 388). Two additional eQTL associations of rs1545300, at FDR < 0.2, across the 48 tissues tested were found with *KCND3* (ENSG00000171385.5) in the left ventricle (*P* = 2.9 × 10^–4^, *n* = 272) of the human heart and with *CEPT1* (ENSG00000134255.9) in the minor salivary gland (*P* = 3.4 × 10^–4^, *n* = 85) (Supplemental Table 4).

**Figure 3:**
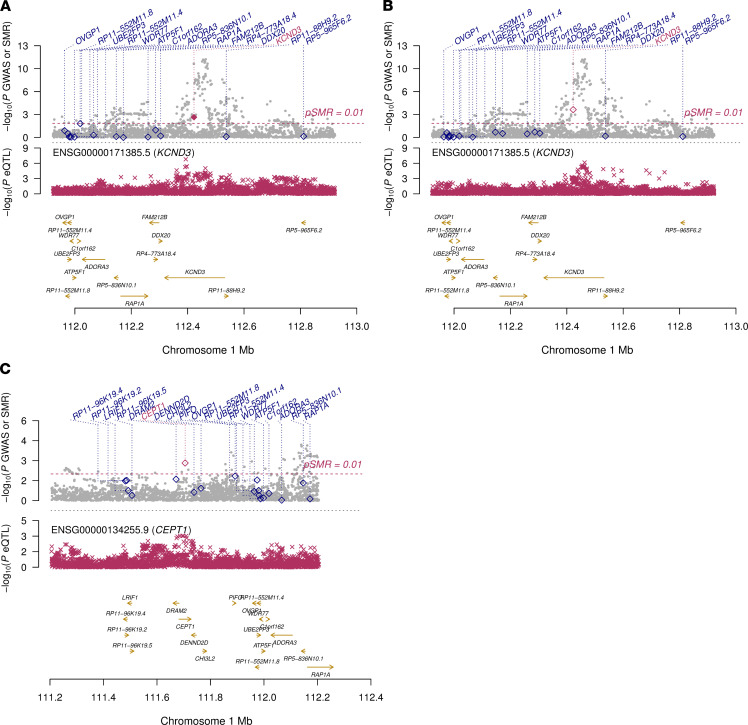
Colocalization results. Illustrations of the SMR test for the early repolarization pattern (ERP) risk and the expression quantitative trait loci (eQTLs) at the rs1545300 locus at chromosome 1p13.2 for the (**A**) left ventricle of the heart, (**B**) tibial artery, and (**C**) minor salivary gland tissue. The sample sizes for the eQTLs are *n* = 272, *n* = 388, and *n* = 85 in **A**, **B**, and **C**, respectively. In **A**–**C**, the GWAS regional association plot with ERP risk of the combined GWAS (*n* = 39,456), with level of significance of the SMR test (*y* axis) for each transcript in the locus indicated by a diamond positioned at the center of the transcript is shown (top). A significant SMR test represented by a purple diamond indicates an association of the transcript level of the respective genes (purple label) with the trait. For all 3 tissues, an increased gene expression level shown by a significant SMR test was associated with a higher risk of ERP. A filled purple diamond indicates a HEIDI test *P* > 0.05 and, thus, a likely colocalization. The regional association distribution with changes in expression of the highlighted (purple) gene transcript in the respective tissue is shown below. The *x* axis shows GRCh37/hg19 genomic coordinates throughout.

Subsequent colocalization analyses of rs1545300 in these 3 tissues revealed a significant correlation of gene expression pattern with ERP (*P*_SMR_ ≤ 0.01) (Figure 3 and Supplemental Table 5), where for the left ventricle the correlation seems to be attributable to the same underlying causative variant (*P*_HEIDI_ ≥ 0.05) and for tibial artery the test was close to nominal significance (*P*_HEIDI_ = 0.05). However, the significant *P*_HEIDI_ = 1.7 × 10^–3^ for *CEPT1* in the minor salivary gland points rather toward a pleiotropic effect of rs1545300 than a causal effect of gene expression on ERP in this tissue. For all 3 tissues, an increased gene expression level was associated with a higher risk of ERP (Supplemental Table 5).

### Pleiotropic effects of the lead SNPs.

To assess pleiotropic effects of the *KCND3* lead SNP rs1545300 or its proxies (*r*^2^ > 0.8), we looked for genome-wide significant associations in the NHGRI-EBI catalog of published GWAS (25) (accessed July 30, 2019). Pleiotropic associations were found for P wave terminal force (rs12090194 and rs4839185) (26) and for reduced risk of atrial fibrillation per minor allele (rs1545300 and rs1443926) (27, 28). All these SNPs were in strong LD (*r*^2^ > 0.97) with the lead SNP. In addition, variants in low-to-moderate LD with rs1545300 were associated with P wave duration (rs2798334, *r*^2^ = 0.26) (29) and ST-T wave amplitudes (rs12145374, *r*^2^ = 0.60) (30). A phenome-wide lookup of rs1545300 in the association results of 778 traits available via the Gene ATLAS web portal (31) using 452,264 individuals from the UK Biobank cohort revealed an association of the ERP risk–reducing minor T allele with reduced risk of heart arrhythmia (estimated OR = 0.92, *P* = 3.6 × 10^–6^). Of note, no other of the assessed traits reached significance after Bonferroni correction (*P* < 0.05 for the analyzed 778 traits = 6.4 × 10^–5^).

## Discussion

In this GWAS meta-analysis comprising 3,305 cases and 36,151 controls, including independent replication samples, we describe an association of ERP with a locus on chromosome 1 in the *KCND3* gene. This is the first study to our knowledge that identifies a robust genome-wide significant association between genetic variants and ERP. Our findings provide a candidate gene for further functional studies examining the pathophysiological mechanism of ERP and potentially ERS. The *KCND3* gene encodes the main pore-forming α subunit of the voltage-gated rapidly inactivating A-type potassium channel. In the cardiac ventricle *KCND3* contributes to the fast cardiac transient outward potassium current (I_to_), which plays a major role in the early repolarization phase 1 of the cardiac action potential (AP).

To date, two competing theories explain the presence of J waves and ERP: the repolarization and the depolarization theory; both of these involve the I_to_ channel. On the basis of animal models, evidence for the former is more compelling. Thus, J waves result from a transmural voltage gradient created by a more prominent epicardial phase 1 AP notch relative to the endocardial AP notch (11, 32). The I_to_ current notably influences the degree of the transmural heterogeneity of the phase 1 AP notch and consecutively the magnitude of the J wave (11, 32). Pharmacological inhibition of the I_to_ current with 4-aminopyridine results in a reduction of the J wave amplitude (11). The depolarization theory is based on clinical overlap of ERP with Brugada syndrome, which has led to the suggestion that Brugada syndrome is a right ventricular variant of the ERP (33). In theory, deviation from the sequential activation of cardiac currents I_Na_, I_to_, and I_CaL_ can lead to regional conduction slowing and appearance of inferior and/or lateral ERP (32, 34). In patients with ERS, distinct phenotypes of both delayed depolarization and early repolarization have been identified (35).

ERP is a highly heritable trait within families (3, 20); however, limited heritability can be attributed to common SNPs in unrelated individuals (21). This might be a reason why the only GWAS to date that included 452 cases failed to replicate any genome-wide significant loci (22). In our study, which includes 3,334 cases, we discovered and replicated variants in the *KCND3* gene. Interestingly, one of these variants (rs17029069), which is in moderate LD (*r*^2^ = 0.18, D′ = –1) with our lead SNP, rs1545300 (Supplemental Figure 3), was reported as a candidate in the earlier GWAS meta-analysis (22). However, this variant did not replicate in their study; the authors attributed this finding to limited power based on the small sample size and/or heterogeneous phenotyping. In our study, experienced cardiologists evaluated more than 39,000 ECGs with high reproducibility ensuring a very high phenotyping quality (21). The resulting homogenously assessed phenotype and the substantially increased number of cases are two aspects that elevated the statistical power of our GWAS meta-analysis. All detected variants cluster in intronic regions of the *KCND3* gene, without significant allelic heterogeneity. The annotation of the locus does not point to a direct pathogenic effect, i.e., a protein-altering mutation; additionally, the statistical fine mapping revealed no single SNP with a substantial posterior probability (e.g., >80%) of being causal. However, the latter approach has limitations in detecting rare causal variants due to imputation uncertainty and minimum minor allele frequency (MAF). Nevertheless, eQTL analysis suggested that the detected variants may affect *KCND3* gene expression. Potential mechanisms include modification of gene expression via altered binding of transcription factors at *cis*-elements through enhancers or in DNaseI hypersensitivity regions (Figure 2). This is supported by the results of the test for colocalization, which showed an increase of ERP risk due to increased *KCND3* gene expression levels in tissues of the human heart and tibial artery. Similar, pharmacological ex vivo data predict that gain-of-function mutations in the I_to_ current increase the overall transmural outward shift, leading to an increased epicardial AP notch and thereby inducing ERP in the surface ECG (32). Additionally, a long noncoding RNA (lncRNA), KCND3 antisense RNA 1 (*KCND3-AS1*), has been described to be in close proximity to the lead SNP, rs1545300 (Figure 2). lncRNAs have been shown to physiologically influence gene regulation through various mechanisms, e.g., chromatin remodeling, control of transcription initiation, and posttranscriptional processing (36, 37). On the other hand, dysregulation of lncRNA control circuits can potentially effect the development of disease (38): a very prominent example of this in cardiovascular diseases is the lncRNA *ANRIL*, which is a key effector of *9p21* in atherosclerotic risk and cardiovascular events (38–40).

Given the high prevalence of ERP in the general population and a high MAF of the identified genetic variants in our study the key question remains of why only a very small subset of individuals develops severe ventricular arrhythmias and ERS. The fine interplay of a genetic predisposition and specific precipitating conditions might lead to an electrically vulnerable cardiac state. Insights into the potential origin of ventricular arrhythmias in ERS come from animal models and highlight the role of different ion channels, including I_to_ (10). A pharmacological model of ERS in canine wedges from the inferior and lateral ventricular wall showed marked regional dispersion of repolarization (loss of phase 2 AP dome and AP shortening in some epicardial regions but not others). Presence of transmural repolarization heterogeneity allowed local reexcitation in form of closely coupled extrasystolic activity (phase 2 reentry). The combination of an arrhythmogenic substrate, represented by regional electrical instability, and triggering premature ventricular beats resulted in ventricular fibrillation (10). Data from ERS patients suggest that, in a subgroup, the ERP is due to a pure repolarization phenotype and arrhythmia (35) is triggered by Purkinje fiber ectopic beats.

Genetic variants in various ion channel genes have been associated with ERS (16), including the *KCNJ8* and *ABCC9* genes encoding the Kir6.1- and ATP-sensing subunits of the K_ATP_ channel (6, 12, 41, 42). The commonly implicated variant KCNJ8-p.S422L has a population frequency not consistent with ERS and is predicted to be benign by multiple in silico algorithms according to the ClinVar database (43). A recent study by Chauveau et al. has, however, identified a de novo duplication of the *KCND3* gene in a patient who survived sudden cardiac death and in his 2-year-old daughter (13). Both exhibited marked ERP in the inferolateral leads that was augmented by bradycardia and pauses in heart rhythm, in keeping with a repolarization mechanism underlying the ERS phenotype. Studies have suggested that the inferior region of the left ventricle has a higher density of *KCND3* expression and higher intrinsic levels of I_to_ (10). This may explain the higher vulnerability of this region for the development of ERS in the setting of a genetically mediated gain of function in the I_to_ current. Moreover, observational studies also identified different ERP subtypes, including the occurrence of ERP in the inferior region and a horizontal/descending ST segment morphology associated with a higher risk of sudden arrhythmic death and cardiovascular mortality (2, 44, 45). However, in a subgroup of our study, the association signal of ERP risk and *KCND3* variation was not dominated by a specific ERP high-risk subtype. Of note, the formation of subgroups led to reduction in sample size and thus statistical power.

Taken together, the rare occurrence of ERS may be explained by different conditions. On the one hand, an underlying monogenic mutation may be found in some cases. On the other, no single causal mutation can be identified in the majority of ERS cases, rendering the influence of multiple genes and environmental factors more likely, i.e., a “multihit condition.” Similar to other polygenic diseases, the sum of multiple minor effects of several common genetic variations together with specific external triggers may affect the occurrence of ERS. There is indeed evidence to suggest that common variants in the *KCND3* locus increase arrhythmogenicity. A phenome-wide lookup of our common lead SNP in more than 450,000 individuals from the UK Biobank linked the minor T allele associated with reduced ERP to a reduced risk of heart arrhythmia (31). Furthermore, additional data show an association of the same common variant with reduced risk of atrial fibrillation (27, 28). A small effect of a common SNP at *KCND3* does not necessarily mean that the variant is benign; rather, a single risk allele is associated with a small but effective change in the gene expression level. Thus, the overall effects of the *KCND3* gene expression levels on the phenotype may appear much stronger compared with the small effect of rs1545300. Based on our results, it could be hypothesized that variation in *KCND3* gene expression levels and subsequently its encoded protein may affect the risk of ERP and eventually ERS. The positive effect direction of the change in *KCND3* gene expression levels in heart tissue on the risk of ERP estimated via the SMR test (Supplemental Table 5) suggested an elevated risk, with increasing abundance of the *KCND3* encoded protein. Functional validation is necessary to validate this hypothesis, and analyses of the *KCND3* gene in individuals with ERS are warranted to confirm the role of *KCND3* variation in arrhythmogenesis.

Our study has some limitations that need to be acknowledged. The presence of ERP in the ECG can be variable, as it has been described to be dependent on age, heart rate, vagal activity, and medication, although our findings were valid after adjusting for some of these factors. Therefore, we cannot exclude that we have missed some individuals with ERP. Second, the tissue-specific gene expression data used for the colocalization analysis are based on a limited sample size. A larger gene expression sample or functional studies are needed to validate the revealed effect of *KCND3* expression on the ERP. In addition, we analyzed only common and low-frequency SNPs with a MAF >1% missing rare variants and variants not included in the imputation panel. Finally, long-term outcome data identifying those individuals with ERP who suffer from ERS are not available. Further GWAS in large international collaborative cohorts of ERS patients are therefore necessary to determine the genetic risk.

In conclusion, we show for the first time to our knowledge, a robust association of genetic variants with the ERP in a large GWAS of individuals of European ancestry. The locus in the *KCND3* ion channel gene is an intuitive candidate and supports the theory that at least a proportion of ERS is a pure channelopathy. Intensive future research will be needed to extend the discovery of ERP susceptibility loci to individuals of non-European ancestry and to improve identification and risk stratification of the subset of individuals with the ERP who are at highest risk for potentially lethal ventricular arrhythmias.

## Methods

### Study cohorts and SNP genotyping.

The discovery stage included 25,822 subjects (2,181 ERP cases) from 8 independent cohorts with genetic and phenotypic data available for analyses: the British Genetics of Hypertension (BRIGHT) study, the Gutenberg Health Study (GHS1, GHS2), the Genetic Regulation of Arterial Pressure In humans in the Community (GRAPHIC) study, the Lifelines Cohort Study, the Study of Health in Pomerania (SHIP, SHIP-Trend), and TwinsUK. An additional 13,634 subjects (1,124 ERP cases) from 4 cohorts (Rotterdam Study I, II, III, and Cooperative Health Research In South Tyrol [CHRIS] study) were used for independent replication: the Rotterdam Study (Rotterdam Study I, II, III) and the CHRIS study. The included subjects of all cohorts were of European ancestry, and all cohorts but BRIGHT (which sampled hypertensive cases) were population based (Supplemental Table 6). The determination of the discovery and replication cohorts was determined upfront based on the timeline of the availability of the genetic and ERP data.

### ECG analysis and ERP evaluation.

Twelve-lead ECGs of all twelve studies were obtained during a study visit in a supine position after approximately 5 minutes of rest and were analyzed manually by experienced and specifically trained cardiologists for the presence of ERP. In detail, ECGs from TwinsUK and BRIGHT were evaluated in the United Kingdom, and ECGs from all other cohorts were evaluated in Germany. Paper-printed 12-lead ECGs were independently read by 2 experienced clinicians who were blinded with respect to age and sex. There was very high level of agreement between each pair of interpreters (95%–98%) (20, 21). Cases of ambiguous or unequal phenotype were jointly reassessed by 2 readers, and a consensus decision was achieved. To determine interobserver variability between the United Kingdom and German teams, a subset of ECGs was analyzed by both teams, yielding a concordance of 96% (20, 21).

The ERP phenotype was established according to the definition by Haïssaguerre and Macfarlane (6, 23). ERP was defined as elevation of the J point above the level of QRS onset of ≥0.1 mV in at least 2 corresponding leads. To avoid confusion or overlap with Brugada syndrome or arrhythmogenic right ventricular dysplasia, leads V1–V3 were excluded from ERP scoring. In case of presence of ERP, region (inferior: leads II, III, aVF; anterolateral: leads I, aVL; V_4_–V_6_, or both) and the maximum amplitude of J point elevation were documented. Further, the morphology of ERP was assessed as notching, slurring, or both; additionally, the ST segment was assessed according to Tikkanen and colleagues (44) as either concave/rapidly ascending (>0.1 mV elevation 100 ms after J point peak or persistently elevated ST segment >0.1 mV) or horizontal/descending (≤0.1 mV elevation within 100 ms after J point peak) (23, 44). In case of a QRS duration of >120 ms or rhythm other than sinus rhythm (e.g., atrial fibrillation, pacemaker stimulation) ECGs were excluded from the analysis.

### GWAS in individual studies.

The GWAS in each study for both the discovery and replication stage was performed on autosomal imputed SNP genotypes using study-specific quality control protocols that are provided in detail in Supplemental Table 6. Association analyses were performed using logistic regression for ERP status as outcome and an additive genetic model on SNP dosages, thus taking genotype uncertainties of imputed SNPs into account. The analyses were adjusted for age, sex, and relevant study-specific covariates, such as principal components for population stratification (Supplemental Table 6).

### Meta-analysis of individual study GWAS results.

The result files from individual cohorts’ GWAS underwent extensive quality control before meta-analysis using the gwasqc() function of the GWAtoolbox package v2.2.4 (46, 47). The quality control included file format checks as well as plausibility and distributions of association results, including effect sizes, standard errors, allele frequencies, and imputation quality of the SNPs.

The meta-analyses were conducted using a fixed-effect inverse variance weighting as implemented in Metal (48). Monomorphic SNPs, SNPs with implausible association results (i.e., *P* ≤ 0, SE ≤ 0, |log(OR)| ≥ 1000), and SNPs with an imputation quality score ≤0.4 were excluded prior to the meta-analyses, resulting in a median of 12,839,202 SNPs per cohort (IQR: 10,756,073–13,184,807). During the meta-analysis, the study-specific results were corrected by their specific λ_GC_ if > 1. Results were checked for possible errors like use of incorrect association model by plotting the association *P* values of the analyses against those from a *Z* score based meta-analysis for verifying overall concordance. SNPs that were present in <75% of the total sample size contributing to the respective meta-analysis or with a MAF ≤0.01 were excluded from subsequent analyses. Finally, data for up to 6,976,246 SNPs were available after the meta-analysis.

Quantile-quantile plots of the meta-analysis results are provided in Supplemental Figure 2. To assess whether there was an inflation of *P* values in the meta-analysis results attributed to reasons other than polygenicity, we performed LD score regression (49). The LD score–corrected λ_GC_ value of the discovery and replication combined meta-analysis was 1.01, supporting the absence of unaccounted population stratification. Genome-wide significance was defined as *P* < 5 × 10^–8^, corresponding to a Bonferroni correction of 1 million independent tests (50). The I^2^ statistic was used to evaluate between-study heterogeneity (51).

To evaluate the presence of allelic heterogeneity within each locus, the GCTA stepwise model selection procedure (cojo-slct algorithm) was used to identify independent variants using a step-wise forward selection approach (52). We used the genotype information of 4,081 SHIP individuals for LD estimation and set the significance threshold for independent SNPs to 5 × 10^–8^.

All loci were named according to the nearest gene of the lead SNP. Genomic positions correspond to build 37 (GRCh37).

### Replication analysis.

To minimize the burden for multiple testing correction and thus maximizing the power for replication, the lead SNPs of genome-wide significant loci in the discovery stage were taken forward to the replication stage in independent samples (Table 1). SNPs were considered as replicated if the *P* value of a 1-sided association test was <0.025, which corresponds to a Bonferroni correction for the 2 lead SNPs tested at 5% significance level.

Finally, the GWAS results from the discovery and replication studies were meta-analyzed to search for additional genome-wide significant loci by maximizing the statistical power for locus discovery.

### Gene expression–based analyses.

The lead SNP, rs1545300, of the *KCND3* locus of the combined discovery and replication GWAS meta-analysis was tested for *cis* eQTLs (±1-Mb window around the transcription start site) in 48 tissues available in the GTEx v7 database that included at least 70 samples. Significant associations were selected based on a Bonferroni-corrected *P* < 3.0 × 10^–5^ for the number of genes and tissues tested.

Subsequently, the SNP rs1545300 was tested and plotted for colocalization in the 3 tissues with an eQTL FDR < 0.2 by applying the SMR method (53) using the GWAS and GTEx eQTL summary statistics. The method includes a test of whether the effect on expression observed at a SNP or at its proxies is independent of the signal observed in the GWAS, i.e., that gene expression and *y* are associated only because of a latent nongenetic confounding variable (SMR test), and a second test that evaluates if the eQTL and GWAS associations can be attributable to the same causative variant (HEIDI test). Significance for colocalization of gene expression and the GWAS signals was defined by *P*_SMR_ < 0.01, where additionally a *P*_HEIDI_ ≥ 0.05 indicates the same underlying causal variant (53).

### Data availability.

Summary association results of the combined GWAS meta-analysis have been submitted for full download to the CHARGE dbGaP website under accession phs000930 (https://www.ncbi.nlm.nih.gov/gap).

### Statistics.

Unless stated otherwise, the analyses were conducted and plotted using R statistical software (46), a *Z* test was applied, and all reported *P* values are 2 sided. *P* values of less 0.05 after correction for multiple testing were considered significant.

### Study approval.

The BRIGHT study ethics committee approval was obtained from the multiple local research committees of the partner institutes from Aberdeen, Glasgow, London, Cambridge, and Oxford in the United Kingdom. The GHS study followed the recommendations of the Declaration of Helsinki and approval was obtained by the local data protection officer and ethics committee of the Chamber of Physicians of Rhineland-Palatinate, Germany (reference no. 837.020.07). For the GRAPHIC Study, the Leicestershire Research Ethics Committee approved the study, and all subjects provided written, informed consent. The LifeLines Cohort Study is conducted according to the principles of the Declaration of Helsinki and in accordance with the research code of the University Medical Center Groningen (UMCG). The LifeLines study is approved by the medical ethical committee of the UMCG, the Netherlands. The medical ethics committee of the University of Greifswald approved the SHIP/SHIP-Trend study protocol, and oral and written informed consents were obtained from each of the study participants. The TwinsUK study has ethical approval from Guys & St Thomas’ Trust Ethics Committee. The CHRIS study has been approved by the “Ethikkomitee Klinische Prüfung - Gesundheitsbezirk Bozen, Bozen, Südtirol.” The Rotterdam Study has been approved by the Medical Ethics Committee of the Erasmus MC (registration no. MEC 02.1015) and by the Dutch Ministry of Health, Welfare and Sport (Population Screening Act WBO, license no. 1071272-159521-PG). The Rotterdam Study has been entered into the Netherlands National Trial Register (NTR; https://www.trialregister.nl/) and into the WHO International Clinical Trials Registry Platform (https://www.who.int/ictrp/network/primary/en/) under shared catalog no. NTR6831. All participants provided written informed consent to participate in the study and to have their information obtained from treating physicians.

## Author contributions

WR, TT, AT, MD, and ERB were responsible for project design and analysis. AFD, CF, CP, CPN, ERB, KJL, LMH, MAI, MD, MG, MK, MZ, NJS, NP, NV, PBM, PPP, PSB, PSW, PVDH, RB, RBS, SA, SBF, TDS, TM, TT, WR, and YJ managed the individual studies. AFD, GS, H. Schunkert, ID, MD, MZ, NV, PVDH, SA, and SBF recruited individual study subjects. AR, AT, BHS, ERB, MDB, MEVDB, TT, and WR drafted the manuscript. AT, CC, CF, CM, HRW, H. Snieder, IMN, KS, MEVDB, and SP were responsible for statistical methods and analysis of the study. AGU, CF, MN, PBM, and UV were responsible for genotyping of the individual studies. AR, AT, BK, CH, ERB, MD, MDB, TK, TT, and WR interpreted results. All authors engaged in critical review of the manuscript. The authorship order among co–first authors was set alphabetically.

## Supplementary Material

Supplemental data

ICMJE disclosure forms

## Figures and Tables

**Table 3:**
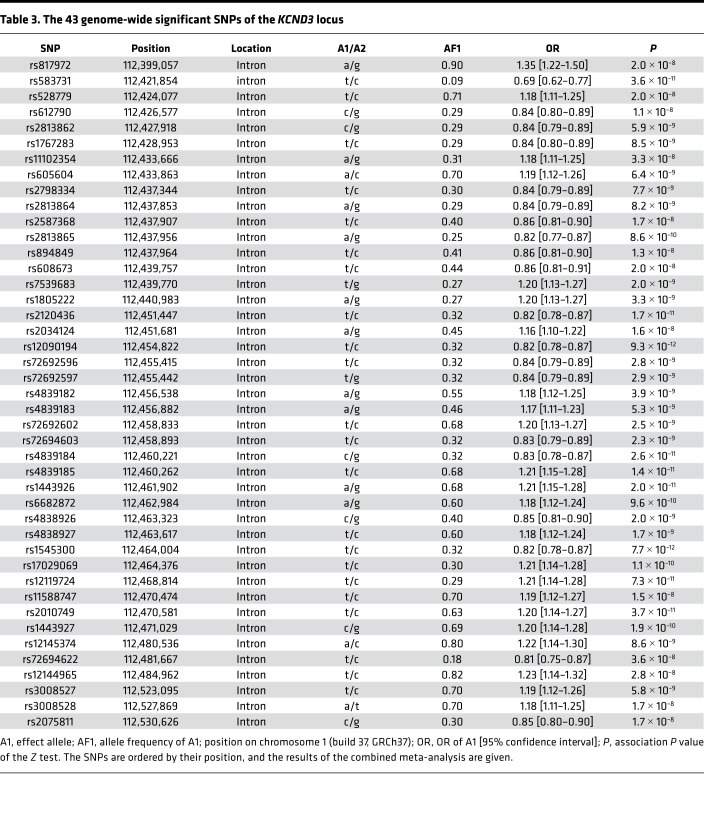
The 43 genome-wide significant SNPs of the *KCND3* locus

**Table 2:**
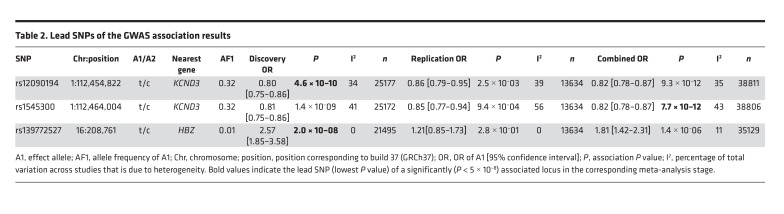
Lead SNPs of the GWAS association results

**Table 1:**
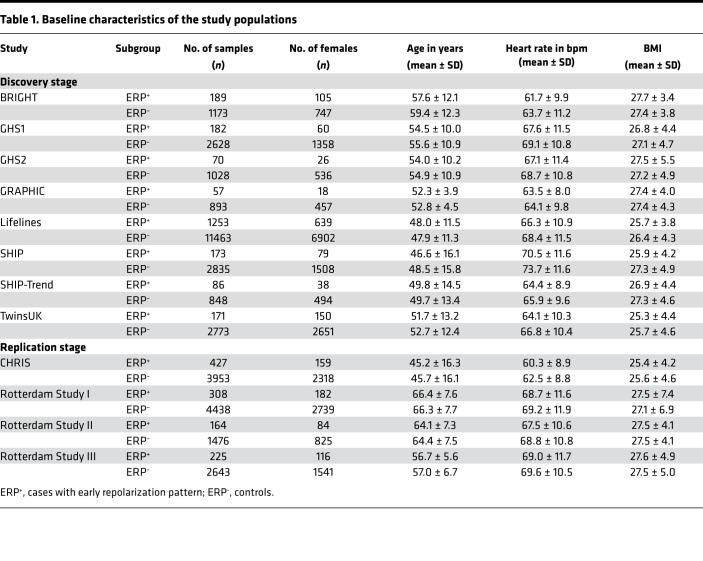
Baseline characteristics of the study populations

## Version 1 10/10/2019

In-Press Preview

## Version 2 12/05/2019

Electronic publication

## Version 3 08/29/2020

The copyright information was updated.
