# Supplementary material for: KCND3 potassium channel gene variant confers susceptibility to electrocardiographic early repolarization pattern
Source: JCI Insight. 2019 Dec 5;4(23):e131156. doi: 10.1172/jci.insight.131156 (PMC6962032; doi:10.1172/jci.insight.131156)
Supplement: Supplemental data [file jciinsight-4-131156-s007.pdf]

KCND3 potassium channel gene variant confers susceptibility to electrocardiographic early repolarization pattern

SUPPLEMENTARY INFORMATION

Table of Contents

SUPPLEMENTARY TEXT 3

ACKNOWLEDGMENTS 3

SUPPLEMENTARY TABLES 6

SUPPLEMENTARY TABLE 1: LOCI WITH LEAD SNP P<1X10^{-6} IN THE DISCOVERY STAGE 6
SUPPLEMENTARY TABLE 2: VARIANTS OF THE KCND3 LOCUS MAPPING INTO A 99% CREDIBLE SET 7
SUPPLEMENTARY TABLE 3: ASSOCIATION RESULTS OF RS1545300 STRATIFIED BY ERP SUBTYPES IN THE LIFELINES COHORT 7
SUPPLEMENTARY TABLE 4: GTEX CIS EQTL LOOKUP RESULTS OF RS1545300 ACROSS 48 TISSUES 8
SUPPLEMENTARY TABLE 5: CO-LOCALIZATION RESULTS FOR ALL TISSUES WITH SIGNIFICANT CIS EQTL ASSOCIATIONS OF RS1545300 45
SUPPLEMENTARY TABLE 6: STUDY GENOTYPING INFORMATION 48

SUPPLEMENTARY FIGURES 51

SUPPLEMENTARY FIGURE 1: MANHATTAN PLOT OF THE COMBINED GWAS META-ANALYSIS 51
SUPPLEMENTARY FIGURE 2: QQ PLOT OF THE COMBINED GWAS META-ANALYSIS 52
SUPPLEMENTARY FIGURE 3: REGIONAL ASSOCIATION PLOT OF RS17029069 53

Supplementary Text

Acknowledgments

The Genotype-Tissue Expression (GTEx) Project was supported by the Common Fund of the Office of the Director of the National Institutes of Health, and by NCI, NHGRI, NHLBI, NIDA, NIMH, and NINDS.

BRIGHT

The BRIGHT study is extremely grateful to all the patients who participated in the study and the BRIGHT nursing team. This works forms part of the research areas contributing to the translational research portfolio of the Cardiovascular Biomedical Research Centre at Barts which is supported and funded by the National Institute for Health Research (Warren, Cabrera, Munroe). This work was supported by the Medical Research Council of Great Britain (grant number G9521010D) and the British Heart Foundation (grant number PG/02/128).

CHRIS

Full acknowledgements for the Cooperative Health Research In South Tyrol (CHRIS) study are reported here: http://translational-medicine.biomedcentral.com/articles/10.1186/s12967-015-0704-9#Declarations. The CHRIS study was funded by the Department of Innovation, Research, and University of the Autonomous Province of Bolzano-South Tyrol.

GHS

Gutenberg Health Study: The Gutenberg Health Study is funded through the government of Rhineland-Palatinate („Stiftung Rheinland-Pfalz für Innovation“, contract AZ 961-386261/733), the research programs “Wissen schafft Zukunft” and “Center for Translational Vascular Biology (CTVB)” of the Johannes Gutenberg-University of Mainz, and its contract with Boehringer Ingelheim and PHILIPS Medical Systems, including an unrestricted grant for the Gutenberg Health Study. Philipp S. Wild is funded by the Federal Ministry of Education and Research (BMBF 01EO1503) and he is PI of the German Center for Cardiovascular Research (DZHK). This project has received funding from the European Research Council (ERC) under the European Union’s Horizon 2020 research and innovation programme (grant agreement No 648131). This work was performed in the context of the Junior Research Alliance symAtrial project funded by the German Ministry of Research and Education (BMBF 01ZX1408A) e:Med – Systems Medicine program (RBS). RBS was supported by Deutsche Forschungsgemeinschaft (German Research Foundation) Emmy Noether Program SCHN 1149/3-1.

GRAPHIC

The GRAPHIC study was funded by the BHF.

Lifelines

Lifelines is a multi-disciplinary prospective population-based cohort study examining in a unique three-generation design the health and health-related behaviors of 167,729 persons living in the North of The Netherlands. It employs a broad range of investigative procedures

in assessing the biomedical, socio-demographic, behavioral, physical and psychological factors which contribute to the health and disease of the general population, with a special focus on multi-morbidity and complex genetics.

The Lifelines Cohort Study, and generation and management of GWAS genotype data for the Lifelines Cohort Study is supported by the Netherlands Organization of Scientific Research NWO (grant 175.010.2007.006), the Economic Structure Enhancing Fund (FES) of the Dutch government, the Ministry of Economic Affairs, the Ministry of Education, Culture and Science, the Ministry for Health, Welfare and Sports, the Northern Netherlands Collaboration of Provinces (SNN), the Province of Groningen, University Medical Center Groningen, the University of Groningen, Dutch Kidney Foundation and Dutch Diabetes Research Foundation.

The authors wish to acknowledge the services of the Lifelines Cohort Study, the contributing research centers delivering data to Lifelines, and all the study participants.

Lifelines Cohort Study group authors:

Behrooz Z Alizadeh (1), H Marike Boezen (1), Lude Franke (2), Pim van der Harst (3), Gerjan Navis (4), Marianne G Rots (5), Harold Snieder (1), Morris Swertz (2), Bruce HR Wolffenbuttel (6), Cisca Wijmenga (2)

(1) Department of Epidemiology, University of Groningen, University Medical Center Groningen, The Netherlands
(2) Department of Genetics, University of Groningen, University Medical Center Groningen, The Netherlands
(3) Department of Cardiology, University of Groningen, University Medical Center Groningen, The Netherlands
(4) Department of Internal Medicine, Division of Nephrology, University of Groningen, University Medical Center Groningen, The Netherlands
(5) Department of Pathology and Medical Biology, University of Groningen, University Medical Center Groningen, The Netherlands
(6) Department of Endocrinology, University of Groningen, University Medical Center Groningen, The Netherlands

<u>*Rotterdam Study*</u>

The generation and management of GWAS genotype data for the Rotterdam Study (RS I, RS II, RS III) was executed by the Human Genotyping Facility of the Genetic Laboratory of the Department of Internal Medicine, Erasmus MC, Rotterdam, The Netherlands. The GWAS datasets are supported by the Netherlands Organisation of Scientific Research NWO Investments (nr. 175.010.2005.011, 911-03-012), the Genetic Laboratory of the Department of Internal Medicine, Erasmus MC, the Research Institute for Diseases in the Elderly (014-93-015; RIDE2), the Netherlands Genomics Initiative (NGI)/Netherlands Organisation for Scientific Research (NWO) Netherlands Consortium for Healthy Aging (NCHA), project nr. 050-060-810. We thank Pascal Arp, Mila Jhamai, Marijn Verkerk, Lizbeth Herrera and Marjolein Peters, MSc, and Carolina Medina-Gomez, MSc, for their help in creating the GWAS database, and Karol Estrada, PhD, Yurii Aulchenko, PhD, and Carolina Medina-Gomez,

MSc, for the creation and analysis of imputed data. The Rotterdam Study is funded by Erasmus Medical Center and Erasmus University, Rotterdam, Netherlands Organization for the Health Research and Development (ZonMw), the Research Institute for Diseases in the Elderly (RIDE), the Ministry of Education, Culture and Science, the Ministry for Health, Welfare and Sports, the European Commission (DG XII), and the Municipality of Rotterdam. The authors are grateful to the study participants, the staff from the Rotterdam Study and the participating general practitioners and pharmacists.

SHIP

The Study of Health in Pomerania (SHIP) is part of the Community Medicine Research net of the University of Greifswald, Germany, which is funded by the Federal Ministry of Education and Research (grants no. 01ZZ9603, 01ZZ0103, and 01ZZ0403), the Ministry of Cultural Affairs as well as the Social Ministry of the Federal State of Mecklenburg-West Pomerania, and the network 'Greifswald Approach to Individualized Medicine (GANI_MED)' funded by the Federal Ministry of Education and Research (grant 03IS2061A). Genome-wide data have been supported by the Federal Ministry of Education and Research (grant no. 03ZIK012) and a joint grant from Siemens Healthineers, Erlangen, Germany and the Federal State of Mecklenburg- West Pomerania. The University of Greifswald is a member of the Caché Campus program of the InterSystems GmbH.

TwinsUK

The study was funded by the Wellcome Trust; European Community's Seventh Framework Programme (FP7/2007-2013). The study also receives support from the National Institute for Health Research (NIHR) BioResource Clinical Research Facility and Biomedical Research Centre based at Guy's and St Thomas' NHS Foundation Trust and King's College London and the British Heart Foundation. Tim Spector is holder of an ERC Advanced Principal Investigator award. SNP Genotyping was performed by The Wellcome Trust Sanger Institute and National Eye Institute via NIH/CIDR. Statistical analyses were carried out on the Genetic Cluster Computer (http://www.geneticcluster.org) hosted by SURFsara and financially supported by the Netherlands Scientific Organization (NWO 480-05-003 PI: Posthuma) along with a supplement from the Dutch Brain Foundation and the VU University Amsterdam. Y.J. received funding for the project from the British Heart Foundation (PG/12/38/29615 and PG/06/094/21278). E.B. receives funding from the Robert Lancaster Memorial Fund sponsored by McColl's RG Ltd.

Supplementary Tables

Supplementary Table 1: Loci with lead SNP $p<1x10^{-6}$ in the discovery stage

									Discovery stage					
**lead SNP**	**A1**	**A2**	**AF1**	**chr**	**position (GRCh37)**	**nearest gene**	**gene dist**	**SNP function**	**Effect**	**SE**	**OR**	**P**	**I²**	**N**
rs12090194	t	c	0.32	1	112,454,822	*KCND3*	0	intron	-0.2205	0.0354	0.80	4.6E-10	34.1	25177
rs139772527	t	c	0.01	16	208,761	*HBZ*	4257	intergenic	0.9447	0.1684	2.57	2.0E-08	0.0	21495
rs79630422	a	t	0.98	20	48,505,749	*SLC9A8*	0	untrans-3	-0.7406	0.1383	0.48	8.5E-08	0.0	21495
rs111637248	a	g	0.96	5	67,351,181	*PIK3R1*	160402	intergenic	-0.4761	0.0893	0.62	9.7E-08	0.0	21495
rs61588349	t	g	0.99	18	53,809,471	*LOC100505474*	4704	intron	-0.9616	0.1827	0.38	1.4E-07	0.0	21495
rs344507	a	g	0.02	5	35,029,862	*AGXT2*	0	intron	0.5921	0.1138	1.81	1.9E-07	18.8	25807
rs181993557	a	g	0.02	6	117,032,161	*KPNA5*	0	intron	0.6528	0.1306	1.92	5.8E-07	0.0	21495
rs2545583	a	g	0.33	5	102,048,098	*PAM*	153428	intergenic	-0.2301	0.0469	0.79	9.3E-07	2.5	21495
rs28453057	a	c	0.04	19	11,436,339	*RAB3D*	0	intron	0.4157	0.0848	1.52	9.4E-07	0.0	21495
rs76495268	t	c	0.71	10	5,219,864	*AKR1CL1*	0	intergenic	-0.221	0.0451	0.80	9.5E-07	22.9	21495

			Replication stage						Combined meta-analysis					
**lead SNP**	**A1**	**A2**	**Effect**	**SE**	**OR**	**P**	**I²**	**N**	**Effect**	**SE**	**OR**	**P**	**I²**	**N**
rs12090194	t	c	-0.1468	0.0485	0.86	2.5E-03	38.6	13634	-0.1949	0.0286	0.82	9.3E-12	35.3	38811
rs139772527	t	c	0.1935	0.1802	1.21	2.8E-01	0.0	13634	0.5945	0.1231	1.81	1.4E-06	17.8	35129
rs79630422	a	t	-0.2254	0.1933	0.80	2.4E-01	35.5	13634	-0.5662	0.1125	0.57	4.8E-07	11.8	35129
rs111637248	a	g	0.1578	0.1426	1.17	2.7E-01	0.0	13634	-0.2975	0.0757	0.74	8.4E-05	55.9	35129
rs61588349	t	g	0.0536	0.2161	1.06	8.0E-01	0.0	13634	-0.5382	0.1395	0.58	1.1E-04	47.4	35129
rs344507	a	g	-0.0607	0.1756	0.94	7.3E-01	0.0	13634	0.3991	0.0955	1.49	2.9E-05	47.8	39441
rs181993557	a	g	0.3078	0.4083	1.36	4.5E-01	79.2	13634	0.6208	0.1244	1.86	6.0E-07	50.7	35129
rs2545583	a	g	-0.0927	0.0449	0.91	3.9E-02	0.0	13634	-0.1584	0.0324	0.85	1.0E-06	28.0	35129
rs28453057	a	c	0.1355	0.1333	1.15	3.1E-01	67.1	13634	0.3351	0.0715	1.40	2.8E-06	31.8	35129
rs76495268	t	c	-0.0815	0.0482	0.92	9.1E-02	0.0	13634	-0.156	0.0329	0.86	2.2E-06	27.7	35129

lead SNP: SNP with the lowest p-value in the discovery stage of that region. A1: effect allele; AF1: allele frequency of A1; gene dist: distance of SNP in bases to nearest gene; Effect: association effect (log OR) of A1; SE: standard error of the effect; OR: odds ratio; P: association p-value; I^2: percentage of total variation across studies that is due to heterogeneity; N: sample size

Supplementary Table 2: Variants of the KCND3 locus mapping into a 99% credible set

SNP	chr	position (GRCh37)	A1	A2	AF1	Effect	SE	OR	P	I²	N	nearest gene	function	PP	cumulated PP
rs1545300	1	112,464,004	T	C	0.32	-0.1961	0.0287	0.82	7.7E-12	43.4	38806	*KCND3*	intron	0.215	0.215
rs12090194	1	112,454,822	T	C	0.32	-0.1949	0.0286	0.82	9.3E-12	35.3	38811	*KCND3*	intron	0.193	0.408
rs4839185	1	112,460,262	T	C	0.68	0.1929	0.0285	1.21	1.4E-11	38.6	39293	*KCND3*	intron	0.146	0.554
rs2120436	1	112,451,447	T	C	0.32	-0.1938	0.0288	0.82	1.7E-11	31.2	38805	*KCND3*	intron	0.112	0.666
rs1443926	1	112,461,902	A	G	0.68	0.1918	0.0286	1.21	2.0E-11	38.1	38799	*KCND3*	intron	0.099	0.765
rs4839184	1	112,460,221	C	G	0.32	-0.1909	0.0286	0.83	2.6E-11	37.6	39307	*KCND3*	intron	0.082	0.846
rs2010749	1	112,470,581	T	C	0.63	0.1835	0.0277	1.20	3.7E-11	42.6	39229	*KCND3*	intron	0.065	0.911
rs12119724	1	112,468,814	T	C	0.29	0.1902	0.0292	1.21	7.3E-11	5.5	38772	*KCND3*	intron	0.029	0.940
rs17029069	1	112,464,376	T	C	0.30	0.1875	0.0291	1.21	1.1E-10	1.6	38786	*KCND3*	intron	0.019	0.960
rs1443927	1	112,471,029	C	G	0.69	0.1864	0.0292	1.20	1.9E-10	42.6	38818	*KCND3*	intron	0.014	0.973
rs6682872	1	112,462,984	A	G	0.60	0.1649	0.027	1.18	9.6E-10	39.2	38918	*KCND3*	intron	0.003	0.977
rs2813865	1	112,437,956	A	G	0.25	-0.1964	0.032	0.82	8.6E-10	66.0	39360	*KCND3*	intron	0.003	0.979
rs583731	1	112,421,854	T	C	0.09	-0.3726	0.0563	0.69	3.6E-11	43.9	38199	*KCND3*	intron	0.002	0.981
rs4838927	1	112,463,617	T	C	0.60	0.1623	0.027	1.18	1.7E-09	42.1	39230	*KCND3*	intron	0.002	0.983
rs4838926	1	112,463,323	C	G	0.40	-0.1618	0.027	0.85	2.0E-09	41.1	39230	*KCND3*	intron	0.002	0.985
rs7539683	1	112,439,770	T	G	0.27	0.1806	0.0301	1.20	2.0E-09	0.0	38700	*KCND3*	intron	0.001	0.987
rs72694603	1	112,458,893	T	C	0.32	-0.1805	0.0302	0.83	2.3E-09	30.2	35129	*KCND3*	intron	0.001	0.988
rs72692602	1	112,458,833	T	C	0.68	0.1801	0.0302	1.20	2.5E-09	29.8	35129	*KCND3*	intron	0.001	0.989
rs72692597	1	112,455,442	T	G	0.32	-0.1796	0.0302	0.84	2.9E-09	31.0	35129	*KCND3*	intron	0.001	0.990

A1: effect allele; AF1: allele frequency of A1; Effect: association effect (log OR) of A1; SE: standard error of the effect; OR: odds ratio; P: association p-value; I^2: percentage of total variation across studies that is due to heterogeneity; N: sample size; PP: posterior probability of being causal.

Supplementary Table 3: Association results of rs1545300 stratified by ERP subtypes in the LifeLines cohort

Subtype	OR	lower CI	upper CI	P	N cases	N controls
not stratified	0.78	0.71	0.85	1.18E-07	1253	11463
ST segment ascending	0.76	0.67	0.87	5.50E-05	622	11463
ST segment horizontal/descending	0.79	0.69	0.90	2.66E-04	630	11463
ERP localization inferior	0.81	0.71	0.93	2.49E-03	560	11463
ERP localization lateral	0.70	0.56	0.87	1.18E-03	229	11463
ERP localization inferior and lateral	0.77	0.67	0.90	7.05E-04	464	11463

OR: odds ratio related to minor T allele; CI: 95% confidence interval of the OR; P: association p-value; N cases, N controls: number of cases and controls, respectively.

Supplementary Table 4: GTEx cis eQTL lookup results of rs1545300 across 48 tissues

The GTEx variant id 1_112464004_C_T_b37 corresponds to rs1545300, and the transcript ENSG00000171385.5 to *KCND3*. The effect direction of the eQTL slope corresponds to the T allele of rs1545300.

tissue	transcript	tss_distance	N	MAC	P	slope	slope_se	FDR
Artery_Tibial	ENSG00000171385.5	-67773	441	219	2.98E-06	-0.19	0.04	0.01
Heart_Left_Ventricle	ENSG00000171385.5	-67773	303	154	2.92E-04	-0.12	0.03	0.19
Minor_Salivary_Gland	ENSG00000134255.9	781156	97	49	3.39E-04	-0.34	0.09	0.19
Heart_Atrial_Appendage	ENSG00000171385.5	-67773	297	147	1.58E-03	-0.13	0.04	0.49
Skin_Not_Sun_Exposed_Suprapubic	ENSG00000155367.11	-794095	387	200	1.89E-03	0.10	0.03	0.49
Adipose_Visceral_Omentum	ENSG00000231346.1	312659	355	172	1.91E-03	-0.15	0.05	0.49
Brain_Amygdala	ENSG00000121933.13	357420	100	47	2.03E-03	0.23	0.07	0.49
Artery_Aorta	ENSG00000231346.1	312659	299	152	2.94E-03	0.15	0.05	0.62
Esophagus_Muscularis	ENSG00000064886.9	720611	370	188	4.12E-03	0.13	0.05	0.71
Brain_Anterior_cingulate_cortex_BA24	ENSG00000155367.11	-794095	121	65	4.60E-03	0.22	0.07	0.71
Whole_Blood	ENSG00000155363.14	-751759	407	195	4.93E-03	-0.06	0.02	0.71
Heart_Left_Ventricle	ENSG00000143110.7	447590	303	154	6.14E-03	-0.15	0.05	0.71
Esophagus_Muscularis	ENSG00000116473.10	379164	370	188	6.60E-03	-0.09	0.03	0.71
Lung	ENSG00000155366.12	-786052	427	222	6.67E-03	-0.07	0.02	0.71
Artery_Aorta	ENSG00000064886.9	720611	299	152	6.72E-03	0.17	0.06	0.71
Brain_Caudate_basal_ganglia	ENSG00000134245.13	-545159	160	88	7.52E-03	0.14	0.05	0.71
Colon_Sigmoid	ENSG00000121933.13	357420	233	107	7.62E-03	-0.18	0.07	0.71
Minor_Salivary_Gland	ENSG00000173947.9	575094	97	49	8.76E-03	0.22	0.08	0.71
Ovary	ENSG00000260948.1	488314	133	71	8.89E-03	-0.23	0.09	0.71
Whole_Blood	ENSG00000116489.8	-698415	407	195	9.00E-03	-0.04	0.01	0.71
Brain_Caudate_basal_ganglia	ENSG00000273010.1	958080	160	88	9.92E-03	0.34	0.13	0.71
Ovary	ENSG00000085465.11	493605	133	71	1.01E-02	-0.29	0.11	0.71
Colon_Sigmoid	ENSG00000155363.14	-751759	233	107	1.02E-02	-0.12	0.05	0.71
Brain_Anterior_cingulate_cortex_BA24	ENSG00000134255.9	781156	121	65	1.02E-02	0.16	0.06	0.71
Esophagus_Gastroesophageal_Junction	ENSG00000227811.2	181541	244	110	1.09E-02	-0.24	0.09	0.71
Brain_Spinal_cord_cervical_c-1	ENSG00000232811.1	977915	91	49	1.21E-02	0.48	0.18	0.71
Colon_Sigmoid	ENSG00000007341.14	-699443	233	107	1.27E-02	-0.22	0.09	0.71
Testis	ENSG00000155367.11	-794095	259	128	1.32E-02	0.07	0.03	0.71
Small_Intestine_Terminal_Ileum	ENSG00000197852.8	168128	137	54	1.46E-02	0.22	0.09	0.71
Brain_Spinal_cord_cervical_c-1	ENSG00000260948.1	488314	91	49	1.47E-02	-0.36	0.14	0.71
Breast_Mammary_Tissue	ENSG00000064886.9	720611	290	148	1.52E-02	-0.17	0.07	0.71
Artery_Aorta	ENSG00000171385.5	-67773	299	152	1.61E-02	-0.14	0.06	0.71
Brain_Cerebellum	ENSG00000143079.10	-474799	173	96	1.62E-02	0.18	0.07	0.71
Brain_Amygdala	ENSG00000116459.6	472490	100	47	1.63E-02	-0.15	0.06	0.71
Adipose_Subcutaneous	ENSG00000116459.6	472490	442	214	1.64E-02	0.08	0.03	0.71
Adrenal_Gland	ENSG00000273483.1	-597059	190	96	1.64E-02	0.19	0.08	0.71
Pituitary	ENSG00000064886.9	720611	183	95	1.64E-02	0.17	0.07	0.71
Brain_Anterior_cingulate_cortex_BA24	ENSG00000121933.13	357420	121	65	1.72E-02	0.18	0.07	0.71
Breast_Mammary_Tissue	ENSG00000064703.7	166137	290	148	1.73E-02	0.14	0.06	0.71
Artery_Coronary	ENSG00000143110.7	447590	173	81	1.74E-02	-0.11	0.05	0.71

Minor_Salivary_Gland	ENSG00000156171.10	781166	97	49	1.74E-02	-0.27	0.11	0.71
Artery_Aorta	ENSG00000155363.14	-751759	299	152	1.76E-02	-0.09	0.04	0.71
Liver	ENSG00000143110.7	447590	175	88	1.80E-02	-0.16	0.07	0.71
Prostate	ENSG00000155363.14	-751759	152	75	1.87E-02	-0.13	0.06	0.71
Esophagus_Gastroesophageal_Junction	ENSG00000155366.12	-786052	244	110	1.89E-02	-0.12	0.05	0.71
Brain_Spinal_cord_cervical_c-1	ENSG00000236040.1	671128	91	49	2.09E-02	0.41	0.17	0.72
Minor_Salivary_Gland	ENSG00000260948.1	488314	97	49	2.09E-02	0.24	0.10	0.72
Brain_Hippocampus	ENSG00000184599.9	-799037	123	63	2.10E-02	-0.28	0.12	0.72
Prostate	ENSG00000143079.10	-474799	152	75	2.10E-02	-0.15	0.06	0.72
Brain_Caudate_basal_ganglia	ENSG00000184599.9	-799037	160	88	2.19E-02	-0.28	0.12	0.74
Minor_Salivary_Gland	ENSG00000232811.1	977915	97	49	2.26E-02	0.34	0.15	0.75
Colon_Transverse	ENSG00000134245.13	-545159	274	119	2.34E-02	-0.11	0.05	0.76
Stomach	ENSG00000007341.14	-699443	262	107	2.40E-02	-0.19	0.08	0.76
Minor_Salivary_Gland	ENSG00000261654.1	979029	97	49	2.49E-02	0.33	0.14	0.77
Whole_Blood	ENSG00000261654.1	979029	407	195	2.53E-02	0.13	0.06	0.77
Small_Intestine_Terminal_Ileum	ENSG00000155367.11	-794095	137	54	2.54E-02	-0.20	0.09	0.77
Pituitary	ENSG00000231346.1	312659	183	95	2.73E-02	-0.19	0.09	0.78
Testis	ENSG00000261595.1	-794290	259	128	2.73E-02	0.08	0.04	0.78
Adrenal_Gland	ENSG00000143110.7	447590	190	96	2.74E-02	-0.14	0.06	0.78
Testis	ENSG00000197852.8	168128	259	128	2.87E-02	-0.17	0.08	0.78
Brain_Cerebellar_Hemisphere	ENSG00000243960.1	482557	136	74	2.88E-02	-0.25	0.11	0.78
Skin_Sun_Exposed_Lower_leg	ENSG00000261654.1	979029	473	233	2.97E-02	0.15	0.07	0.78
Breast_Mammary_Tissue	ENSG00000162777.12	716847	290	148	2.97E-02	0.09	0.04	0.78
Esophagus_Mucosa	ENSG00000243960.1	482557	407	208	2.99E-02	0.18	0.08	0.78
Pituitary	ENSG00000007341.14	-699443	183	95	3.03E-02	-0.25	0.11	0.78
Heart_Atrial_Appendage	ENSG00000155363.14	-751759	297	147	3.10E-02	-0.07	0.03	0.78
Nerve_Tibial	ENSG00000155367.11	-794095	414	202	3.10E-02	0.08	0.04	0.78
Spleen	ENSG00000231346.1	312659	162	55	3.14E-02	-0.28	0.13	0.78
Small_Intestine_Terminal_Ileum	ENSG00000215867.4	271838	137	54	3.31E-02	-0.34	0.16	0.81
Esophagus_Mucosa	ENSG00000085465.11	493605	407	208	3.34E-02	0.08	0.04	0.81
Pituitary	ENSG00000156171.10	781166	183	95	3.42E-02	-0.20	0.09	0.81
Brain_Nucleus_accumbens_basal_ganglia	ENSG00000134245.13	-545159	147	77	3.49E-02	-0.14	0.07	0.81
Brain_Spinal_cord_cervical_c-1	ENSG00000231437.3	-68388	91	49	3.52E-02	0.21	0.10	0.81
Brain_Caudate_basal_ganglia	ENSG00000121933.13	357420	160	88	3.64E-02	0.15	0.07	0.81
Brain_Substantia_nigra	ENSG00000116459.6	472490	88	49	3.67E-02	0.15	0.07	0.81
Brain_Frontal_Cortex_BA9	ENSG00000134245.13	-545159	129	73	3.67E-02	-0.22	0.10	0.81
Liver	ENSG00000116489.8	-698415	175	88	3.76E-02	-0.10	0.05	0.81
Esophagus_Mucosa	ENSG00000184599.9	-799037	407	208	3.79E-02	-0.15	0.07	0.81
Minor_Salivary_Gland	ENSG00000231346.1	312659	97	49	3.83E-02	-0.25	0.12	0.81
Adrenal_Gland	ENSG00000064703.7	166137	190	96	3.84E-02	0.15	0.07	0.81
Esophagus_Mucosa	ENSG00000231437.3	-68388	407	208	4.08E-02	0.11	0.06	0.84
Cells_EBV-transformed_lymphocytes	ENSG00000007341.14	-699443	130	68	4.10E-02	-0.24	0.11	0.84
Small_Intestine_Terminal_Ileum	ENSG00000273010.1	958080	137	54	4.32E-02	0.29	0.14	0.86
Artery_Coronary	ENSG00000116455.9	472519	173	81	4.33E-02	-0.09	0.04	0.86
Adipose_Subcutaneous	ENSG00000116473.10	379164	442	214	4.35E-02	-0.07	0.03	0.86
Adrenal_Gland	ENSG00000155366.12	-786052	190	96	4.44E-02	0.12	0.06	0.86
Artery_Tibial	ENSG00000007341.14	-699443	441	219	4.56E-02	-0.12	0.06	0.86

Brain_Hippocampus	ENSG00000155367.11	-794095	123	63	4.66E-02	-0.15	0.08	0.86
Heart_Atrial_Appendage	ENSG00000116489.8	-698415	297	147	4.72E-02	0.06	0.03	0.86
Brain_Hypothalamus	ENSG00000085465.11	493605	121	61	4.78E-02	0.14	0.07	0.86
Testis	ENSG00000064703.7	166137	259	128	4.81E-02	0.05	0.03	0.86
Cells_Transformed_fibroblasts	ENSG00000243960.1	482557	343	175	4.85E-02	0.14	0.07	0.86
Thyroid	ENSG00000007341.14	-699443	446	224	4.87E-02	-0.11	0.06	0.86
Cells_EBV-transformed_lymphocytes	ENSG00000197852.8	168128	130	68	4.97E-02	0.20	0.10	0.86
Nerve_Tibial	ENSG00000232811.1	977915	414	202	5.07E-02	-0.15	0.07	0.86
Cells_EBV-transformed_lymphocytes	ENSG00000155363.14	-751759	130	68	5.17E-02	0.16	0.08	0.86
Brain_Cerebellum	ENSG00000231437.3	-68388	173	96	5.18E-02	0.15	0.08	0.86
Skin_Sun_Exposed_Lower_leg	ENSG00000116489.8	-698415	473	233	5.29E-02	-0.05	0.02	0.86
Colon_Transverse	ENSG00000156171.10	781166	274	119	5.33E-02	-0.10	0.05	0.86
Nerve_Tibial	ENSG00000007341.14	-699443	414	202	5.36E-02	-0.12	0.06	0.86
Cells_Transformed_fibroblasts	ENSG00000261654.1	979029	343	175	5.50E-02	-0.10	0.05	0.86
Esophagus_Gastroesophageal_Junction	ENSG00000064703.7	166137	244	110	5.62E-02	0.11	0.06	0.86
Brain_Hippocampus	ENSG00000233337.1	483868	123	63	5.65E-02	-0.22	0.12	0.86
Esophagus_Muscularis	ENSG00000007341.14	-699443	370	188	5.67E-02	-0.13	0.07	0.86
Breast_Mammary_Tissue	ENSG00000121933.13	357420	290	148	5.71E-02	-0.10	0.05	0.86
Stomach	ENSG00000121931.11	968421	262	107	5.75E-02	-0.15	0.08	0.86
Prostate	ENSG00000173947.9	575094	152	75	5.75E-02	-0.12	0.06	0.86
Pancreas	ENSG00000273483.1	-597059	248	108	5.77E-02	-0.18	0.09	0.86
Muscle_Skeletal	ENSG00000273010.1	958080	564	276	5.82E-02	0.10	0.05	0.86
Colon_Sigmoid	ENSG00000064703.7	166137	233	107	5.84E-02	0.10	0.05	0.86
Brain_Nucleus_accumbens_basal_ganglia	ENSG00000116489.8	-698415	147	77	5.90E-02	-0.13	0.07	0.86
Stomach	ENSG00000143110.7	447590	262	107	5.92E-02	-0.10	0.05	0.86
Cells_Transformed_fibroblasts	ENSG00000155366.12	-786052	343	175	5.95E-02	-0.04	0.02	0.86
Brain_Hypothalamus	ENSG00000121933.13	357420	121	61	5.99E-02	0.11	0.06	0.86
Breast_Mammary_Tissue	ENSG00000233337.1	483868	290	148	6.05E-02	0.15	0.08	0.86
Brain_Nucleus_accumbens_basal_ganglia	ENSG00000224167.1	-955558	147	77	6.09E-02	0.25	0.13	0.86
Colon_Sigmoid	ENSG00000231246.1	-439146	233	107	6.11E-02	0.18	0.10	0.86
Spleen	ENSG00000232811.1	977915	162	55	6.19E-02	0.26	0.14	0.86
Liver	ENSG00000116455.9	472519	175	88	6.34E-02	-0.10	0.06	0.86
Adipose_Visceral_Omentum	ENSG00000155363.14	-751759	355	172	6.38E-02	-0.06	0.03	0.86
Cells_Transformed_fibroblasts	ENSG00000007341.14	-699443	343	175	6.42E-02	-0.14	0.07	0.86
Brain_Cerebellum	ENSG00000116455.9	472519	173	96	6.48E-02	0.13	0.07	0.86
Colon_Transverse	ENSG00000215866.3	-929261	274	119	6.59E-02	0.15	0.08	0.86
Breast_Mammary_Tissue	ENSG00000243960.1	482557	290	148	6.74E-02	0.17	0.09	0.86
Skin_Sun_Exposed_Lower_leg	ENSG00000171385.5	-67773	473	233	6.76E-02	0.07	0.04	0.86
Esophagus_Gastroesophageal_Junction	ENSG00000155363.14	-751759	244	110	6.79E-02	-0.09	0.05	0.86
Pancreas	ENSG00000143110.7	447590	248	108	6.79E-02	-0.11	0.06	0.86
Heart_Atrial_Appendage	ENSG00000197852.8	168128	297	147	6.82E-02	0.08	0.04	0.86
Cells_EBV-transformed_lymphocytes	ENSG00000116489.8	-698415	130	68	6.90E-02	-0.10	0.05	0.86
Brain_Nucleus_accumbens_basal_ganglia	ENSG00000227811.2	181541	147	77	6.92E-02	-0.20	0.11	0.86
Brain_Hippocampus	ENSG00000243960.1	482557	123	63	6.93E-02	0.29	0.16	0.86
Skin_Sun_Exposed_Lower_leg	ENSG00000064703.7	166137	473	233	7.03E-02	0.08	0.04	0.86
Brain_Caudate_basal_ganglia	ENSG00000231437.3	-68388	160	88	7.05E-02	0.15	0.08	0.86

Colon_Sigmoid	ENSG00000116455.9	472519	233	107	7.07E-02	-0.08	0.05	0.86
Brain_Amygdala	ENSG00000184599.9	-799037	100	47	7.09E-02	0.35	0.19	0.86
Pancreas	ENSG00000233337.1	483868	248	108	7.09E-02	0.16	0.09	0.86
Pancreas	ENSG00000173947.9	575094	248	108	7.10E-02	0.14	0.08	0.86
Skin_Sun_Exposed_Lower_leg	ENSG00000143079.10	-474799	473	233	7.12E-02	0.04	0.02	0.86
Spleen	ENSG00000238975.1	-731206	162	55	7.25E-02	-0.23	0.12	0.86
Whole_Blood	ENSG00000156171.10	781166	407	195	7.27E-02	-0.06	0.03	0.86
Small_Intestine_Terminal_Ileum	ENSG00000116473.10	379164	137	54	7.29E-02	-0.15	0.08	0.86
Uterus	ENSG00000273010.1	958080	111	63	7.31E-02	0.26	0.14	0.86
Prostate	ENSG00000232811.1	977915	152	75	7.45E-02	-0.22	0.12	0.86
Brain_Putamen_basal_ganglia	ENSG00000231346.1	312659	124	71	7.48E-02	-0.25	0.14	0.86
Vagina	ENSG00000116459.6	472490	115	59	7.61E-02	0.06	0.04	0.86
Testis	ENSG00000116455.9	472519	259	128	7.62E-02	0.10	0.06	0.86
Pancreas	ENSG00000116455.9	472519	248	108	7.72E-02	0.12	0.07	0.86
Adrenal_Gland	ENSG00000143079.10	-474799	190	96	7.75E-02	0.13	0.07	0.86
Cells_EBV-transformed_lymphocytes	ENSG00000261654.1	979029	130	68	7.75E-02	0.18	0.10	0.86
Prostate	ENSG00000261654.1	979029	152	75	7.82E-02	-0.21	0.12	0.86
Muscle_Skeletal	ENSG00000116455.9	472519	564	276	7.83E-02	0.05	0.03	0.86
Heart_Left_Ventricle	ENSG00000227811.2	181541	303	154	7.85E-02	0.12	0.07	0.86
Skin_Not_Sun_Exposed_Suprapubic	ENSG00000155366.12	-786052	387	200	7.92E-02	-0.06	0.03	0.86
Cells_Transformed_fibroblasts	ENSG00000184599.9	-799037	343	175	7.99E-02	-0.13	0.07	0.86
Cells_Transformed_fibroblasts	ENSG00000116473.10	379164	343	175	8.13E-02	-0.05	0.03	0.86
Thyroid	ENSG00000273010.1	958080	446	224	8.15E-02	-0.13	0.07	0.86
Colon_Sigmoid	ENSG00000121931.11	968421	233	107	8.17E-02	0.14	0.08	0.86
Adipose_Visceral_Omentum	ENSG00000116473.10	379164	355	172	8.24E-02	-0.05	0.03	0.86
Pancreas	ENSG00000134245.13	-545159	248	108	8.38E-02	0.12	0.07	0.86
Ovary	ENSG00000116455.9	472519	133	71	8.41E-02	0.11	0.06	0.86
Thyroid	ENSG00000116455.9	472519	446	224	8.45E-02	0.05	0.03	0.86
Breast_Mammary_Tissue	ENSG00000155366.12	-786052	290	148	8.49E-02	-0.06	0.03	0.86
Thyroid	ENSG00000116473.10	379164	446	224	8.49E-02	-0.05	0.03	0.86
Minor_Salivary_Gland	ENSG00000007341.14	-699443	97	49	8.50E-02	-0.28	0.16	0.86
Lung	ENSG00000238975.1	-731206	427	222	8.65E-02	0.09	0.05	0.86
Spleen	ENSG00000116459.6	472490	162	55	8.73E-02	0.08	0.05	0.86
Pituitary	ENSG00000171385.5	-67773	183	95	8.78E-02	0.15	0.09	0.86
Brain_Cerebellar_Hemisphere	ENSG00000116459.6	472490	136	74	8.85E-02	-0.08	0.05	0.86
Skin_Sun_Exposed_Lower_leg	ENSG00000064886.9	720611	473	233	8.88E-02	0.07	0.04	0.86
Brain_Amygdala	ENSG00000134255.9	781156	100	47	8.93E-02	0.19	0.11	0.86
Esophagus_Muscularis	ENSG00000225075.1	-775239	370	188	8.98E-02	0.13	0.08	0.86
Brain_Cerebellar_Hemisphere	ENSG00000227811.2	181541	136	74	8.99E-02	0.23	0.13	0.86
Esophagus_Gastroesophageal_Junction	ENSG00000116489.8	-698415	244	110	9.09E-02	-0.08	0.05	0.86
Brain_Substantia_nigra	ENSG00000155366.12	-786052	88	49	9.18E-02	0.10	0.06	0.86
Colon_Transverse	ENSG00000134255.9	781156	274	119	9.20E-02	-0.06	0.04	0.86
Pituitary	ENSG00000143110.7	447590	183	95	9.21E-02	0.11	0.07	0.86
Pituitary	ENSG00000143079.10	-474799	183	95	9.23E-02	0.12	0.07	0.86
Liver	ENSG00000116473.10	379164	175	88	9.26E-02	-0.13	0.08	0.86
Brain_Substantia_nigra	ENSG00000162777.12	716847	88	49	9.27E-02	0.16	0.09	0.86
Testis	ENSG00000155363.14	-751759	259	128	9.43E-02	0.04	0.03	0.86

Brain_Caudate_basal_ganglia	ENSG00000171385.5	-67773	160	88	9.58E-02	0.13	0.08	0.86
Uterus	ENSG00000260948.1	488314	111	63	9.59E-02	-0.18	0.11	0.86
Adipose_Visceral_Omentum	ENSG00000155367.11	-794095	355	172	9.59E-02	-0.08	0.05	0.86
Esophagus_Mucosa	ENSG00000134245.13	-545159	407	208	9.63E-02	-0.06	0.04	0.86
Brain_Substantia_nigra	ENSG00000243960.1	482557	88	49	9.65E-02	0.25	0.15	0.86
Adipose_Visceral_Omentum	ENSG00000227811.2	181541	355	172	9.71E-02	-0.13	0.08	0.86
Brain_Putamen_basal_ganglia	ENSG00000231437.3	-68388	124	71	9.77E-02	0.15	0.09	0.86
Ovary	ENSG00000134245.13	-545159	133	71	9.79E-02	0.14	0.08	0.86
Brain_Hypothalamus	ENSG00000231346.1	312659	121	61	9.94E-02	0.19	0.12	0.86
Testis	ENSG00000231246.1	-439146	259	128	9.95E-02	0.11	0.07	0.86
Brain_Cortex	ENSG00000116459.6	472490	158	79	9.99E-02	-0.12	0.07	0.86
Spleen	ENSG00000116473.10	379164	162	55	1.00E-01	-0.13	0.08	0.86
Heart_Left_Ventricle	ENSG00000085465.11	493605	303	154	1.00E-01	-0.08	0.05	0.86
Stomach	ENSG00000238975.1	-731206	262	107	1.01E-01	0.15	0.09	0.86
Esophagus_Mucosa	ENSG00000225075.1	-775239	407	208	1.01E-01	-0.12	0.07	0.86
Brain_Amygdala	ENSG00000231437.3	-68388	100	47	1.02E-01	0.23	0.14	0.86
Brain_Cortex	ENSG00000197852.8	168128	158	79	1.02E-01	-0.09	0.05	0.86
Brain_Frontal_Cortex_BA9	ENSG00000233337.1	483868	129	73	1.03E-01	0.23	0.14	0.86
Thyroid	ENSG00000215866.3	-929261	446	224	1.03E-01	0.11	0.07	0.86
Nerve_Tibial	ENSG00000121931.11	968421	414	202	1.04E-01	-0.06	0.04	0.86
Artery_Coronary	ENSG00000143079.10	-474799	173	81	1.04E-01	0.12	0.07	0.86
Esophagus_Mucosa	ENSG00000143079.10	-474799	407	208	1.05E-01	0.04	0.03	0.86
Small_Intestine_Terminal_Ileum	ENSG00000173947.9	575094	137	54	1.07E-01	-0.13	0.08	0.86
Pituitary	ENSG00000162777.12	716847	183	95	1.07E-01	0.13	0.08	0.86
Pituitary	ENSG00000116455.9	472519	183	95	1.07E-01	-0.10	0.06	0.86
Brain_Spinal_cord_cervical_c-1	ENSG00000171385.5	-67773	91	49	1.07E-01	0.17	0.11	0.86
Testis	ENSG00000116459.6	472490	259	128	1.07E-01	0.08	0.05	0.86
Skin_Sun_Exposed_Lower_leg	ENSG00000273010.1	958080	473	233	1.08E-01	-0.12	0.07	0.86
Small_Intestine_Terminal_Ileum	ENSG00000156171.10	781166	137	54	1.08E-01	0.10	0.06	0.86
Minor_Salivary_Gland	ENSG00000064886.9	720611	97	49	1.08E-01	-0.11	0.07	0.86
Uterus	ENSG00000064703.7	166137	111	63	1.09E-01	0.15	0.09	0.86
Heart_Atrial_Appendage	ENSG00000233337.1	483868	297	147	1.09E-01	-0.13	0.08	0.86
Artery_Tibial	ENSG00000232811.1	977915	441	219	1.10E-01	-0.11	0.07	0.86
Lung	ENSG00000232811.1	977915	427	222	1.10E-01	0.11	0.07	0.86
Brain_Nucleus_accumbens_basal_ganglia	ENSG00000273483.1	-597059	147	77	1.10E-01	0.12	0.08	0.86
Minor_Salivary_Gland	ENSG00000273483.1	-597059	97	49	1.11E-01	0.22	0.14	0.86
Esophagus_Muscularis	ENSG00000155363.14	-751759	370	188	1.12E-01	-0.05	0.03	0.86
Cells_Transformed_fibroblasts	ENSG00000225075.1	-775239	343	175	1.12E-01	-0.13	0.08	0.86
Pancreas	ENSG00000121933.13	357420	248	108	1.12E-01	-0.08	0.05	0.86
Heart_Atrial_Appendage	ENSG00000243960.1	482557	297	147	1.14E-01	0.14	0.09	0.87
Brain_Anterior_cingulate_cortex_BA24	ENSG00000085465.11	493605	121	65	1.14E-01	0.11	0.07	0.87
Pituitary	ENSG00000197852.8	168128	183	95	1.15E-01	-0.17	0.11	0.87
Pituitary	ENSG00000155367.11	-794095	183	95	1.16E-01	0.15	0.10	0.88
Brain_Cerebellar_Hemisphere	ENSG00000155367.11	-794095	136	74	1.17E-01	-0.13	0.08	0.88
Ovary	ENSG00000232811.1	977915	133	71	1.17E-01	-0.20	0.12	0.88
Thyroid	ENSG00000064703.7	166137	446	224	1.18E-01	-0.04	0.03	0.88
Prostate	ENSG00000273010.1	958080	152	75	1.19E-01	-0.19	0.12	0.88

Brain_Cerebellum	ENSG00000215866.3	-929261	173	96	1.21E-01	0.18	0.11	0.88
Artery_Aorta	ENSG00000116489.8	-698415	299	152	1.22E-01	0.05	0.03	0.88
Brain_Amygdala	ENSG00000231346.1	312659	100	47	1.22E-01	0.22	0.14	0.88
Brain_Spinal_cord_cervical_c-1	ENSG00000134255.9	781156	91	49	1.22E-01	0.12	0.08	0.88
Spleen	ENSG00000121931.11	968421	162	55	1.25E-01	-0.17	0.11	0.88
Brain_Hippocampus	ENSG00000121933.13	357420	123	63	1.25E-01	0.08	0.05	0.88
Brain_Cerebellum	ENSG00000215867.4	271838	173	96	1.25E-01	-0.18	0.12	0.88
Esophagus_Muscularis	ENSG00000171385.5	-67773	370	188	1.25E-01	-0.04	0.03	0.88
Spleen	ENSG00000243960.1	482557	162	55	1.25E-01	-0.23	0.15	0.88
Brain_Putamen_basal_ganglia	ENSG00000232811.1	977915	124	71	1.26E-01	0.21	0.14	0.88
Testis	ENSG00000134245.13	-545159	259	128	1.27E-01	0.07	0.04	0.88
Nerve_Tibial	ENSG00000143079.10	-474799	414	202	1.27E-01	-0.05	0.03	0.88
Thyroid	ENSG00000064886.9	720611	446	224	1.28E-01	0.08	0.05	0.88
Brain_Substantia_nigra	ENSG00000227811.2	181541	88	49	1.30E-01	0.28	0.18	0.88
Brain_Hippocampus	ENSG00000173947.9	575094	123	63	1.30E-01	0.10	0.07	0.88
Breast_Mammary_Tissue	ENSG00000227811.2	181541	290	148	1.30E-01	0.13	0.09	0.88
Esophagus_Mucosa	ENSG00000156171.10	781166	407	208	1.30E-01	-0.08	0.06	0.88
Nerve_Tibial	ENSG00000121933.13	357420	414	202	1.32E-01	-0.06	0.04	0.88
Brain_Hypothalamus	ENSG00000231437.3	-68388	121	61	1.32E-01	0.15	0.10	0.88
Ovary	ENSG00000171385.5	-67773	133	71	1.33E-01	-0.14	0.09	0.88
Lung	ENSG00000121931.11	968421	427	222	1.33E-01	-0.04	0.03	0.88
Adrenal_Gland	ENSG00000134245.13	-545159	190	96	1.33E-01	0.11	0.07	0.88
Artery_Aorta	ENSG00000143079.10	-474799	299	152	1.33E-01	0.07	0.04	0.88
Esophagus_Gastroesophageal_Junction	ENSG00000261654.1	979029	244	110	1.34E-01	-0.14	0.09	0.88
Colon_Transverse	ENSG00000007341.14	-699443	274	119	1.34E-01	-0.13	0.09	0.88
Artery_Tibial	ENSG00000155367.11	-794095	441	219	1.34E-01	0.06	0.04	0.88
Brain_Cortex	ENSG00000231437.3	-68388	158	79	1.34E-01	0.18	0.12	0.88
Thyroid	ENSG00000238975.1	-731206	446	224	1.35E-01	0.09	0.06	0.88
Vagina	ENSG00000116455.9	472519	115	59	1.36E-01	0.11	0.07	0.88
Prostate	ENSG00000116473.10	379164	152	75	1.37E-01	-0.07	0.05	0.88
Artery_Coronary	ENSG00000156171.10	781166	173	81	1.38E-01	-0.10	0.07	0.88
Brain_Substantia_nigra	ENSG00000173947.9	575094	88	49	1.38E-01	0.18	0.12	0.88
Adipose_Subcutaneous	ENSG00000171385.5	-67773	442	214	1.39E-01	-0.06	0.04	0.88
Pancreas	ENSG00000143079.10	-474799	248	108	1.41E-01	0.09	0.06	0.88
Brain_Substantia_nigra	ENSG00000134255.9	781156	88	49	1.41E-01	0.10	0.07	0.88
Stomach	ENSG00000229283.1	603455	262	107	1.42E-01	0.10	0.07	0.88
Adipose_Subcutaneous	ENSG00000162777.12	716847	442	214	1.42E-01	0.05	0.03	0.88
Testis	ENSG00000237556.1	12046	259	128	1.42E-01	0.07	0.05	0.88
Brain_Substantia_nigra	ENSG00000116473.10	379164	88	49	1.43E-01	0.09	0.06	0.88
Brain_Frontal_Cortex_BA9	ENSG00000116473.10	379164	129	73	1.43E-01	-0.08	0.05	0.88
Nerve_Tibial	ENSG00000261654.1	979029	414	202	1.44E-01	-0.09	0.06	0.88
Testis	ENSG00000156171.10	781166	259	128	1.44E-01	-0.06	0.04	0.88
Skin_Sun_Exposed_Lower_leg	ENSG00000227811.2	181541	473	233	1.44E-01	-0.11	0.08	0.88
Brain_Caudate_basal_ganglia	ENSG00000143079.10	-474799	160	88	1.45E-01	0.11	0.07	0.88
Heart_Atrial_Appendage	ENSG00000227811.2	181541	297	147	1.45E-01	0.09	0.06	0.88
Esophagus_Gastroesophageal_Junction	ENSG00000231346.1	312659	244	110	1.46E-01	0.08	0.06	0.88
Small_Intestine_Terminal_Ileum	ENSG00000116459.6	472490	137	54	1.46E-01	-0.06	0.04	0.88

Vagina	ENSG00000231437.3	-68388	115	59	1.47E-01	0.16	0.11	0.88
Brain_Cerebellar_Hemisphere	ENSG00000116489.8	-698415	136	74	1.47E-01	-0.14	0.10	0.88
Brain_Cortex	ENSG00000261654.1	979029	158	79	1.47E-01	0.16	0.11	0.88
Testis	ENSG00000273010.1	958080	259	128	1.48E-01	-0.09	0.06	0.88
Esophagus_Muscularis	ENSG00000116455.9	472519	370	188	1.49E-01	0.05	0.03	0.88
Brain_Substantia_nigra	ENSG00000184599.9	-799037	88	49	1.49E-01	0.14	0.09	0.88
Brain_Spinal_cord_cervical_c-1	ENSG00000064703.7	166137	91	49	1.50E-01	0.19	0.13	0.88
Nerve_Tibial	ENSG00000116489.8	-698415	414	202	1.50E-01	-0.05	0.03	0.88
Brain_Spinal_cord_cervical_c-1	ENSG00000134245.13	-545159	91	49	1.51E-01	-0.20	0.14	0.88
Brain_Anterior_cingulate_cortex_BA24	ENSG00000232811.1	977915	121	65	1.51E-01	-0.20	0.14	0.88
Cells_EBV-transformed_lymphocytes	ENSG00000273010.1	958080	130	68	1.51E-01	-0.20	0.14	0.88
Brain_Nucleus_accumbens_basal_ganglia	ENSG00000231346.1	312659	147	77	1.52E-01	0.18	0.13	0.88
Esophagus_Mucosa	ENSG00000007341.14	-699443	407	208	1.53E-01	-0.08	0.05	0.88
Lung	ENSG00000215867.4	271838	427	222	1.53E-01	-0.10	0.07	0.88
Brain_Frontal_Cortex_BA9	ENSG00000143110.7	447590	129	73	1.53E-01	0.11	0.08	0.88
Artery_Aorta	ENSG00000225075.1	-775239	299	152	1.55E-01	0.13	0.09	0.88
Brain_Caudate_basal_ganglia	ENSG00000231346.1	312659	160	88	1.56E-01	0.15	0.11	0.88
Uterus	ENSG00000227811.2	181541	111	63	1.56E-01	-0.20	0.14	0.88
Brain_Cortex	ENSG00000155367.11	-794095	158	79	1.58E-01	0.13	0.09	0.88
Heart_Atrial_Appendage	ENSG00000173947.9	575094	297	147	1.59E-01	-0.05	0.03	0.88
Brain_Hypothalamus	ENSG00000134255.9	781156	121	61	1.59E-01	-0.08	0.05	0.88
Cells_Transformed_fibroblasts	ENSG00000260948.1	488314	343	175	1.60E-01	0.05	0.04	0.88
Brain_Caudate_basal_ganglia	ENSG00000121931.11	968421	160	88	1.60E-01	0.13	0.09	0.88
Lung	ENSG00000085465.11	493605	427	222	1.61E-01	0.04	0.03	0.88
Liver	ENSG00000121931.11	968421	175	88	1.61E-01	0.11	0.07	0.88
Skin_Sun_Exposed_Lower_leg	ENSG00000273483.1	-597059	473	233	1.61E-01	-0.10	0.07	0.88
Liver	ENSG00000231437.3	-68388	175	88	1.61E-01	-0.14	0.10	0.88
Prostate	ENSG00000162777.12	716847	152	75	1.62E-01	-0.08	0.05	0.88
Ovary	ENSG00000064703.7	166137	133	71	1.62E-01	0.10	0.07	0.88
Artery_Tibial	ENSG00000156171.10	781166	441	219	1.63E-01	-0.05	0.04	0.88
Skin_Sun_Exposed_Lower_leg	ENSG00000238975.1	-731206	473	233	1.65E-01	-0.08	0.06	0.88
Minor_Salivary_Gland	ENSG00000233337.1	483868	97	49	1.65E-01	0.13	0.09	0.88
Esophagus_Gastroesophageal_Junction	ENSG00000143079.10	-474799	244	110	1.65E-01	-0.10	0.07	0.88
Cells_Transformed_fibroblasts	ENSG00000273010.1	958080	343	175	1.65E-01	-0.13	0.09	0.88
Artery_Tibial	ENSG00000227811.2	181541	441	219	1.66E-01	0.10	0.07	0.88
Small_Intestine_Terminal_Ileum	ENSG00000121933.13	357420	137	54	1.66E-01	0.15	0.11	0.88
Brain_Spinal_cord_cervical_c-1	ENSG00000173947.9	575094	91	49	1.66E-01	0.20	0.14	0.88
Small_Intestine_Terminal_Ileum	ENSG00000231437.3	-68388	137	54	1.67E-01	0.13	0.09	0.88
Breast_Mammary_Tissue	ENSG00000231346.1	312659	290	148	1.67E-01	0.10	0.07	0.88
Minor_Salivary_Gland	ENSG00000121931.11	968421	97	49	1.68E-01	0.15	0.11	0.88
Colon_Sigmoid	ENSG00000156171.10	781166	233	107	1.68E-01	-0.10	0.07	0.88
Spleen	ENSG00000273010.1	958080	162	55	1.68E-01	-0.22	0.16	0.88
Skin_Not_Sun_Exposed_Suprapubic	ENSG00000238975.1	-731206	387	200	1.70E-01	-0.08	0.06	0.88
Brain_Frontal_Cortex_BA9	ENSG00000064703.7	166137	129	73	1.70E-01	-0.15	0.11	0.88
Testis	ENSG00000243960.1	482557	259	128	1.70E-01	-0.13	0.09	0.88
Cells_EBV-transformed_lymphocytes	ENSG00000143110.7	447590	130	68	1.71E-01	0.13	0.09	0.88
Ovary	ENSG00000231437.3	-68388	133	71	1.72E-01	-0.15	0.11	0.88

Brain_Putamen_basal_ganglia	ENSG00000007341.14	-699443	124	71	1.72E-01	-0.19	0.14	0.88
Brain_Hypothalamus	ENSG00000162777.12	716847	121	61	1.72E-01	0.14	0.10	0.88
Cells_Transformed_fibroblasts	ENSG00000143079.10	-474799	343	175	1.73E-01	-0.05	0.03	0.88
Breast_Mammary_Tissue	ENSG00000007341.14	-699443	290	148	1.73E-01	-0.10	0.07	0.88
Artery_Tibial	ENSG00000231346.1	312659	441	219	1.73E-01	0.05	0.03	0.88
Liver	ENSG00000171385.5	-67773	175	88	1.74E-01	-0.10	0.07	0.88
Minor_Salivary_Gland	ENSG00000171385.5	-67773	97	49	1.74E-01	-0.13	0.09	0.88
Pancreas	ENSG00000197852.8	168128	248	108	1.74E-01	0.11	0.08	0.88
Cells_Transformed_fibroblasts	ENSG00000215866.3	-929261	343	175	1.75E-01	0.10	0.07	0.88
Brain_Frontal_Cortex_BA9	ENSG00000224167.1	-955558	129	73	1.75E-01	-0.19	0.14	0.88
Esophagus_Gastroesophageal_Junction	ENSG00000134245.13	-545159	244	110	1.76E-01	-0.09	0.06	0.88
Brain_Putamen_basal_ganglia	ENSG00000227811.2	181541	124	71	1.76E-01	-0.20	0.15	0.88
Brain_Hypothalamus	ENSG00000116455.9	472519	121	61	1.76E-01	-0.13	0.09	0.88
Brain_Hippocampus	ENSG00000064703.7	166137	123	63	1.77E-01	-0.15	0.11	0.88
Lung	ENSG00000156171.10	781166	427	222	1.78E-01	-0.06	0.04	0.88
Brain_Nucleus_accumbens_basal_ganglia	ENSG00000243960.1	482557	147	77	1.78E-01	0.19	0.14	0.88
Brain_Hippocampus	ENSG00000231437.3	-68388	123	63	1.78E-01	0.11	0.08	0.88
Prostate	ENSG00000143110.7	447590	152	75	1.78E-01	-0.11	0.08	0.88
Uterus	ENSG00000134255.9	781156	111	63	1.79E-01	-0.13	0.09	0.88
Brain_Putamen_basal_ganglia	ENSG00000162777.12	716847	124	71	1.80E-01	-0.15	0.11	0.88
Brain_Cortex	ENSG00000121933.13	357420	158	79	1.80E-01	0.12	0.09	0.88
Brain_Hypothalamus	ENSG00000243960.1	482557	121	61	1.80E-01	-0.18	0.14	0.88
Brain_Caudate_basal_ganglia	ENSG00000064886.9	720611	160	88	1.80E-01	-0.14	0.10	0.88
Esophagus_Muscularis	ENSG00000156171.10	781166	370	188	1.81E-01	-0.08	0.06	0.89
Lung	ENSG00000121933.13	357420	427	222	1.83E-01	0.04	0.03	0.89
Adipose_Subcutaneous	ENSG00000134245.13	-545159	442	214	1.84E-01	-0.06	0.05	0.89
Skin_Not_Sun_Exposed_Suprapubic	ENSG00000225075.1	-775239	387	200	1.85E-01	0.09	0.07	0.89
Adrenal_Gland	ENSG00000197852.8	168128	190	96	1.85E-01	0.12	0.09	0.89
Artery_Coronary	ENSG00000233337.1	483868	173	81	1.85E-01	-0.15	0.11	0.89
Adipose_Visceral_Omentum	ENSG00000171385.5	-67773	355	172	1.85E-01	0.08	0.06	0.89
Esophagus_Gastroesophageal_Junction	ENSG00000233337.1	483868	244	110	1.87E-01	-0.12	0.09	0.89
Brain_Nucleus_accumbens_basal_ganglia	ENSG00000121931.11	968421	147	77	1.88E-01	0.11	0.09	0.89
Artery_Coronary	ENSG00000261654.1	979029	173	81	1.89E-01	-0.15	0.11	0.89
Brain_Hippocampus	ENSG00000134245.13	-545159	123	63	1.89E-01	0.15	0.11	0.89
Brain_Amygdala	ENSG00000143079.10	-474799	100	47	1.89E-01	0.10	0.08	0.89
Small_Intestine_Terminal_Ileum	ENSG00000243960.1	482557	137	54	1.90E-01	-0.16	0.12	0.89
Brain_Substantia_nigra	ENSG00000232811.1	977915	88	49	1.91E-01	-0.22	0.17	0.89
Brain_Frontal_Cortex_BA9	ENSG00000116459.6	472490	129	73	1.92E-01	-0.11	0.08	0.89
Artery_Tibial	ENSG00000155363.14	-751759	441	219	1.93E-01	-0.04	0.03	0.89
Esophagus_Gastroesophageal_Junction	ENSG00000162777.12	716847	244	110	1.94E-01	-0.08	0.06	0.89
Adipose_Visceral_Omentum	ENSG00000231437.3	-68388	355	172	1.94E-01	0.07	0.05	0.89
Brain_Anterior_cingulate_cortex_BA24	ENSG00000233337.1	483868	121	65	1.94E-01	0.18	0.14	0.89
Muscle_Skeletal	ENSG00000231346.1	312659	564	276	1.94E-01	-0.08	0.06	0.89
Brain_Cerebellar_Hemisphere	ENSG00000260948.1	488314	136	74	1.94E-01	-0.12	0.09	0.89
Brain_Spinal_cord_cervical_c-1	ENSG00000116459.6	472490	91	49	1.95E-01	-0.12	0.09	0.89
Minor_Salivary_Gland	ENSG00000085465.11	493605	97	49	1.97E-01	0.12	0.09	0.89

Skin_Not_Sun_Exposed_Suprapubic	ENSG00000197852.8	168128	387	200	1.97E-01	0.05	0.04	0.89
Nerve_Tibial	ENSG00000143110.7	447590	414	202	1.97E-01	-0.04	0.03	0.89
Nerve_Tibial	ENSG00000273483.1	-597059	414	202	1.98E-01	0.07	0.06	0.89
Uterus	ENSG00000273483.1	-597059	111	63	1.99E-01	0.13	0.10	0.89
Pituitary	ENSG00000173947.9	575094	183	95	1.99E-01	-0.08	0.07	0.89
Uterus	ENSG00000155363.14	-751759	111	63	1.99E-01	-0.09	0.07	0.89
Whole_Blood	ENSG00000007341.14	-699443	407	195	2.01E-01	-0.07	0.05	0.89
Lung	ENSG00000134255.9	781156	427	222	2.01E-01	-0.05	0.04	0.89
Heart_Atrial_Appendage	ENSG00000162777.12	716847	297	147	2.01E-01	0.07	0.05	0.89
Brain_Cortex	ENSG00000134245.13	-545159	158	79	2.01E-01	-0.15	0.12	0.89
Esophagus_Mucosa	ENSG00000064886.9	720611	407	208	2.02E-01	0.06	0.05	0.89
Skin_Not_Sun_Exposed_Suprapubic	ENSG00000116473.10	379164	387	200	2.02E-01	-0.04	0.03	0.89
Brain_Anterior_cingulate_cortex_BA24	ENSG00000197852.8	168128	121	65	2.02E-01	-0.08	0.06	0.89
Brain_Cortex	ENSG00000273483.1	-597059	158	79	2.02E-01	0.11	0.08	0.89
Uterus	ENSG00000261654.1	979029	111	63	2.02E-01	0.15	0.12	0.89
Brain_Frontal_Cortex_BA9	ENSG00000134255.9	781156	129	73	2.03E-01	0.08	0.06	0.89
Artery_Tibial	ENSG00000260948.1	488314	441	219	2.03E-01	-0.07	0.05	0.89
Brain_Spinal_cord_cervical_c-1	ENSG00000121931.11	968421	91	49	2.04E-01	0.21	0.17	0.89
Pancreas	ENSG00000155367.11	-794095	248	108	2.04E-01	-0.08	0.07	0.89
Breast_Mammary_Tissue	ENSG00000231437.3	-68388	290	148	2.06E-01	0.09	0.07	0.89
Artery_Tibial	ENSG00000155366.12	-786052	441	219	2.06E-01	-0.03	0.02	0.89
Nerve_Tibial	ENSG00000155366.12	-786052	414	202	2.06E-01	-0.03	0.03	0.89
Brain_Hypothalamus	ENSG00000233337.1	483868	121	61	2.07E-01	0.14	0.11	0.90
Thyroid	ENSG00000143079.10	-474799	446	224	2.08E-01	0.04	0.03	0.90
Adrenal_Gland	ENSG00000007341.14	-699443	190	96	2.09E-01	-0.13	0.10	0.90
Brain_Cerebellar_Hemisphere	ENSG00000273483.1	-597059	136	74	2.11E-01	0.07	0.06	0.91
Heart_Left_Ventricle	ENSG00000156171.10	781166	303	154	2.13E-01	-0.07	0.06	0.91
Colon_Transverse	ENSG00000116489.8	-698415	274	119	2.13E-01	0.03	0.02	0.91
Spleen	ENSG00000155367.11	-794095	162	55	2.14E-01	0.13	0.10	0.91
Breast_Mammary_Tissue	ENSG00000134255.9	781156	290	148	2.16E-01	0.05	0.04	0.92
Brain_Hypothalamus	ENSG00000143110.7	447590	121	61	2.16E-01	-0.09	0.07	0.92
Uterus	ENSG00000155366.12	-786052	111	63	2.17E-01	-0.08	0.07	0.92
Colon_Transverse	ENSG00000116459.6	472490	274	119	2.18E-01	-0.03	0.02	0.92
Brain_Caudate_basal_ganglia	ENSG00000155367.11	-794095	160	88	2.18E-01	-0.10	0.08	0.92
Heart_Left_Ventricle	ENSG00000134255.9	781156	303	154	2.18E-01	-0.07	0.06	0.92
Colon_Transverse	ENSG00000238975.1	-731206	274	119	2.20E-01	0.10	0.08	0.92
Brain_Putamen_basal_ganglia	ENSG00000233337.1	483868	124	71	2.20E-01	-0.16	0.13	0.92
Skin_Sun_Exposed_Lower_leg	ENSG00000007341.14	-699443	473	233	2.21E-01	0.05	0.04	0.92
Brain_Hippocampus	ENSG00000273483.1	-597059	123	63	2.21E-01	0.10	0.08	0.92
Artery_Tibial	ENSG00000261654.1	979029	441	219	2.22E-01	-0.08	0.07	0.92
Cells_Transformed_fibroblasts	ENSG00000121931.11	968421	343	175	2.25E-01	0.04	0.04	0.93
Brain_Cortex	ENSG00000260948.1	488314	158	79	2.25E-01	0.12	0.10	0.93
Stomach	ENSG00000231346.1	312659	262	107	2.26E-01	0.07	0.06	0.93
Lung	ENSG00000227811.2	181541	427	222	2.27E-01	0.09	0.07	0.93
Skin_Sun_Exposed_Lower_leg	ENSG00000116455.9	472519	473	233	2.27E-01	-0.04	0.03	0.93
Cells_Transformed_fibroblasts	ENSG00000064886.9	720611	343	175	2.29E-01	0.05	0.04	0.93
Brain_Putamen_basal_ganglia	ENSG00000215866.3	-929261	124	71	2.29E-01	0.16	0.13	0.93

Minor_Salivary_Gland	ENSG00000162777.12	716847	97	49	2.31E-01	-0.11	0.09	0.94
Brain_Anterior_cingulate_cortex_BA24	ENSG00000273010.1	958080	121	65	2.32E-01	0.16	0.13	0.94
Esophagus_Muscularis	ENSG00000064703.7	166137	370	188	2.35E-01	0.05	0.04	0.94
Adipose_Visceral_Omentum	ENSG00000134255.9	781156	355	172	2.35E-01	0.05	0.04	0.94
Brain_Nucleus_accumbens_basal_ganglia	ENSG00000085465.11	493605	147	77	2.35E-01	0.08	0.06	0.94
Esophagus_Muscularis	ENSG00000232811.1	977915	370	188	2.35E-01	-0.10	0.08	0.94
Skin_Not_Sun_Exposed_Suprapubic	ENSG00000162777.12	716847	387	200	2.36E-01	-0.04	0.03	0.94
Brain_Cerebellum	ENSG00000116473.10	379164	173	96	2.36E-01	0.06	0.05	0.94
Colon_Transverse	ENSG00000233337.1	483868	274	119	2.36E-01	-0.10	0.09	0.94
Small_Intestine_Terminal_Ileum	ENSG00000155363.14	-751759	137	54	2.36E-01	-0.07	0.06	0.94
Brain_Putamen_basal_ganglia	ENSG00000121933.13	357420	124	71	2.37E-01	0.12	0.10	0.94
Esophagus_Gastroesophageal_Junction	ENSG00000143110.7	447590	244	110	2.39E-01	-0.06	0.05	0.94
Minor_Salivary_Gland	ENSG00000155363.14	-751759	97	49	2.39E-01	-0.10	0.08	0.94
Esophagus_Gastroesophageal_Junction	ENSG00000007341.14	-699443	244	110	2.40E-01	-0.11	0.09	0.94
Colon_Transverse	ENSG00000197852.8	168128	274	119	2.41E-01	0.07	0.06	0.94
Esophagus_Mucosa	ENSG00000261654.1	979029	407	208	2.41E-01	-0.09	0.08	0.94
Breast_Mammary_Tissue	ENSG00000171385.5	-67773	290	148	2.43E-01	-0.08	0.07	0.94
Stomach	ENSG00000197852.8	168128	262	107	2.43E-01	-0.05	0.05	0.94
Vagina	ENSG00000273010.1	958080	115	59	2.45E-01	0.17	0.15	0.94
Thyroid	ENSG00000116489.8	-698415	446	224	2.46E-01	-0.03	0.03	0.94
Lung	ENSG00000155367.11	-794095	427	222	2.46E-01	0.03	0.03	0.94
Adipose_Subcutaneous	ENSG00000116455.9	472519	442	214	2.46E-01	-0.03	0.03	0.94
Adipose_Visceral_Omentum	ENSG00000225075.1	-775239	355	172	2.46E-01	0.09	0.08	0.94
Ovary	ENSG00000261654.1	979029	133	71	2.48E-01	-0.12	0.10	0.94
Thyroid	ENSG00000227811.2	181541	446	224	2.48E-01	0.09	0.08	0.94
Brain_Anterior_cingulate_cortex_BA24	ENSG00000143079.10	-474799	121	65	2.49E-01	0.08	0.07	0.94
Stomach	ENSG00000231437.3	-68388	262	107	2.49E-01	0.07	0.06	0.94
Uterus	ENSG00000143110.7	447590	111	63	2.49E-01	0.13	0.11	0.94
Uterus	ENSG00000116459.6	472490	111	63	2.49E-01	-0.06	0.05	0.94
Adrenal_Gland	ENSG00000156171.10	781166	190	96	2.51E-01	-0.08	0.07	0.94
Artery_Tibial	ENSG00000064703.7	166137	441	219	2.51E-01	0.03	0.03	0.94
Skin_Not_Sun_Exposed_Suprapubic	ENSG00000085465.11	493605	387	200	2.52E-01	-0.04	0.03	0.94
Prostate	ENSG00000156171.10	781166	152	75	2.52E-01	-0.12	0.10	0.94
Brain_Nucleus_accumbens_basal_ganglia	ENSG00000197852.8	168128	147	77	2.52E-01	-0.11	0.10	0.94
Brain_Substantia_nigra	ENSG00000231346.1	312659	88	49	2.52E-01	0.17	0.14	0.94
Brain_Caudate_basal_ganglia	ENSG00000064703.7	166137	160	88	2.54E-01	-0.10	0.09	0.94
Brain_Spinal_cord_cervical_c-1	ENSG00000231346.1	312659	91	49	2.54E-01	-0.20	0.17	0.94
Heart_Atrial_Appendage	ENSG00000156171.10	781166	297	147	2.55E-01	-0.07	0.06	0.94
Whole_Blood	ENSG00000116473.10	379164	407	195	2.55E-01	-0.02	0.02	0.94
Brain_Substantia_nigra	ENSG00000215866.3	-929261	88	49	2.57E-01	0.15	0.13	0.94
Brain_Frontal_Cortex_BA9	ENSG00000197852.8	168128	129	73	2.57E-01	-0.07	0.06	0.94
Vagina	ENSG00000116489.8	-698415	115	59	2.58E-01	-0.07	0.06	0.94
Prostate	ENSG00000155366.12	-786052	152	75	2.59E-01	-0.09	0.08	0.94
Heart_Left_Ventricle	ENSG00000260948.1	488314	303	154	2.59E-01	0.09	0.08	0.94
Nerve_Tibial	ENSG00000231437.3	-68388	414	202	2.60E-01	0.07	0.06	0.94
Esophagus_Mucosa	ENSG00000173947.9	575094	407	208	2.61E-01	-0.06	0.05	0.94

Brain_Hypothalamus	ENSG00000121931.11	968421	121	61	2.61E-01	0.10	0.09	0.94
Adrenal_Gland	ENSG00000116473.10	379164	190	96	2.61E-01	0.06	0.06	0.94
Breast_Mammary_Tissue	ENSG00000273010.1	958080	290	148	2.61E-01	-0.10	0.09	0.94
Ovary	ENSG00000243960.1	482557	133	71	2.62E-01	-0.15	0.13	0.94
Brain_Cerebellar_Hemisphere	ENSG00000134245.13	-545159	136	74	2.63E-01	-0.08	0.07	0.94
Colon_Sigmoid	ENSG00000243960.1	482557	233	107	2.67E-01	-0.12	0.10	0.94
Esophagus_Gastroesophageal_Junction	ENSG00000116459.6	472490	244	110	2.67E-01	0.04	0.03	0.94
Esophagus_Muscularis	ENSG00000243960.1	482557	370	188	2.67E-01	0.10	0.09	0.94
Breast_Mammary_Tissue	ENSG00000232811.1	977915	290	148	2.68E-01	-0.09	0.08	0.94
Brain_Anterior_cingulate_cortex_BA24	ENSG00000134245.13	-545159	121	65	2.69E-01	-0.13	0.11	0.94
Adrenal_Gland	ENSG00000085465.11	493605	190	96	2.69E-01	-0.07	0.07	0.94
Artery_Tibial	ENSG00000116473.10	379164	441	219	2.69E-01	-0.03	0.02	0.94
Artery_Coronary	ENSG00000064886.9	720611	173	81	2.71E-01	-0.10	0.09	0.94
Cells_Transformed_fibroblasts	ENSG00000231437.3	-68388	343	175	2.72E-01	0.05	0.05	0.94
Stomach	ENSG00000261654.1	979029	262	107	2.72E-01	-0.10	0.09	0.94
Brain_Putamen_basal_ganglia	ENSG00000273483.1	-597059	124	71	2.73E-01	0.12	0.11	0.94
Brain_Putamen_basal_ganglia	ENSG00000143079.10	-474799	124	71	2.73E-01	0.09	0.08	0.94
Esophagus_Gastroesophageal_Junction	ENSG00000116473.10	379164	244	110	2.73E-01	-0.04	0.04	0.94
Uterus	ENSG00000155367.11	-794095	111	63	2.73E-01	-0.12	0.11	0.94
Brain_Anterior_cingulate_cortex_BA24	ENSG00000116459.6	472490	121	65	2.73E-01	-0.07	0.07	0.94
Pancreas	ENSG00000064703.7	166137	248	108	2.75E-01	-0.08	0.08	0.94
Pancreas	ENSG00000156171.10	781166	248	108	2.75E-01	-0.07	0.07	0.94
Lung	ENSG00000173947.9	575094	427	222	2.77E-01	-0.03	0.03	0.94
Brain_Nucleus_accumbens_basal_ganglia	ENSG00000143110.7	447590	147	77	2.78E-01	-0.08	0.07	0.94
Small_Intestine_Terminal_Ileum	ENSG00000116455.9	472519	137	54	2.78E-01	-0.07	0.07	0.94
Lung	ENSG00000197852.8	168128	427	222	2.79E-01	0.05	0.04	0.94
Liver	ENSG00000156171.10	781166	175	88	2.79E-01	-0.12	0.11	0.94
Brain_Cerebellum	ENSG00000155363.14	-751759	173	96	2.80E-01	-0.05	0.05	0.94
Brain_Frontal_Cortex_BA9	ENSG00000260948.1	488314	129	73	2.80E-01	-0.11	0.10	0.94
Adipose_Visceral_Omentum	ENSG00000162777.12	716847	355	172	2.80E-01	0.05	0.04	0.94
Minor_Salivary_Gland	ENSG00000143110.7	447590	97	49	2.81E-01	0.10	0.09	0.94
Vagina	ENSG00000155366.12	-786052	115	59	2.81E-01	0.06	0.06	0.94
Lung	ENSG00000260948.1	488314	427	222	2.81E-01	0.04	0.04	0.94
Esophagus_Mucosa	ENSG00000273483.1	-597059	407	208	2.83E-01	0.07	0.06	0.94
Spleen	ENSG00000064703.7	166137	162	55	2.83E-01	0.13	0.12	0.94
Brain_Amygdala	ENSG00000162777.12	716847	100	47	2.83E-01	-0.12	0.11	0.94
Liver	ENSG00000243960.1	482557	175	88	2.84E-01	-0.12	0.12	0.94
Skin_Not_Sun_Exposed_Suprapubic	ENSG00000143079.10	-474799	387	200	2.84E-01	0.03	0.02	0.94
Brain_Cerebellum	ENSG00000197852.8	168128	173	96	2.85E-01	-0.08	0.08	0.94
Lung	ENSG00000243960.1	482557	427	222	2.86E-01	0.07	0.07	0.94
Brain_Cerebellum	ENSG00000243960.1	482557	173	96	2.87E-01	0.12	0.11	0.94
Brain_Hypothalamus	ENSG00000156171.10	781166	121	61	2.87E-01	-0.11	0.10	0.94
Brain_Hippocampus	ENSG00000064886.9	720611	123	63	2.88E-01	0.11	0.10	0.94
Skin_Sun_Exposed_Lower_leg	ENSG00000231437.3	-68388	473	233	2.88E-01	0.05	0.05	0.94
Esophagus_Gastroesophageal_Junction	ENSG00000085465.11	493605	244	110	2.89E-01	-0.05	0.04	0.94
Colon_Transverse	ENSG00000143110.7	447590	274	119	2.89E-01	0.05	0.05	0.94
Stomach	ENSG00000143079.10	-474799	262	107	2.89E-01	-0.04	0.04	0.94

Brain_Caudate_basal_ganglia	ENSG00000143110.7	447590	160	88	2.89E-01	-0.06	0.06	0.94
Brain_Frontal_Cortex_BA9	ENSG00000231437.3	-68388	129	73	2.90E-01	0.13	0.12	0.94
Brain_Cortex	ENSG00000143079.10	-474799	158	79	2.92E-01	0.09	0.08	0.94
Cells_EBV-transformed_lymphocytes	ENSG00000238975.1	-731206	130	68	2.92E-01	-0.10	0.09	0.94
Breast_Mammary_Tissue	ENSG00000143079.10	-474799	290	148	2.93E-01	0.03	0.03	0.94
Nerve_Tibial	ENSG00000116473.10	379164	414	202	2.94E-01	-0.04	0.03	0.94
Liver	ENSG00000232811.1	977915	175	88	2.94E-01	0.12	0.11	0.94
Minor_Salivary_Gland	ENSG00000155366.12	-786052	97	49	2.95E-01	0.06	0.06	0.94
Pancreas	ENSG00000238975.1	-731206	248	108	2.95E-01	-0.11	0.11	0.94
Brain_Amygdala	ENSG00000171385.5	-67773	100	47	2.95E-01	-0.07	0.07	0.94
Colon_Sigmoid	ENSG00000225075.1	-775239	233	107	2.96E-01	-0.11	0.10	0.94
Artery_Tibial	ENSG00000134255.9	781156	441	219	2.96E-01	-0.04	0.04	0.94
Brain_Caudate_basal_ganglia	ENSG00000007341.14	-699443	160	88	2.97E-01	-0.12	0.11	0.94
Testis	ENSG00000134255.9	781156	259	128	2.97E-01	0.03	0.03	0.94
Heart_Atrial_Appendage	ENSG00000064703.7	166137	297	147	2.97E-01	0.04	0.04	0.94
Adipose_Visceral_Omentum	ENSG00000243960.1	482557	355	172	2.98E-01	-0.07	0.07	0.94
Artery_Aorta	ENSG00000007341.14	-699443	299	152	2.98E-01	-0.06	0.06	0.94
Adrenal_Gland	ENSG00000231437.3	-68388	190	96	2.99E-01	0.10	0.10	0.94
Adipose_Visceral_Omentum	ENSG00000116489.8	-698415	355	172	2.99E-01	-0.03	0.03	0.94
Breast_Mammary_Tissue	ENSG00000238975.1	-731206	290	148	2.99E-01	-0.08	0.08	0.94
Brain_Cerebellar_Hemisphere	ENSG00000273010.1	958080	136	74	3.00E-01	0.13	0.12	0.94
Liver	ENSG00000155363.14	-751759	175	88	3.01E-01	-0.07	0.06	0.94
Muscle_Skeletal	ENSG00000197852.8	168128	564	276	3.01E-01	-0.04	0.04	0.94
Artery_Aorta	ENSG00000121933.13	357420	299	152	3.01E-01	-0.04	0.04	0.94
Brain_Nucleus_accumbens_basal_ganglia	ENSG00000155363.14	-751759	147	77	3.01E-01	-0.08	0.07	0.94
Colon_Sigmoid	ENSG00000162777.12	716847	233	107	3.02E-01	0.06	0.05	0.94
Spleen	ENSG00000273483.1	-597059	162	55	3.02E-01	0.12	0.11	0.94
Liver	ENSG00000261654.1	979029	175	88	3.02E-01	-0.14	0.13	0.94
Artery_Aorta	ENSG00000116473.10	379164	299	152	3.02E-01	-0.04	0.04	0.94
Artery_Tibial	ENSG00000197852.8	168128	441	219	3.02E-01	-0.04	0.04	0.94
Ovary	ENSG00000225075.1	-775239	133	71	3.02E-01	0.13	0.12	0.94
Heart_Left_Ventricle	ENSG00000155366.12	-786052	303	154	3.03E-01	-0.03	0.03	0.94
Uterus	ENSG00000171385.5	-67773	111	63	3.03E-01	-0.11	0.11	0.94
Testis	ENSG00000232811.1	977915	259	128	3.05E-01	-0.07	0.07	0.94
Ovary	ENSG00000273010.1	958080	133	71	3.05E-01	0.13	0.12	0.94
Adipose_Visceral_Omentum	ENSG00000064886.9	720611	355	172	3.06E-01	-0.06	0.06	0.94
Skin_Not_Sun_Exposed_Suprapubic	ENSG00000116459.6	472490	387	200	3.06E-01	0.03	0.02	0.94
Uterus	ENSG00000233337.1	483868	111	63	3.06E-01	0.12	0.11	0.94
Brain_Cerebellum	ENSG00000064703.7	166137	173	96	3.06E-01	-0.08	0.08	0.94
Brain_Substantia_nigra	ENSG00000121933.13	357420	88	49	3.07E-01	0.07	0.07	0.94
Brain_Frontal_Cortex_BA9	ENSG00000064886.9	720611	129	73	3.08E-01	0.11	0.10	0.94
Cells_Transformed_fibroblasts	ENSG00000231346.1	312659	343	175	3.08E-01	0.07	0.07	0.94
Muscle_Skeletal	ENSG00000162777.12	716847	564	276	3.08E-01	-0.04	0.04	0.94
Cells_EBV-transformed_lymphocytes	ENSG00000116459.6	472490	130	68	3.09E-01	0.05	0.05	0.94
Testis	ENSG00000231437.3	-68388	259	128	3.09E-01	0.08	0.08	0.94
Brain_Cortex	ENSG00000007341.14	-699443	158	79	3.09E-01	0.11	0.11	0.94
Heart_Atrial_Appendage	ENSG00000007341.14	-699443	297	147	3.11E-01	-0.08	0.08	0.94

Brain_Nucleus_accumbens_basal_ganglia	ENSG00000231246.1	-439146	147	77	3.11E-01	-0.12	0.12	0.94
Brain_Frontal_Cortex_BA9	ENSG00000007341.14	-699443	129	73	3.12E-01	-0.13	0.12	0.94
Artery_Coronary	ENSG00000121933.13	357420	173	81	3.16E-01	-0.06	0.06	0.94
Brain_Substantia_nigra	ENSG00000116455.9	472519	88	49	3.16E-01	0.10	0.10	0.94
Brain_Spinal_cord_cervical_c-1	ENSG00000215866.3	-929261	91	49	3.16E-01	-0.13	0.13	0.94
Adipose_Subcutaneous	ENSG00000156171.10	781166	442	214	3.16E-01	-0.05	0.04	0.94
Brain_Nucleus_accumbens_basal_ganglia	ENSG00000116473.10	379164	147	77	3.17E-01	-0.05	0.05	0.94
Muscle_Skeletal	ENSG00000116489.8	-698415	564	276	3.17E-01	0.03	0.03	0.94
Brain_Cortex	ENSG00000116473.10	379164	158	79	3.18E-01	-0.06	0.06	0.94
Brain_Cortex	ENSG00000155363.14	-751759	158	79	3.19E-01	-0.06	0.06	0.94
Prostate	ENSG00000184599.9	-799037	152	75	3.20E-01	-0.10	0.10	0.94
Artery_Aorta	ENSG00000273483.1	-597059	299	152	3.20E-01	0.07	0.07	0.94
Brain_Hippocampus	ENSG00000232811.1	977915	123	63	3.21E-01	-0.15	0.15	0.94
Pancreas	ENSG00000243960.1	482557	248	108	3.23E-01	0.09	0.09	0.94
Minor_Salivary_Gland	ENSG00000134245.13	-545159	97	49	3.24E-01	-0.10	0.10	0.94
Brain_Frontal_Cortex_BA9	ENSG00000227811.2	181541	129	73	3.25E-01	0.12	0.12	0.94
Skin_Sun_Exposed_Lower_leg	ENSG00000173947.9	575094	473	233	3.25E-01	0.05	0.05	0.94
Pituitary	ENSG00000116473.10	379164	183	95	3.26E-01	-0.06	0.06	0.94
Esophagus_Gastroesophageal_Junction	ENSG00000225075.1	-775239	244	110	3.26E-01	-0.10	0.10	0.94
Pituitary	ENSG00000231437.3	-68388	183	95	3.26E-01	0.10	0.10	0.94
Artery_Aorta	ENSG00000231437.3	-68388	299	152	3.26E-01	0.06	0.06	0.94
Pancreas	ENSG00000232811.1	977915	248	108	3.26E-01	0.11	0.11	0.94
Thyroid	ENSG00000173947.9	575094	446	224	3.27E-01	0.03	0.04	0.94
Thyroid	ENSG00000273483.1	-597059	446	224	3.27E-01	-0.06	0.06	0.94
Pituitary	ENSG00000121931.11	968421	183	95	3.28E-01	0.08	0.08	0.94
Artery_Coronary	ENSG00000260948.1	488314	173	81	3.29E-01	-0.09	0.09	0.94
Brain_Hypothalamus	ENSG00000184599.9	-799037	121	61	3.29E-01	0.14	0.14	0.94
Brain_Cerebellum	ENSG00000232558.1	64477	173	96	3.30E-01	0.12	0.12	0.94
Heart_Left_Ventricle	ENSG00000155367.11	-794095	303	154	3.30E-01	0.05	0.05	0.94
Colon_Sigmoid	ENSG00000116473.10	379164	233	107	3.30E-01	-0.05	0.06	0.94
Breast_Mammary_Tissue	ENSG00000156171.10	781166	290	148	3.30E-01	0.04	0.04	0.94
Artery_Coronary	ENSG00000121931.11	968421	173	81	3.31E-01	-0.09	0.10	0.94
Ovary	ENSG00000116473.10	379164	133	71	3.31E-01	-0.07	0.07	0.94
Colon_Sigmoid	ENSG00000173947.9	575094	233	107	3.32E-01	-0.06	0.06	0.94
Cells_Transformed_fibroblasts	ENSG00000224167.1	-955558	343	175	3.32E-01	0.07	0.08	0.94
Colon_Sigmoid	ENSG00000171385.5	-67773	233	107	3.33E-01	-0.07	0.07	0.94
Artery_Tibial	ENSG00000121931.11	968421	441	219	3.34E-01	-0.04	0.04	0.94
Heart_Left_Ventricle	ENSG00000162777.12	716847	303	154	3.34E-01	-0.05	0.05	0.94
Adipose_Subcutaneous	ENSG00000231437.3	-68388	442	214	3.35E-01	0.05	0.06	0.94
Nerve_Tibial	ENSG00000064886.9	720611	414	202	3.35E-01	-0.05	0.05	0.94
Colon_Sigmoid	ENSG00000116489.8	-698415	233	107	3.36E-01	-0.05	0.06	0.94
Artery_Aorta	ENSG00000261654.1	979029	299	152	3.36E-01	0.08	0.08	0.94
Adipose_Subcutaneous	ENSG00000134216.14	630520	442	214	3.37E-01	-0.06	0.07	0.94
Brain_Hippocampus	ENSG00000155363.14	-751759	123	63	3.38E-01	-0.06	0.06	0.94
Artery_Tibial	ENSG00000143079.10	-474799	441	219	3.38E-01	-0.03	0.03	0.94
Vagina	ENSG00000134255.9	781156	115	59	3.39E-01	-0.08	0.09	0.94

Vagina	ENSG00000162777.12	716847	115	59	3.39E-01	0.05	0.05	0.94
Thyroid	ENSG00000116459.6	472490	446	224	3.39E-01	-0.01	0.01	0.94
Brain_Putamen_basal_ganglia	ENSG00000155367.11	-794095	124	71	3.39E-01	-0.10	0.10	0.94
Ovary	ENSG00000155363.14	-751759	133	71	3.40E-01	0.06	0.07	0.94
Brain_Hippocampus	ENSG00000171385.5	-67773	123	63	3.42E-01	0.06	0.06	0.94
Muscle_Skeletal	ENSG00000007341.14	-699443	564	276	3.42E-01	-0.05	0.05	0.94
Brain_Amygdala	ENSG00000227811.2	181541	100	47	3.43E-01	-0.17	0.18	0.94
Prostate	ENSG00000260948.1	488314	152	75	3.43E-01	0.10	0.10	0.94
Thyroid	ENSG00000155367.11	-794095	446	224	3.43E-01	-0.04	0.04	0.94
Pituitary	ENSG00000224167.1	-955558	183	95	3.44E-01	-0.14	0.14	0.94
Brain_Frontal_Cortex_BA9	ENSG00000273483.1	-597059	129	73	3.45E-01	0.07	0.07	0.94
Brain_Spinal_cord_cervical_c-1	ENSG00000184599.9	-799037	91	49	3.45E-01	-0.14	0.15	0.94
Testis	ENSG00000215866.3	-929261	259	128	3.45E-01	0.04	0.04	0.94
Brain_Hippocampus	ENSG00000224167.1	-955558	123	63	3.45E-01	-0.13	0.14	0.94
Adipose_Subcutaneous	ENSG00000243960.1	482557	442	214	3.46E-01	-0.07	0.08	0.94
Nerve_Tibial	ENSG00000171385.5	-67773	414	202	3.47E-01	0.04	0.05	0.94
Artery_Coronary	ENSG00000134255.9	781156	173	81	3.47E-01	-0.06	0.06	0.94
Uterus	ENSG00000116473.10	379164	111	63	3.47E-01	-0.05	0.06	0.94
Minor_Salivary_Gland	ENSG00000184599.9	-799037	97	49	3.49E-01	0.13	0.14	0.94
Cells_Transformed_fibroblasts	ENSG00000155363.14	-751759	343	175	3.49E-01	-0.03	0.03	0.94
Brain_Caudate_basal_ganglia	ENSG00000156171.10	781166	160	88	3.49E-01	-0.07	0.08	0.94
Stomach	ENSG00000233337.1	483868	262	107	3.50E-01	-0.07	0.08	0.94
Whole_Blood	ENSG00000143110.7	447590	407	195	3.50E-01	-0.02	0.02	0.94
Adrenal_Gland	ENSG00000243960.1	482557	190	96	3.50E-01	-0.10	0.11	0.94
Adipose_Subcutaneous	ENSG00000227179.2	529563	442	214	3.52E-01	0.05	0.05	0.94
Lung	ENSG00000064886.9	720611	427	222	3.52E-01	0.05	0.05	0.94
Brain_Hippocampus	ENSG00000116473.10	379164	123	63	3.53E-01	-0.05	0.05	0.94
Brain_Spinal_cord_cervical_c-1	ENSG00000007341.14	-699443	91	49	3.53E-01	-0.14	0.15	0.94
Minor_Salivary_Gland	ENSG00000231437.3	-68388	97	49	3.53E-01	-0.11	0.12	0.94
Brain_Hippocampus	ENSG00000162777.12	716847	123	63	3.55E-01	0.09	0.10	0.94
Prostate	ENSG00000064886.9	720611	152	75	3.55E-01	-0.08	0.08	0.94
Liver	ENSG00000197852.8	168128	175	88	3.56E-01	-0.10	0.10	0.94
Brain_Spinal_cord_cervical_c-1	ENSG00000224167.1	-955558	91	49	3.57E-01	-0.15	0.16	0.94
Adipose_Visceral_Omentum	ENSG00000231246.1	-439146	355	172	3.57E-01	0.07	0.07	0.94
Brain_Nucleus_accumbens_basal_ganglia	ENSG00000121933.13	357420	147	77	3.58E-01	-0.07	0.07	0.94
Brain_Amygdala	ENSG00000224167.1	-955558	100	47	3.58E-01	-0.15	0.16	0.94
Cells_Transformed_fibroblasts	ENSG00000238975.1	-731206	343	175	3.58E-01	-0.07	0.08	0.94
Stomach	ENSG00000243960.1	482557	262	107	3.58E-01	-0.08	0.08	0.94
Esophagus_Gastroesophageal_Junction	ENSG00000121931.11	968421	244	110	3.58E-01	-0.06	0.07	0.94
Vagina	ENSG00000231346.1	312659	115	59	3.59E-01	0.08	0.08	0.94
Brain_Frontal_Cortex_BA9	ENSG00000121933.13	357420	129	73	3.59E-01	0.09	0.10	0.94
Artery_Tibial	ENSG00000064886.9	720611	441	219	3.61E-01	-0.05	0.05	0.94
Brain_Cortex	ENSG00000233337.1	483868	158	79	3.62E-01	-0.12	0.13	0.94
Artery_Coronary	ENSG00000197852.8	168128	173	81	3.62E-01	-0.06	0.06	0.94
Brain_Substantia_nigra	ENSG00000156171.10	781166	88	49	3.63E-01	0.09	0.10	0.94
Ovary	ENSG00000007341.14	-699443	133	71	3.63E-01	0.08	0.08	0.94
Liver	ENSG00000134245.13	-545159	175	88	3.64E-01	-0.09	0.10	0.94

Skin_Sun_Exposed_Lower_leg	ENSG00000231246.1	-439146	473	233	3.64E-01	0.05	0.06	0.94
Testis	ENSG00000234020.1	553973	259	128	3.65E-01	-0.08	0.09	0.94
Testis	ENSG00000121933.13	357420	259	128	3.66E-01	-0.03	0.03	0.94
Adipose_Visceral_Omentum	ENSG00000134245.13	-545159	355	172	3.66E-01	-0.03	0.04	0.94
Brain_Caudate_basal_ganglia	ENSG00000273483.1	-597059	160	88	3.67E-01	0.07	0.08	0.94
Breast_Mammary_Tissue	ENSG00000143110.7	447590	290	148	3.67E-01	-0.03	0.03	0.94
Uterus	ENSG00000116455.9	472519	111	63	3.68E-01	0.05	0.05	0.94
Heart_Left_Ventricle	ENSG00000273010.1	958080	303	154	3.69E-01	0.08	0.09	0.94
Adrenal_Gland	ENSG00000215866.3	-929261	190	96	3.70E-01	-0.09	0.10	0.94
Uterus	ENSG00000184599.9	-799037	111	63	3.71E-01	-0.10	0.12	0.94
Brain_Substantia_nigra	ENSG00000155363.14	-751759	88	49	3.71E-01	0.09	0.10	0.94
Artery_Aorta	ENSG00000134245.13	-545159	299	152	3.72E-01	-0.05	0.06	0.94
Uterus	ENSG00000007341.14	-699443	111	63	3.73E-01	-0.07	0.08	0.94
Colon_Sigmoid	ENSG00000155366.12	-786052	233	107	3.73E-01	0.04	0.05	0.94
Brain_Nucleus_accumbens_basal_ganglia	ENSG00000064703.7	166137	147	77	3.73E-01	-0.09	0.10	0.94
Adrenal_Gland	ENSG00000232811.1	977915	190	96	3.74E-01	0.09	0.10	0.94
Breast_Mammary_Tissue	ENSG00000116455.9	472519	290	148	3.74E-01	0.03	0.03	0.94
Adrenal_Gland	ENSG00000173947.9	575094	190	96	3.74E-01	-0.05	0.06	0.94
Skin_Not_Sun_Exposed_Suprapubic	ENSG00000116455.9	472519	387	200	3.75E-01	0.03	0.03	0.94
Skin_Sun_Exposed_Lower_leg	ENSG00000156171.10	781166	473	233	3.75E-01	-0.04	0.04	0.94
Colon_Sigmoid	ENSG00000261654.1	979029	233	107	3.76E-01	0.08	0.09	0.94
Brain_Nucleus_accumbens_basal_ganglia	ENSG00000155366.12	-786052	147	77	3.76E-01	-0.04	0.05	0.94
Esophagus_Muscularis	ENSG00000155367.11	-794095	370	188	3.78E-01	0.04	0.04	0.94
Whole_Blood	ENSG00000231346.1	312659	407	195	3.78E-01	0.04	0.05	0.94
Brain_Amygdala	ENSG00000243960.1	482557	100	47	3.79E-01	-0.18	0.20	0.94
Brain_Cerebellum	ENSG00000227811.2	181541	173	96	3.79E-01	0.11	0.13	0.94
Spleen	ENSG00000171385.5	-67773	162	55	3.80E-01	-0.10	0.12	0.94
Brain_Cerebellum	ENSG00000224167.1	-955558	173	96	3.80E-01	0.12	0.13	0.94
Heart_Left_Ventricle	ENSG00000134245.13	-545159	303	154	3.80E-01	-0.05	0.06	0.94
Esophagus_Mucosa	ENSG00000260948.1	488314	407	208	3.82E-01	0.05	0.06	0.94
Brain_Spinal_cord_cervical_c-1	ENSG00000143079.10	-474799	91	49	3.83E-01	0.10	0.11	0.94
Skin_Not_Sun_Exposed_Suprapubic	ENSG00000143110.7	447590	387	200	3.84E-01	-0.04	0.04	0.94
Thyroid	ENSG00000155363.14	-751759	446	224	3.84E-01	-0.02	0.03	0.94
Pancreas	ENSG00000225075.1	-775239	248	108	3.84E-01	-0.08	0.09	0.94
Pancreas	ENSG00000231437.3	-68388	248	108	3.84E-01	0.06	0.07	0.94
Adipose_Visceral_Omentum	ENSG00000116459.6	472490	355	172	3.84E-01	0.02	0.02	0.94
Brain_Substantia_nigra	ENSG00000155367.11	-794095	88	49	3.85E-01	0.07	0.07	0.94
Brain_Cortex	ENSG00000064886.9	720611	158	79	3.85E-01	-0.08	0.09	0.94
Heart_Atrial_Appendage	ENSG00000155366.12	-786052	297	147	3.85E-01	-0.03	0.04	0.94
Brain_Putamen_basal_ganglia	ENSG00000155363.14	-751759	124	71	3.85E-01	-0.06	0.07	0.94
Thyroid	ENSG00000143110.7	447590	446	224	3.86E-01	0.02	0.03	0.94
Esophagus_Muscularis	ENSG00000143079.10	-474799	370	188	3.86E-01	-0.05	0.05	0.94
Cells_EBV-transformed_lymphocytes	ENSG00000225075.1	-775239	130	68	3.86E-01	0.10	0.11	0.94
Heart_Left_Ventricle	ENSG00000116489.8	-698415	303	154	3.87E-01	-0.03	0.03	0.94
Nerve_Tibial	ENSG00000225075.1	-775239	414	202	3.88E-01	0.07	0.08	0.94
Cells_EBV-transformed_lymphocytes	ENSG00000064886.9	720611	130	68	3.89E-01	-0.11	0.12	0.94

Colon_Sigmoid	ENSG00000134255.9	781156	233	107	3.89E-01	-0.05	0.06	0.94
Colon_Transverse	ENSG00000171385.5	-67773	274	119	3.89E-01	-0.04	0.04	0.94
Adipose_Subcutaneous	ENSG00000116489.8	-698415	442	214	3.89E-01	-0.02	0.02	0.94
Esophagus_Gastroesophageal_Junction	ENSG00000231246.1	-439146	244	110	3.90E-01	-0.07	0.08	0.94
Vagina	ENSG00000173947.9	575094	115	59	3.90E-01	0.08	0.10	0.94
Adipose_Visceral_Omentum	ENSG00000116455.9	472519	355	172	3.91E-01	0.02	0.02	0.94
Esophagus_Gastroesophageal_Junction	ENSG00000173947.9	575094	244	110	3.92E-01	-0.05	0.06	0.94
Brain_Frontal_Cortex_BA9	ENSG00000184599.9	-799037	129	73	3.93E-01	-0.12	0.14	0.94
Brain_Amygdala	ENSG00000215866.3	-929261	100	47	3.93E-01	0.11	0.13	0.94
Minor_Salivary_Gland	ENSG00000243960.1	482557	97	49	3.93E-01	0.10	0.11	0.94
Brain_Substantia_nigra	ENSG00000231246.1	-439146	88	49	3.95E-01	-0.13	0.15	0.94
Adipose_Visceral_Omentum	ENSG00000143079.10	-474799	355	172	3.95E-01	-0.03	0.03	0.94
Brain_Cerebellar_Hemisphere	ENSG00000007341.14	-699443	136	74	3.95E-01	-0.09	0.10	0.94
Testis	ENSG00000227811.2	181541	259	128	3.96E-01	-0.07	0.08	0.94
Brain_Spinal_cord_cervical_c-1	ENSG00000143110.7	447590	91	49	3.96E-01	0.07	0.08	0.94
Brain_Cerebellar_Hemisphere	ENSG00000224167.1	-955558	136	74	3.96E-01	-0.10	0.12	0.94
Testis	ENSG00000171385.5	-67773	259	128	3.96E-01	0.07	0.08	0.94
Brain_Spinal_cord_cervical_c-1	ENSG00000261654.1	979029	91	49	3.97E-01	-0.13	0.16	0.94
Pancreas	ENSG00000231346.1	312659	248	108	3.97E-01	0.08	0.09	0.94
Esophagus_Muscularis	ENSG00000197852.8	168128	370	188	3.97E-01	-0.04	0.05	0.94
Cells_EBV-transformed_lymphocytes	ENSG00000243960.1	482557	130	68	3.98E-01	-0.11	0.13	0.94
Artery_Aorta	ENSG00000121931.11	968421	299	152	3.98E-01	-0.04	0.05	0.94
Cells_Transformed_fibroblasts	ENSG00000143110.7	447590	343	175	3.99E-01	0.06	0.07	0.94
Minor_Salivary_Gland	ENSG00000273010.1	958080	97	49	4.00E-01	-0.12	0.14	0.94
Brain_Hypothalamus	ENSG00000155367.11	-794095	121	61	4.00E-01	0.07	0.09	0.94
Muscle_Skeletal	ENSG00000134255.9	781156	564	276	4.01E-01	0.04	0.04	0.94
Brain_Cerebellar_Hemisphere	ENSG00000197852.8	168128	136	74	4.01E-01	-0.08	0.09	0.94
Heart_Atrial_Appendage	ENSG00000260948.1	488314	297	147	4.01E-01	0.06	0.07	0.94
Ovary	ENSG00000064886.9	720611	133	71	4.03E-01	-0.08	0.09	0.94
Artery_Aorta	ENSG00000155367.11	-794095	299	152	4.03E-01	-0.03	0.04	0.94
Brain_Substantia_nigra	ENSG00000233337.1	483868	88	49	4.03E-01	-0.13	0.15	0.94
Artery_Coronary	ENSG00000116473.10	379164	173	81	4.04E-01	0.04	0.05	0.94
Thyroid	ENSG00000261654.1	979029	446	224	4.05E-01	0.05	0.06	0.94
Colon_Sigmoid	ENSG00000232811.1	977915	233	107	4.05E-01	0.09	0.11	0.94
Esophagus_Mucosa	ENSG00000116455.9	472519	407	208	4.05E-01	0.03	0.03	0.94
Liver	ENSG00000064886.9	720611	175	88	4.06E-01	0.10	0.12	0.94
Breast_Mammary_Tissue	ENSG00000116489.8	-698415	290	148	4.06E-01	-0.03	0.03	0.94
Small_Intestine_Terminal_Ileum	ENSG00000116489.8	-698415	137	54	4.07E-01	-0.06	0.07	0.94
Testis	ENSG00000260948.1	488314	259	128	4.07E-01	0.05	0.07	0.94
Cells_EBV-transformed_lymphocytes	ENSG00000143079.10	-474799	130	68	4.07E-01	-0.08	0.10	0.94
Brain_Cerebellar_Hemisphere	ENSG00000064703.7	166137	136	74	4.07E-01	-0.07	0.08	0.94
Brain_Cerebellar_Hemisphere	ENSG00000121931.11	968421	136	74	4.08E-01	0.07	0.09	0.94
Artery_Tibial	ENSG00000273483.1	-597059	441	219	4.08E-01	-0.05	0.07	0.94
Spleen	ENSG00000197852.8	168128	162	55	4.08E-01	-0.07	0.08	0.94
Cells_EBV-transformed_lymphocytes	ENSG00000064703.7	166137	130	68	4.09E-01	0.05	0.07	0.94
Prostate	ENSG00000116455.9	472519	152	75	4.10E-01	-0.05	0.06	0.94
Pituitary	ENSG00000232811.1	977915	183	95	4.11E-01	0.11	0.13	0.94

Brain_Cerebellum	ENSG00000171385.5	-67773	173	96	4.11E-01	0.05	0.06	0.94
Skin_Sun_Exposed_Lower_leg	ENSG00000197852.8	168128	473	233	4.11E-01	0.03	0.04	0.94
Lung	ENSG00000261654.1	979029	427	222	4.12E-01	0.04	0.05	0.94
Skin_Not_Sun_Exposed_Suprapubic	ENSG00000231246.1	-439146	387	200	4.13E-01	0.05	0.07	0.94
Brain_Amygdala	ENSG00000155363.14	-751759	100	47	4.14E-01	-0.08	0.10	0.94
Breast_Mammary_Tissue	ENSG00000116459.6	472490	290	148	4.14E-01	-0.02	0.02	0.94
Nerve_Tibial	ENSG00000260948.1	488314	414	202	4.15E-01	0.04	0.05	0.94
Small_Intestine_Terminal_Ileum	ENSG00000171385.5	-67773	137	54	4.15E-01	0.07	0.08	0.94
Vagina	ENSG00000233337.1	483868	115	59	4.15E-01	0.11	0.14	0.94
Brain_Cerebellum	ENSG00000121931.11	968421	173	96	4.17E-01	0.06	0.07	0.94
Muscle_Skeletal	ENSG00000085465.11	493605	564	276	4.18E-01	0.03	0.03	0.94
Brain_Putamen_basal_ganglia	ENSG00000134245.13	-545159	124	71	4.18E-01	0.04	0.05	0.94
Brain_Putamen_basal_ganglia	ENSG00000085465.11	493605	124	71	4.18E-01	0.06	0.07	0.94
Adipose_Subcutaneous	ENSG00000225075.1	-775239	442	214	4.23E-01	-0.06	0.07	0.94
Esophagus_Mucosa	ENSG00000232811.1	977915	407	208	4.24E-01	-0.06	0.08	0.94
Skin_Not_Sun_Exposed_Suprapubic	ENSG00000155363.14	-751759	387	200	4.25E-01	0.02	0.03	0.94
Brain_Cerebellum	ENSG00000156171.10	781166	173	96	4.26E-01	0.07	0.09	0.94
Spleen	ENSG00000116489.8	-698415	162	55	4.26E-01	0.05	0.07	0.94
Nerve_Tibial	ENSG00000173947.9	575094	414	202	4.26E-01	0.04	0.05	0.94
Prostate	ENSG00000273483.1	-597059	152	75	4.26E-01	-0.06	0.08	0.94
Whole_Blood	ENSG00000134245.13	-545159	407	195	4.27E-01	-0.05	0.06	0.94
Brain_Anterior_cingulate_cortex_BA24	ENSG00000184599.9	-799037	121	65	4.27E-01	0.10	0.12	0.94
Brain_Substantia_nigra	ENSG00000171385.5	-67773	88	49	4.27E-01	0.05	0.06	0.94
Brain_Cerebellar_Hemisphere	ENSG00000134255.9	781156	136	74	4.28E-01	-0.05	0.06	0.94
Brain_Hypothalamus	ENSG00000064886.9	720611	121	61	4.28E-01	0.08	0.10	0.94
Heart_Atrial_Appendage	ENSG00000064886.9	720611	297	147	4.29E-01	-0.05	0.07	0.94
Adipose_Visceral_Omentum	ENSG00000007341.14	-699443	355	172	4.29E-01	-0.05	0.07	0.94
Brain_Substantia_nigra	ENSG00000116489.8	-698415	88	49	4.30E-01	0.07	0.09	0.94
Stomach	ENSG00000155363.14	-751759	262	107	4.31E-01	-0.04	0.05	0.94
Adipose_Subcutaneous	ENSG00000173947.9	575094	442	214	4.31E-01	-0.04	0.05	0.94
Brain_Anterior_cingulate_cortex_BA24	ENSG00000273483.1	-597059	121	65	4.31E-01	0.07	0.08	0.94
Brain_Cortex	ENSG00000171385.5	-67773	158	79	4.32E-01	-0.05	0.06	0.94
Cells_Transformed_fibroblasts	ENSG00000116459.6	472490	343	175	4.32E-01	-0.01	0.02	0.94
Minor_Salivary_Gland	ENSG00000116489.8	-698415	97	49	4.33E-01	-0.05	0.06	0.94
Brain_Hypothalamus	ENSG00000171385.5	-67773	121	61	4.33E-01	0.07	0.09	0.94
Brain_Cortex	ENSG00000143110.7	447590	158	79	4.34E-01	0.06	0.07	0.94
Skin_Sun_Exposed_Lower_leg	ENSG00000232811.1	977915	473	233	4.35E-01	0.06	0.07	0.95
Stomach	ENSG00000116489.8	-698415	262	107	4.36E-01	-0.03	0.04	0.95
Small_Intestine_Terminal_Ileum	ENSG00000225075.1	-775239	137	54	4.37E-01	0.07	0.10	0.95
Colon_Sigmoid	ENSG00000134245.13	-545159	233	107	4.38E-01	-0.05	0.06	0.95
Spleen	ENSG00000007341.14	-699443	162	55	4.39E-01	0.10	0.12	0.95
Brain_Nucleus_accumbens_basal_ganglia	ENSG00000215866.3	-929261	147	77	4.41E-01	0.09	0.11	0.95
Brain_Cerebellum	ENSG00000273483.1	-597059	173	96	4.43E-01	0.06	0.08	0.95
Brain_Amygdala	ENSG00000233337.1	483868	100	47	4.43E-01	-0.15	0.20	0.95
Cells_Transformed_fibroblasts	ENSG00000231246.1	-439146	343	175	4.44E-01	-0.04	0.06	0.95
Skin_Sun_Exposed_Lower_leg	ENSG00000143110.7	447590	473	233	4.45E-01	-0.02	0.03	0.95
Skin_Sun_Exposed_Lower_leg	ENSG00000243960.1	482557	473	233	4.45E-01	0.05	0.07	0.95

Brain_Amygdala	ENSG00000155367.11	-794095	100	47	4.45E-01	0.11	0.15	0.95
Brain_Amygdala	ENSG00000156171.10	781166	100	47	4.46E-01	-0.09	0.12	0.95
Brain_Nucleus_accumbens_basal_ganglia	ENSG00000007341.14	-699443	147	77	4.47E-01	-0.10	0.13	0.95
Brain_Hypothalamus	ENSG00000064703.7	166137	121	61	4.47E-01	-0.08	0.11	0.95
Colon_Transverse	ENSG00000273483.1	-597059	274	119	4.47E-01	0.05	0.07	0.95
Stomach	ENSG00000085465.11	493605	262	107	4.49E-01	-0.05	0.06	0.95
Adipose_Subcutaneous	ENSG00000007341.14	-699443	442	214	4.51E-01	-0.05	0.06	0.95
Small_Intestine_Terminal_Ileum	ENSG00000064886.9	720611	137	54	4.51E-01	0.06	0.08	0.95
Brain_Amygdala	ENSG00000197852.8	168128	100	47	4.52E-01	0.07	0.09	0.95
Brain_Spinal_cord_cervical_c-1	ENSG00000156171.10	781166	91	49	4.52E-01	-0.08	0.11	0.95
Stomach	ENSG00000260948.1	488314	262	107	4.52E-01	-0.06	0.08	0.95
Brain_Anterior_cingulate_cortex_BA24	ENSG00000155363.14	-751759	121	65	4.52E-01	-0.04	0.05	0.95
Small_Intestine_Terminal_Ileum	ENSG00000162777.12	716847	137	54	4.53E-01	-0.04	0.05	0.95
Esophagus_Mucosa	ENSG00000064703.7	166137	407	208	4.53E-01	0.03	0.03	0.95
Brain_Putamen_basal_ganglia	ENSG00000064886.9	720611	124	71	4.53E-01	-0.09	0.12	0.95
Brain_Cerebellum	ENSG00000261654.1	979029	173	96	4.54E-01	0.07	0.10	0.95
Adipose_Visceral_Omentum	ENSG00000143110.7	447590	355	172	4.55E-01	-0.02	0.03	0.95
Brain_Caudate_basal_ganglia	ENSG00000085465.11	493605	160	88	4.55E-01	0.05	0.06	0.95
Liver	ENSG00000143079.10	-474799	175	88	4.55E-01	-0.05	0.06	0.95
Vagina	ENSG00000143079.10	-474799	115	59	4.56E-01	0.05	0.07	0.95
Brain_Cerebellar_Hemisphere	ENSG00000121933.13	357420	136	74	4.56E-01	-0.08	0.11	0.95
Testis	ENSG00000143110.7	447590	259	128	4.57E-01	0.06	0.07	0.95
Testis	ENSG00000116473.10	379164	259	128	4.57E-01	-0.02	0.02	0.95
Muscle_Skeletal	ENSG00000134245.13	-545159	564	276	4.59E-01	0.03	0.05	0.95
Adrenal_Gland	ENSG00000233337.1	483868	190	96	4.60E-01	-0.07	0.09	0.95
Brain_Frontal_Cortex_BA9	ENSG00000273010.1	958080	129	73	4.61E-01	-0.10	0.13	0.95
Adrenal_Gland	ENSG00000184599.9	-799037	190	96	4.62E-01	0.04	0.05	0.95
Vagina	ENSG00000238975.1	-731206	115	59	4.62E-01	0.11	0.15	0.95
Heart_Left_Ventricle	ENSG00000121933.13	357420	303	154	4.63E-01	-0.04	0.05	0.95
Adipose_Visceral_Omentum	ENSG00000260948.1	488314	355	172	4.64E-01	0.05	0.06	0.95
Lung	ENSG00000134216.14	630520	427	222	4.64E-01	0.04	0.05	0.95
Muscle_Skeletal	ENSG00000116459.6	472490	564	276	4.65E-01	-0.01	0.02	0.95
Colon_Sigmoid	ENSG00000233337.1	483868	233	107	4.65E-01	-0.07	0.10	0.95
Pancreas	ENSG00000171385.5	-67773	248	108	4.65E-01	0.04	0.05	0.95
Breast_Mammary_Tissue	ENSG00000273483.1	-597059	290	148	4.66E-01	-0.06	0.09	0.95
Spleen	ENSG00000231437.3	-68388	162	55	4.67E-01	-0.08	0.12	0.95
Vagina	ENSG00000227811.2	181541	115	59	4.67E-01	-0.12	0.16	0.95
Adipose_Visceral_Omentum	ENSG00000121933.13	357420	355	172	4.67E-01	-0.02	0.03	0.95
Brain_Hippocampus	ENSG00000143110.7	447590	123	63	4.67E-01	0.05	0.06	0.95
Brain_Spinal_cord_cervical_c-1	ENSG00000121933.13	357420	91	49	4.69E-01	0.07	0.10	0.95
Liver	ENSG00000162777.12	716847	175	88	4.69E-01	-0.08	0.11	0.95
Lung	ENSG00000225075.1	-775239	427	222	4.70E-01	0.05	0.06	0.95
Colon_Transverse	ENSG00000116455.9	472519	274	119	4.70E-01	0.03	0.04	0.95
Spleen	ENSG00000231246.1	-439146	162	55	4.72E-01	0.09	0.13	0.95
Colon_Transverse	ENSG00000173947.9	575094	274	119	4.72E-01	-0.03	0.04	0.95
Muscle_Skeletal	ENSG00000171385.5	-67773	564	276	4.73E-01	-0.03	0.04	0.95
Skin_Not_Sun_Exposed_Suprapubic	ENSG00000273483.1	-597059	387	200	4.73E-01	0.06	0.08	0.95

Colon_Sigmoid	ENSG00000273483.1	-597059	233	107	4.73E-01	0.04	0.06	0.95
Brain_Cerebellum	ENSG00000184599.9	-799037	173	96	4.74E-01	0.07	0.10	0.95
Brain_Nucleus_accumbens_basal_ganglia	ENSG00000156171.10	781166	147	77	4.74E-01	-0.08	0.11	0.95
Brain_Hippocampus	ENSG00000227811.2	181541	123	63	4.76E-01	-0.09	0.13	0.95
Cells_Transformed_fibroblasts	ENSG00000273483.1	-597059	343	175	4.77E-01	-0.05	0.07	0.95
Esophagus_Gastroesophageal_Junction	ENSG00000155367.11	-794095	244	110	4.77E-01	-0.05	0.07	0.95
Skin_Sun_Exposed_Lower_leg	ENSG00000155366.12	-786052	473	233	4.78E-01	0.02	0.03	0.95
Small_Intestine_Terminal_Ileum	ENSG00000232811.1	977915	137	54	4.78E-01	-0.09	0.12	0.95
Prostate	ENSG00000225075.1	-775239	152	75	4.78E-01	-0.06	0.09	0.95
Spleen	ENSG00000155363.14	-751759	162	55	4.78E-01	-0.05	0.07	0.95
Brain_Spinal_cord_cervical_c-1	ENSG00000273483.1	-597059	91	49	4.78E-01	-0.11	0.15	0.95
Small_Intestine_Terminal_Ileum	ENSG00000143079.10	-474799	137	54	4.79E-01	-0.05	0.07	0.95
Liver	ENSG00000155367.11	-794095	175	88	4.80E-01	0.07	0.11	0.95
Cells_Transformed_fibroblasts	ENSG00000156171.10	781166	343	175	4.80E-01	0.02	0.03	0.95
Colon_Transverse	ENSG00000261654.1	979029	274	119	4.82E-01	-0.06	0.08	0.95
Artery_Aorta	ENSG00000085465.11	493605	299	152	4.82E-01	-0.03	0.04	0.95
Brain_Hippocampus	ENSG00000116455.9	472519	123	63	4.82E-01	-0.06	0.08	0.95
Artery_Aorta	ENSG00000116459.6	472490	299	152	4.84E-01	0.02	0.03	0.95
Liver	ENSG00000007341.14	-699443	175	88	4.84E-01	-0.09	0.13	0.95
Brain_Cerebellar_Hemisphere	ENSG00000162777.12	716847	136	74	4.84E-01	0.07	0.10	0.95
Vagina	ENSG00000064886.9	720611	115	59	4.84E-01	-0.09	0.13	0.95
Spleen	ENSG00000225075.1	-775239	162	55	4.85E-01	0.08	0.12	0.95
Brain_Hippocampus	ENSG00000156171.10	781166	123	63	4.85E-01	-0.06	0.09	0.95
Brain_Spinal_cord_cervical_c-1	ENSG00000197852.8	168128	91	49	4.85E-01	-0.11	0.16	0.95
Brain_Substantia_nigra	ENSG00000197852.8	168128	88	49	4.86E-01	0.09	0.13	0.95
Brain_Frontal_Cortex_BA9	ENSG00000173947.9	575094	129	73	4.86E-01	0.06	0.09	0.95
Esophagus_Gastroesophageal_Junction	ENSG00000232811.1	977915	244	110	4.87E-01	0.08	0.11	0.95
Muscle_Skeletal	ENSG00000064886.9	720611	564	276	4.88E-01	0.03	0.05	0.95
Stomach	ENSG00000116473.10	379164	262	107	4.89E-01	-0.03	0.04	0.95
Brain_Frontal_Cortex_BA9	ENSG00000215866.3	-929261	129	73	4.89E-01	0.11	0.15	0.95
Colon_Transverse	ENSG00000064703.7	166137	274	119	4.89E-01	0.04	0.06	0.95
Adipose_Subcutaneous	ENSG00000155367.11	-794095	442	214	4.90E-01	0.03	0.04	0.95
Nerve_Tibial	ENSG00000156171.10	781166	414	202	4.91E-01	0.04	0.05	0.95
Vagina	ENSG00000134245.13	-545159	115	59	4.91E-01	0.07	0.11	0.95
Esophagus_Muscularis	ENSG00000143110.7	447590	370	188	4.93E-01	-0.03	0.04	0.95
Cells_Transformed_fibroblasts	ENSG00000233337.1	483868	343	175	4.94E-01	-0.04	0.05	0.95
Lung	ENSG00000231246.1	-439146	427	222	4.94E-01	-0.04	0.06	0.95
Pituitary	ENSG00000121933.13	357420	183	95	4.95E-01	0.05	0.07	0.95
Minor_Salivary_Gland	ENSG00000238975.1	-731206	97	49	4.95E-01	-0.10	0.14	0.95
Brain_Cerebellar_Hemisphere	ENSG00000232811.1	977915	136	74	4.96E-01	0.09	0.14	0.95
Vagina	ENSG00000085465.11	493605	115	59	4.96E-01	0.07	0.11	0.95
Adipose_Subcutaneous	ENSG00000273010.1	958080	442	214	4.97E-01	-0.04	0.07	0.95
Colon_Sigmoid	ENSG00000197852.8	168128	233	107	4.97E-01	0.05	0.07	0.95
Brain_Cortex	ENSG00000162777.12	716847	158	79	4.97E-01	-0.08	0.11	0.95
Adrenal_Gland	ENSG00000116455.9	472519	190	96	4.97E-01	0.04	0.06	0.95
Brain_Frontal_Cortex_BA9	ENSG00000232811.1	977915	129	73	4.99E-01	-0.09	0.13	0.95
Esophagus_Mucosa	ENSG00000121931.11	968421	407	208	4.99E-01	-0.03	0.05	0.95

Stomach	ENSG00000155367.11	-794095	262	107	5.01E-01	0.06	0.09	0.95
Brain_Cortex	ENSG00000231346.1	312659	158	79	5.02E-01	-0.09	0.14	0.95
Thyroid	ENSG00000134245.13	-545159	446	224	5.03E-01	-0.03	0.04	0.95
Brain_Substantia_nigra	ENSG00000273010.1	958080	88	49	5.04E-01	-0.11	0.16	0.95
Uterus	ENSG00000243960.1	482557	111	63	5.04E-01	-0.09	0.14	0.95
Minor_Salivary_Gland	ENSG00000143079.10	-474799	97	49	5.05E-01	-0.03	0.05	0.95
Colon_Sigmoid	ENSG00000155367.11	-794095	233	107	5.06E-01	0.04	0.07	0.95
Muscle_Skeletal	ENSG00000156171.10	781166	564	276	5.06E-01	-0.03	0.05	0.95
Pituitary	ENSG00000155366.12	-786052	183	95	5.06E-01	0.04	0.05	0.95
Pituitary	ENSG00000225075.1	-775239	183	95	5.07E-01	-0.08	0.13	0.95
Uterus	ENSG00000143079.10	-474799	111	63	5.07E-01	-0.05	0.07	0.95
Vagina	ENSG00000243960.1	482557	115	59	5.07E-01	0.09	0.14	0.95
Nerve_Tibial	ENSG00000227811.2	181541	414	202	5.07E-01	0.05	0.08	0.95
Pancreas	ENSG00000134255.9	781156	248	108	5.08E-01	-0.05	0.07	0.95
Pancreas	ENSG00000085465.11	493605	248	108	5.09E-01	0.05	0.07	0.95
Cells_EBV-transformed_lymphocytes	ENSG00000156171.10	781166	130	68	5.10E-01	0.04	0.06	0.95
Brain_Spinal_cord_cervical_c-1	ENSG00000243960.1	482557	91	49	5.12E-01	-0.12	0.18	0.95
Esophagus_Gastroesophageal_Junction	ENSG00000171385.5	-67773	244	110	5.13E-01	-0.03	0.04	0.95
Brain_Cerebellum	ENSG00000064886.9	720611	173	96	5.14E-01	-0.06	0.10	0.95
Brain_Caudate_basal_ganglia	ENSG00000162777.12	716847	160	88	5.14E-01	0.06	0.09	0.95
Cells_EBV-transformed_lymphocytes	ENSG00000121931.11	968421	130	68	5.15E-01	-0.06	0.09	0.95
Brain_Amygdala	ENSG00000173947.9	575094	100	47	5.15E-01	0.07	0.10	0.95
Cells_EBV-transformed_lymphocytes	ENSG00000134255.9	781156	130	68	5.16E-01	0.06	0.10	0.95
Cells_Transformed_fibroblasts	ENSG00000085465.11	493605	343	175	5.16E-01	0.02	0.03	0.95
Brain_Anterior_cingulate_cortex_BA24	ENSG00000162777.12	716847	121	65	5.17E-01	0.07	0.10	0.95
Brain_Anterior_cingulate_cortex_BA24	ENSG00000155366.12	-786052	121	65	5.17E-01	-0.02	0.04	0.95
Lung	ENSG00000116473.10	379164	427	222	5.17E-01	0.02	0.03	0.95
Brain_Cerebellum	ENSG00000237556.1	12046	173	96	5.17E-01	-0.08	0.12	0.95
Brain_Frontal_Cortex_BA9	ENSG00000143079.10	-474799	129	73	5.18E-01	0.05	0.08	0.95
Brain_Frontal_Cortex_BA9	ENSG00000243960.1	482557	129	73	5.18E-01	0.08	0.13	0.95
Small_Intestine_Terminal_Ileum	ENSG00000134255.9	781156	137	54	5.19E-01	-0.03	0.05	0.95
Testis	ENSG00000155366.12	-786052	259	128	5.19E-01	-0.02	0.03	0.95
Brain_Frontal_Cortex_BA9	ENSG00000155367.11	-794095	129	73	5.19E-01	0.05	0.07	0.95
Testis	ENSG00000085465.11	493605	259	128	5.20E-01	0.03	0.04	0.95
Brain_Amygdala	ENSG00000064886.9	720611	100	47	5.21E-01	-0.08	0.13	0.95
Artery_Aorta	ENSG00000232811.1	977915	299	152	5.22E-01	-0.06	0.09	0.95
Spleen	ENSG00000121933.13	357420	162	55	5.22E-01	0.06	0.10	0.95
Artery_Coronary	ENSG00000162777.12	716847	173	81	5.22E-01	-0.03	0.05	0.95
Artery_Coronary	ENSG00000134245.13	-545159	173	81	5.22E-01	0.04	0.07	0.95
Prostate	ENSG00000121933.13	357420	152	75	5.23E-01	-0.05	0.07	0.95
Brain_Cerebellum	ENSG00000273010.1	958080	173	96	5.23E-01	-0.07	0.12	0.95
Heart_Left_Ventricle	ENSG00000064886.9	720611	303	154	5.24E-01	0.05	0.07	0.95
Spleen	ENSG00000173947.9	575094	162	55	5.24E-01	-0.10	0.16	0.95
Brain_Putamen_basal_ganglia	ENSG00000260948.1	488314	124	71	5.24E-01	-0.09	0.13	0.95
Spleen	ENSG00000215866.3	-929261	162	55	5.24E-01	-0.09	0.14	0.95
Ovary	ENSG00000155366.12	-786052	133	71	5.24E-01	-0.05	0.08	0.95
Whole_Blood	ENSG00000232811.1	977915	407	195	5.25E-01	0.03	0.05	0.95

Small_Intestine_Terminal_Ileum	ENSG00000134245.13	-545159	137	54	5.26E-01	-0.05	0.08	0.95
Vagina	ENSG00000007341.14	-699443	115	59	5.27E-01	-0.07	0.11	0.95
Brain_Cortex	ENSG00000155366.12	-786052	158	79	5.28E-01	-0.03	0.04	0.95
Brain_Putamen_basal_ganglia	ENSG00000143110.7	447590	124	71	5.28E-01	-0.06	0.09	0.95
Brain_Nucleus_accumbens_basal_ganglia	ENSG00000134255.9	781156	147	77	5.28E-01	0.03	0.06	0.95
Cells_EBV-transformed_lymphocytes	ENSG00000273483.1	-597059	130	68	5.30E-01	-0.08	0.12	0.95
Cells_EBV-transformed_lymphocytes	ENSG00000116455.9	472519	130	68	5.30E-01	0.03	0.05	0.95
Esophagus_Gastroesophageal_Junction	ENSG00000064886.9	720611	244	110	5.30E-01	-0.04	0.07	0.95
Esophagus_Mucosa	ENSG00000116473.10	379164	407	208	5.32E-01	-0.02	0.03	0.95
Skin_Not_Sun_Exposed_Suprapubic	ENSG00000215866.3	-929261	387	200	5.32E-01	-0.05	0.08	0.95
Brain_Spinal_cord_cervical_c-1	ENSG00000155366.12	-786052	91	49	5.33E-01	0.06	0.09	0.95
Pancreas	ENSG00000260948.1	488314	248	108	5.33E-01	-0.06	0.09	0.95
Muscle_Skeletal	ENSG00000273483.1	-597059	564	276	5.33E-01	0.03	0.05	0.95
Cells_Transformed_fibroblasts	ENSG00000232811.1	977915	343	175	5.33E-01	-0.03	0.05	0.95
Whole_Blood	ENSG00000064703.7	166137	407	195	5.33E-01	0.03	0.05	0.95
Prostate	ENSG00000121931.11	968421	152	75	5.34E-01	0.07	0.10	0.95
Heart_Atrial_Appendage	ENSG00000121933.13	357420	297	147	5.35E-01	0.03	0.04	0.95
Brain_Nucleus_accumbens_basal_ganglia	ENSG00000155367.11	-794095	147	77	5.36E-01	0.05	0.09	0.95
Brain_Hippocampus	ENSG00000085465.11	493605	123	63	5.36E-01	-0.05	0.08	0.95
Nerve_Tibial	ENSG00000162777.12	716847	414	202	5.37E-01	-0.02	0.04	0.95
Spleen	ENSG00000155366.12	-786052	162	55	5.37E-01	0.04	0.07	0.95
Lung	ENSG00000134245.13	-545159	427	222	5.37E-01	-0.02	0.04	0.95
Esophagus_Muscularis	ENSG00000260948.1	488314	370	188	5.37E-01	0.04	0.07	0.95
Brain_Cerebellum	ENSG00000155367.11	-794095	173	96	5.38E-01	0.05	0.08	0.95
Colon_Sigmoid	ENSG00000215867.4	271838	233	107	5.38E-01	-0.06	0.10	0.95
Pituitary	ENSG00000273483.1	-597059	183	95	5.40E-01	0.05	0.08	0.96
Brain_Hypothalamus	ENSG00000007341.14	-699443	121	61	5.41E-01	-0.07	0.12	0.96
Breast_Mammary_Tissue	ENSG00000225075.1	-775239	290	148	5.42E-01	0.06	0.09	0.96
Brain_Spinal_cord_cervical_c-1	ENSG00000233337.1	483868	91	49	5.43E-01	-0.11	0.18	0.96
Esophagus_Mucosa	ENSG00000116489.8	-698415	407	208	5.43E-01	-0.02	0.03	0.96
Brain_Substantia_nigra	ENSG00000231437.3	-68388	88	49	5.44E-01	0.05	0.08	0.96
Brain_Anterior_cingulate_cortex_BA24	ENSG00000007341.14	-699443	121	65	5.44E-01	-0.08	0.13	0.96
Brain_Spinal_cord_cervical_c-1	ENSG00000085465.11	493605	91	49	5.44E-01	0.05	0.08	0.96
Esophagus_Mucosa	ENSG00000197852.8	168128	407	208	5.44E-01	-0.03	0.05	0.96
Ovary	ENSG00000134255.9	781156	133	71	5.46E-01	0.06	0.10	0.96
Pituitary	ENSG00000184599.9	-799037	183	95	5.46E-01	0.07	0.12	0.96
Skin_Not_Sun_Exposed_Suprapubic	ENSG00000273010.1	958080	387	200	5.47E-01	0.05	0.08	0.96
Vagina	ENSG00000197852.8	168128	115	59	5.47E-01	-0.05	0.09	0.96
Colon_Sigmoid	ENSG00000143110.7	447590	233	107	5.48E-01	-0.04	0.06	0.96
Brain_Cerebellar_Hemisphere	ENSG00000155363.14	-751759	136	74	5.49E-01	-0.04	0.07	0.96
Artery_Coronary	ENSG00000231346.1	312659	173	81	5.50E-01	-0.05	0.08	0.96
Spleen	ENSG00000143110.7	447590	162	55	5.50E-01	-0.05	0.09	0.96
Artery_Aorta	ENSG00000227811.2	181541	299	152	5.51E-01	-0.06	0.10	0.96
Brain_Caudate_basal_ganglia	ENSG00000197852.8	168128	160	88	5.52E-01	-0.06	0.10	0.96
Brain_Caudate_basal_ganglia	ENSG00000134255.9	781156	160	88	5.52E-01	0.04	0.07	0.96
Brain_Amygdala	ENSG00000273010.1	958080	100	47	5.53E-01	0.11	0.18	0.96

Esophagus_Muscularis	ENSG00000273483.1	-597059	370	188	5.53E-01	-0.03	0.05	0.96
Brain_Spinal_cord_cervical_c-1	ENSG00000116473.10	379164	91	49	5.53E-01	-0.04	0.06	0.96
Heart_Left_Ventricle	ENSG00000155363.14	-751759	303	154	5.53E-01	-0.02	0.03	0.96
Brain_Hippocampus	ENSG00000134255.9	781156	123	63	5.55E-01	0.04	0.07	0.96
Brain_Hippocampus	ENSG00000116459.6	472490	123	63	5.55E-01	0.03	0.05	0.96
Brain_Hippocampus	ENSG00000273010.1	958080	123	63	5.55E-01	-0.09	0.15	0.96
Stomach	ENSG00000121933.13	357420	262	107	5.57E-01	-0.04	0.06	0.96
Brain_Hippocampus	ENSG00000260948.1	488314	123	63	5.59E-01	0.08	0.13	0.96
Muscle_Skeletal	ENSG00000116473.10	379164	564	276	5.59E-01	-0.01	0.02	0.96
Colon_Sigmoid	ENSG00000260948.1	488314	233	107	5.62E-01	0.05	0.08	0.96
Heart_Atrial_Appendage	ENSG00000116473.10	379164	297	147	5.63E-01	-0.03	0.05	0.96
Small_Intestine_Terminal_Ileum	ENSG00000238975.1	-731206	137	54	5.63E-01	0.07	0.13	0.96
Heart_Atrial_Appendage	ENSG00000134245.13	-545159	297	147	5.65E-01	0.04	0.07	0.96
Brain_Cerebellar_Hemisphere	ENSG00000116473.10	379164	136	74	5.66E-01	-0.03	0.06	0.96
Brain_Cerebellar_Hemisphere	ENSG00000184599.9	-799037	136	74	5.66E-01	-0.06	0.10	0.96
Whole_Blood	ENSG00000155366.12	-786052	407	195	5.66E-01	0.01	0.03	0.96
Muscle_Skeletal	ENSG00000227811.2	181541	564	276	5.68E-01	-0.03	0.06	0.96
Thyroid	ENSG00000134216.14	630520	446	224	5.69E-01	0.03	0.06	0.96
Prostate	ENSG00000155367.11	-794095	152	75	5.70E-01	-0.06	0.10	0.96
Pancreas	ENSG00000121931.11	968421	248	108	5.70E-01	-0.04	0.08	0.96
Heart_Left_Ventricle	ENSG00000225075.1	-775239	303	154	5.70E-01	0.05	0.10	0.96
Adipose_Visceral_Omentum	ENSG00000261654.1	979029	355	172	5.72E-01	0.04	0.06	0.96
Thyroid	ENSG00000171385.5	-67773	446	224	5.72E-01	-0.02	0.04	0.96
Ovary	ENSG00000227811.2	181541	133	71	5.73E-01	-0.06	0.11	0.96
Breast_Mammary_Tissue	ENSG00000261654.1	979029	290	148	5.73E-01	-0.04	0.07	0.96
Thyroid	ENSG00000231246.1	-439146	446	224	5.74E-01	0.03	0.06	0.96
Whole_Blood	ENSG00000171385.5	-67773	407	195	5.74E-01	0.04	0.06	0.96
Artery_Aorta	ENSG00000064703.7	166137	299	152	5.76E-01	0.02	0.03	0.96
Adrenal_Gland	ENSG00000231346.1	312659	190	96	5.77E-01	0.04	0.07	0.96
Brain_Substantia_nigra	ENSG00000224167.1	-955558	88	49	5.78E-01	-0.08	0.14	0.96
Whole_Blood	ENSG00000215867.4	271838	407	195	5.78E-01	-0.04	0.07	0.96
Pituitary	ENSG00000243960.1	482557	183	95	5.78E-01	0.08	0.14	0.96
Small_Intestine_Terminal_Ileum	ENSG00000121931.11	968421	137	54	5.79E-01	0.06	0.11	0.96
Heart_Left_Ventricle	ENSG00000143079.10	-474799	303	154	5.83E-01	0.02	0.04	0.96
Muscle_Skeletal	ENSG00000233337.1	483868	564	276	5.83E-01	-0.03	0.06	0.96
Brain_Hippocampus	ENSG00000116489.8	-698415	123	63	5.84E-01	0.05	0.08	0.96
Vagina	ENSG00000231246.1	-439146	115	59	5.85E-01	-0.05	0.10	0.96
Colon_Transverse	ENSG00000143079.10	-474799	274	119	5.85E-01	0.02	0.04	0.96
Skin_Not_Sun_Exposed_Suprapubic	ENSG00000156171.10	781166	387	200	5.85E-01	-0.02	0.04	0.96
Heart_Atrial_Appendage	ENSG00000085465.11	493605	297	147	5.86E-01	0.02	0.04	0.96
Stomach	ENSG00000232811.1	977915	262	107	5.86E-01	-0.05	0.08	0.96
Artery_Aorta	ENSG00000197852.8	168128	299	152	5.86E-01	0.02	0.04	0.96
Adipose_Subcutaneous	ENSG00000155363.14	-751759	442	214	5.87E-01	-0.02	0.03	0.96
Brain_Caudate_basal_ganglia	ENSG00000173947.9	575094	160	88	5.88E-01	0.03	0.06	0.96
Skin_Not_Sun_Exposed_Suprapubic	ENSG00000121931.11	968421	387	200	5.88E-01	0.03	0.05	0.96
Uterus	ENSG00000197852.8	168128	111	63	5.88E-01	0.05	0.09	0.96
Nerve_Tibial	ENSG00000215866.3	-929261	414	202	5.89E-01	-0.03	0.06	0.96

Nerve_Tibial	ENSG00000273010.1	958080	414	202	5.89E-01	-0.04	0.07	0.96
Artery_Tibial	ENSG00000173947.9	575094	441	219	5.92E-01	-0.03	0.05	0.96
Skin_Sun_Exposed_Lower_leg	ENSG00000231346.1	312659	473	233	5.94E-01	-0.03	0.05	0.96
Brain_Cerebellar_Hemisphere	ENSG00000261654.1	979029	136	74	5.94E-01	-0.06	0.11	0.96
Adrenal_Gland	ENSG00000155363.14	-751759	190	96	5.94E-01	0.03	0.06	0.96
Whole_Blood	ENSG00000162777.12	716847	407	195	5.94E-01	0.01	0.03	0.96
Whole_Blood	ENSG00000143079.10	-474799	407	195	5.94E-01	-0.02	0.04	0.96
Brain_Putamen_basal_ganglia	ENSG00000273010.1	958080	124	71	5.95E-01	0.07	0.14	0.96
Brain_Anterior_cingulate_cortex_BA24	ENSG00000231346.1	312659	121	65	5.95E-01	-0.07	0.13	0.96
Colon_Transverse	ENSG00000121931.11	968421	274	119	5.96E-01	-0.04	0.07	0.96
Esophagus_Gastroesophageal_Junction	ENSG00000116455.9	472519	244	110	5.96E-01	-0.03	0.05	0.96
Esophagus_Muscularis	ENSG00000116489.8	-698415	370	188	5.96E-01	-0.02	0.04	0.96
Stomach	ENSG00000155366.12	-786052	262	107	5.98E-01	-0.02	0.03	0.96
Brain_Cerebellum	ENSG00000173947.9	575094	173	96	5.98E-01	0.05	0.09	0.96
Muscle_Skeletal	ENSG00000232811.1	977915	564	276	5.99E-01	-0.03	0.06	0.96
Heart_Atrial_Appendage	ENSG00000116459.6	472490	297	147	5.99E-01	0.01	0.02	0.96
Brain_Spinal_cord_cervical_c-1	ENSG00000155363.14	-751759	91	49	5.99E-01	-0.05	0.09	0.96
Esophagus_Muscularis	ENSG00000155366.12	-786052	370	188	6.01E-01	-0.02	0.04	0.96
Brain_Substantia_nigra	ENSG00000260948.1	488314	88	49	6.02E-01	0.09	0.16	0.96
Adipose_Visceral_Omentum	ENSG00000273483.1	-597059	355	172	6.03E-01	0.04	0.07	0.96
Esophagus_Muscularis	ENSG00000233337.1	483868	370	188	6.03E-01	0.04	0.08	0.96
Skin_Not_Sun_Exposed_Suprapubic	ENSG00000233337.1	483868	387	200	6.03E-01	0.03	0.06	0.96
Adipose_Subcutaneous	ENSG00000085465.11	493605	442	214	6.04E-01	0.02	0.03	0.96
Esophagus_Mucosa	ENSG00000231346.1	312659	407	208	6.04E-01	0.03	0.05	0.96
Brain_Nucleus_accumbens_basal_ganglia	ENSG00000116459.6	472490	147	77	6.04E-01	-0.03	0.07	0.96
Brain_Hippocampus	ENSG00000007341.14	-699443	123	63	6.05E-01	-0.06	0.12	0.96
Brain_Hippocampus	ENSG00000121931.11	968421	123	63	6.06E-01	0.06	0.12	0.96
Spleen	ENSG00000224167.1	-955558	162	55	6.06E-01	0.07	0.13	0.96
Breast_Mammary_Tissue	ENSG00000155363.14	-751759	290	148	6.06E-01	0.02	0.03	0.96
Small_Intestine_Terminal_Ileum	ENSG00000064703.7	166137	137	54	6.07E-01	0.04	0.08	0.96
Brain_Putamen_basal_ganglia	ENSG00000116455.9	472519	124	71	6.08E-01	-0.06	0.11	0.96
Thyroid	ENSG00000121933.13	357420	446	224	6.08E-01	-0.02	0.03	0.96
Heart_Left_Ventricle	ENSG00000064703.7	166137	303	154	6.08E-01	0.02	0.05	0.96
Brain_Caudate_basal_ganglia	ENSG00000215866.3	-929261	160	88	6.10E-01	0.06	0.12	0.96
Vagina	ENSG00000116473.10	379164	115	59	6.10E-01	-0.04	0.08	0.96
Heart_Left_Ventricle	ENSG00000173947.9	575094	303	154	6.11E-01	-0.02	0.04	0.96
Brain_Spinal_cord_cervical_c-1	ENSG00000273010.1	958080	91	49	6.11E-01	-0.10	0.20	0.96
Nerve_Tibial	ENSG00000238975.1	-731206	414	202	6.12E-01	0.04	0.07	0.96
Lung	ENSG00000231346.1	312659	427	222	6.12E-01	0.03	0.05	0.96
Stomach	ENSG00000116459.6	472490	262	107	6.13E-01	-0.02	0.03	0.96
Minor_Salivary_Gland	ENSG00000155367.11	-794095	97	49	6.13E-01	-0.06	0.11	0.96
Brain_Cerebellum	ENSG00000134245.13	-545159	173	96	6.13E-01	0.05	0.09	0.96
Prostate	ENSG00000085465.11	493605	152	75	6.14E-01	0.04	0.08	0.96
Ovary	ENSG00000173947.9	575094	133	71	6.15E-01	0.04	0.09	0.96
Ovary	ENSG00000233337.1	483868	133	71	6.15E-01	-0.05	0.11	0.96
Lung	ENSG00000116459.6	472490	427	222	6.15E-01	0.01	0.02	0.96
Adipose_Subcutaneous	ENSG00000121931.11	968421	442	214	6.15E-01	-0.02	0.04	0.96

Nerve_Tibial	ENSG00000243960.1	482557	414	202	6.15E-01	0.04	0.08	0.96
Brain_Cerebellar_Hemisphere	ENSG00000231437.3	-68388	136	74	6.16E-01	0.04	0.09	0.96
Colon_Transverse	ENSG00000243960.1	482557	274	119	6.17E-01	-0.05	0.09	0.96
Esophagus_Gastroesophageal_Junction	ENSG00000156171.10	781166	244	110	6.18E-01	-0.03	0.07	0.96
Nerve_Tibial	ENSG00000134255.9	781156	414	202	6.18E-01	0.02	0.04	0.96
Brain_Putamen_basal_ganglia	ENSG00000134255.9	781156	124	71	6.19E-01	0.03	0.06	0.96
Brain_Frontal_Cortex_BA9	ENSG00000116489.8	-698415	129	73	6.19E-01	0.05	0.09	0.96
Colon_Transverse	ENSG00000155363.14	-751759	274	119	6.19E-01	-0.02	0.05	0.96
Heart_Left_Ventricle	ENSG00000232811.1	977915	303	154	6.19E-01	-0.05	0.10	0.96
Small_Intestine_Terminal_Ileum	ENSG00000227811.2	181541	137	54	6.20E-01	0.06	0.13	0.96
Brain_Hypothalamus	ENSG00000134245.13	-545159	121	61	6.21E-01	-0.05	0.11	0.96
Artery_Tibial	ENSG00000243960.1	482557	441	219	6.21E-01	0.04	0.08	0.96
Brain_Cerebellum	ENSG00000232811.1	977915	173	96	6.22E-01	-0.05	0.10	0.96
Ovary	ENSG00000162777.12	716847	133	71	6.23E-01	-0.06	0.11	0.96
Brain_Nucleus_accumbens_basal_ganglia	ENSG00000232811.1	977915	147	77	6.23E-01	0.06	0.13	0.96
Cells_EBV-transformed_lymphocytes	ENSG00000231346.1	312659	130	68	6.23E-01	0.05	0.09	0.96
Brain_Cerebellar_Hemisphere	ENSG00000085465.11	493605	136	74	6.24E-01	0.03	0.06	0.96
Uterus	ENSG00000232811.1	977915	111	63	6.24E-01	-0.07	0.15	0.96
Artery_Aorta	ENSG00000156171.10	781166	299	152	6.24E-01	-0.03	0.06	0.96
Stomach	ENSG00000227179.2	529563	262	107	6.25E-01	-0.04	0.08	0.96
Minor_Salivary_Gland	ENSG00000116459.6	472490	97	49	6.26E-01	0.03	0.06	0.96
Esophagus_Mucosa	ENSG00000143110.7	447590	407	208	6.27E-01	-0.02	0.04	0.96
Muscle_Skeletal	ENSG00000155366.12	-786052	564	276	6.27E-01	-0.02	0.03	0.96
Ovary	ENSG00000197852.8	168128	133	71	6.27E-01	-0.04	0.08	0.96
Liver	ENSG00000273010.1	958080	175	88	6.27E-01	0.06	0.13	0.96
Testis	ENSG00000273483.1	-597059	259	128	6.28E-01	-0.03	0.06	0.96
Esophagus_Mucosa	ENSG00000134255.9	781156	407	208	6.28E-01	-0.02	0.04	0.96
Esophagus_Mucosa	ENSG00000155367.11	-794095	407	208	6.28E-01	-0.02	0.04	0.96
Prostate	ENSG00000064703.7	166137	152	75	6.28E-01	0.03	0.07	0.96
Spleen	ENSG00000162777.12	716847	162	55	6.29E-01	0.04	0.08	0.96
Vagina	ENSG00000225075.1	-775239	115	59	6.29E-01	0.08	0.16	0.96
Brain_Hypothalamus	ENSG00000116473.10	379164	121	61	6.30E-01	0.02	0.05	0.96
Brain_Anterior_cingulate_cortex_BA24	ENSG00000116489.8	-698415	121	65	6.30E-01	0.04	0.08	0.96
Brain_Substantia_nigra	ENSG00000085465.11	493605	88	49	6.30E-01	0.04	0.08	0.96
Heart_Atrial_Appendage	ENSG00000116455.9	472519	297	147	6.31E-01	-0.02	0.03	0.96
Brain_Cerebellar_Hemisphere	ENSG00000215866.3	-929261	136	74	6.31E-01	-0.06	0.13	0.96
Adrenal_Gland	ENSG00000231246.1	-439146	190	96	6.31E-01	-0.05	0.10	0.96
Brain_Nucleus_accumbens_basal_ganglia	ENSG00000064886.9	720611	147	77	6.33E-01	-0.05	0.10	0.96
Skin_Sun_Exposed_Lower_leg	ENSG00000134245.13	-545159	473	233	6.33E-01	-0.01	0.03	0.96
Cells_Transformed_fibroblasts	ENSG00000134245.13	-545159	343	175	6.34E-01	-0.02	0.05	0.96
Heart_Atrial_Appendage	ENSG00000273010.1	958080	297	147	6.34E-01	-0.04	0.08	0.96
Adrenal_Gland	ENSG00000116459.6	472490	190	96	6.35E-01	-0.02	0.04	0.96
Lung	ENSG00000064703.7	166137	427	222	6.35E-01	0.02	0.04	0.96
Pituitary	ENSG00000215866.3	-929261	183	95	6.38E-01	-0.07	0.14	0.96
Vagina	ENSG00000232811.1	977915	115	59	6.38E-01	-0.07	0.14	0.96
Colon_Sigmoid	ENSG00000085465.11	493605	233	107	6.38E-01	-0.03	0.05	0.96

Brain_Cortex	ENSG00000184599.9	-799037	158	79	6.38E-01	-0.06	0.13	0.96
Brain_Amygdala	ENSG00000064703.7	166137	100	47	6.40E-01	0.07	0.14	0.96
Adrenal_Gland	ENSG00000116489.8	-698415	190	96	6.41E-01	0.02	0.05	0.96
Colon_Sigmoid	ENSG00000231346.1	312659	233	107	6.41E-01	-0.03	0.06	0.96
Adrenal_Gland	ENSG00000225075.1	-775239	190	96	6.43E-01	0.05	0.10	0.96
Artery_Tibial	ENSG00000231437.3	-68388	441	219	6.43E-01	-0.02	0.05	0.96
Muscle_Skeletal	ENSG00000261654.1	979029	564	276	6.43E-01	0.02	0.05	0.96
Brain_Caudate_basal_ganglia	ENSG00000116459.6	472490	160	88	6.44E-01	-0.03	0.06	0.96
Brain_Putamen_basal_ganglia	ENSG00000243960.1	482557	124	71	6.46E-01	-0.07	0.15	0.96
Muscle_Skeletal	ENSG00000155367.11	-794095	564	276	6.48E-01	-0.01	0.02	0.96
Brain_Hippocampus	ENSG00000143079.10	-474799	123	63	6.48E-01	-0.03	0.07	0.96
Thyroid	ENSG00000231346.1	312659	446	224	6.48E-01	-0.02	0.05	0.96
Whole_Blood	ENSG00000273483.1	-597059	407	195	6.49E-01	0.03	0.06	0.96
Brain_Amygdala	ENSG00000116473.10	379164	100	47	6.49E-01	0.03	0.06	0.96
Artery_Tibial	ENSG00000143110.7	447590	441	219	6.50E-01	-0.02	0.03	0.96
Cells_EBV-transformed_lymphocytes	ENSG00000173947.9	575094	130	68	6.50E-01	-0.06	0.12	0.96
Pituitary	ENSG00000116459.6	472490	183	95	6.51E-01	-0.02	0.05	0.96
Brain_Cerebellum	ENSG00000155366.12	-786052	173	96	6.52E-01	0.02	0.04	0.96
Adipose_Subcutaneous	ENSG00000260948.1	488314	442	214	6.52E-01	-0.02	0.05	0.96
Adrenal_Gland	ENSG00000121931.11	968421	190	96	6.52E-01	0.03	0.08	0.96
Colon_Transverse	ENSG00000231437.3	-68388	274	119	6.53E-01	-0.03	0.06	0.96
Brain_Nucleus_accumbens_basal_ganglia	ENSG00000116455.9	472519	147	77	6.54E-01	-0.05	0.11	0.96
Brain_Putamen_basal_ganglia	ENSG00000171385.5	-67773	124	71	6.54E-01	0.04	0.08	0.96
Cells_Transformed_fibroblasts	ENSG00000197852.8	168128	343	175	6.54E-01	0.02	0.05	0.96
Testis	ENSG00000162777.12	716847	259	128	6.54E-01	0.02	0.05	0.96
Cells_Transformed_fibroblasts	ENSG00000162777.12	716847	343	175	6.58E-01	-0.02	0.05	0.96
Adrenal_Gland	ENSG00000260948.1	488314	190	96	6.59E-01	0.04	0.10	0.96
Nerve_Tibial	ENSG00000064703.7	166137	414	202	6.60E-01	-0.02	0.04	0.96
Vagina	ENSG00000121931.11	968421	115	59	6.61E-01	-0.05	0.11	0.96
Minor_Salivary_Gland	ENSG00000064703.7	166137	97	49	6.61E-01	0.05	0.12	0.96
Cells_EBV-transformed_lymphocytes	ENSG00000116473.10	379164	130	68	6.62E-01	0.04	0.08	0.96
Small_Intestine_Terminal_Ileum	ENSG00000143110.7	447590	137	54	6.62E-01	0.05	0.10	0.96
Stomach	ENSG00000225075.1	-775239	262	107	6.64E-01	-0.04	0.09	0.96
Brain_Frontal_Cortex_BA9	ENSG00000116455.9	472519	129	73	6.64E-01	-0.04	0.09	0.96
Brain_Substantia_nigra	ENSG00000134245.13	-545159	88	49	6.65E-01	-0.05	0.12	0.96
Lung	ENSG00000143079.10	-474799	427	222	6.66E-01	0.01	0.03	0.96
Brain_Substantia_nigra	ENSG00000273483.1	-597059	88	49	6.66E-01	0.06	0.15	0.96
Pituitary	ENSG00000064703.7	166137	183	95	6.67E-01	-0.03	0.07	0.96
Artery_Aorta	ENSG00000134255.9	781156	299	152	6.67E-01	0.02	0.04	0.96
Brain_Hypothalamus	ENSG00000116459.6	472490	121	61	6.68E-01	-0.02	0.06	0.96
Pituitary	ENSG00000116489.8	-698415	183	95	6.69E-01	0.02	0.05	0.96
Uterus	ENSG00000231346.1	312659	111	63	6.70E-01	-0.04	0.10	0.96
Artery_Coronary	ENSG00000116489.8	-698415	173	81	6.71E-01	-0.02	0.05	0.96
Heart_Atrial_Appendage	ENSG00000143079.10	-474799	297	147	6.71E-01	0.02	0.04	0.96
Brain_Amygdala	ENSG00000273483.1	-597059	100	47	6.72E-01	0.05	0.13	0.96
Skin_Sun_Exposed_Lower_leg	ENSG00000224167.1	-955558	473	233	6.72E-01	0.03	0.06	0.96
Brain_Cerebellar_Hemisphere	ENSG00000156171.10	781166	136	74	6.73E-01	-0.04	0.10	0.96

Stomach	ENSG00000231246.1	-439146	262	107	6.74E-01	-0.03	0.08	0.96
Brain_Hippocampus	ENSG00000231346.1	312659	123	63	6.74E-01	0.05	0.13	0.96
Brain_Amygdala	ENSG00000116489.8	-698415	100	47	6.74E-01	0.05	0.11	0.96
Esophagus_Muscularis	ENSG00000134255.9	781156	370	188	6.76E-01	0.02	0.05	0.96
Artery_Tibial	ENSG00000116455.9	472519	441	219	6.76E-01	0.01	0.03	0.96
Brain_Hypothalamus	ENSG00000155363.14	-751759	121	61	6.77E-01	-0.03	0.07	0.96
Muscle_Skeletal	ENSG00000143079.10	-474799	564	276	6.77E-01	-0.01	0.03	0.96
Brain_Putamen_basal_ganglia	ENSG00000116473.10	379164	124	71	6.77E-01	0.02	0.06	0.96
Cells_EBV-transformed_lymphocytes	ENSG00000232811.1	977915	130	68	6.77E-01	0.04	0.11	0.96
Brain_Cortex	ENSG00000116455.9	472519	158	79	6.77E-01	-0.05	0.12	0.96
Brain_Anterior_cingulate_cortex_BA24	ENSG00000260948.1	488314	121	65	6.78E-01	0.05	0.12	0.96
Testis	ENSG00000184599.9	-799037	259	128	6.78E-01	0.02	0.05	0.96
Brain_Hypothalamus	ENSG00000215866.3	-929261	121	61	6.78E-01	-0.05	0.12	0.96
Prostate	ENSG00000197852.8	168128	152	75	6.78E-01	0.04	0.10	0.96
Vagina	ENSG00000155363.14	-751759	115	59	6.78E-01	0.03	0.07	0.96
Thyroid	ENSG00000197852.8	168128	446	224	6.79E-01	0.02	0.04	0.96
Prostate	ENSG00000116459.6	472490	152	75	6.79E-01	-0.02	0.05	0.96
Brain_Hypothalamus	ENSG00000143079.10	-474799	121	61	6.79E-01	-0.03	0.07	0.96
Testis	ENSG00000233337.1	483868	259	128	6.80E-01	0.04	0.09	0.96
Brain_Frontal_Cortex_BA9	ENSG00000231346.1	312659	129	73	6.81E-01	-0.06	0.14	0.96
Skin_Sun_Exposed_Lower_leg	ENSG00000116459.6	472490	473	233	6.81E-01	-0.01	0.02	0.96
Brain_Hypothalamus	ENSG00000116489.8	-698415	121	61	6.82E-01	-0.04	0.09	0.96
Brain_Amygdala	ENSG00000116455.9	472519	100	47	6.83E-01	-0.05	0.13	0.96
Prostate	ENSG00000007341.14	-699443	152	75	6.83E-01	0.05	0.12	0.96
Lung	ENSG00000116455.9	472519	427	222	6.84E-01	-0.01	0.03	0.96
Artery_Coronary	ENSG00000155363.14	-751759	173	81	6.84E-01	-0.02	0.04	0.96
Pancreas	ENSG00000162777.12	716847	248	108	6.84E-01	0.02	0.04	0.96
Whole_Blood	ENSG00000260948.1	488314	407	195	6.84E-01	0.02	0.06	0.96
Stomach	ENSG00000134245.13	-545159	262	107	6.84E-01	-0.02	0.06	0.96
Heart_Left_Ventricle	ENSG00000273483.1	-597059	303	154	6.85E-01	0.03	0.06	0.96
Brain_Cortex	ENSG00000227811.2	181541	158	79	6.85E-01	-0.05	0.13	0.96
Stomach	ENSG00000134216.14	630520	262	107	6.86E-01	-0.02	0.06	0.96
Cells_Transformed_fibroblasts	ENSG00000173947.9	575094	343	175	6.86E-01	0.02	0.04	0.96
Esophagus_Muscularis	ENSG00000231346.1	312659	370	188	6.87E-01	0.01	0.03	0.96
Brain_Cerebellar_Hemisphere	ENSG00000064886.9	720611	136	74	6.87E-01	-0.05	0.12	0.96
Brain_Cerebellar_Hemisphere	ENSG00000155366.12	-786052	136	74	6.87E-01	0.02	0.06	0.96
Brain_Amygdala	ENSG00000232811.1	977915	100	47	6.87E-01	0.09	0.21	0.96
Skin_Not_Sun_Exposed_Suprapubic	ENSG00000134255.9	781156	387	200	6.88E-01	-0.02	0.04	0.96
Skin_Not_Sun_Exposed_Suprapubic	ENSG00000231346.1	312659	387	200	6.91E-01	0.02	0.05	0.96
Thyroid	ENSG00000231437.3	-68388	446	224	6.91E-01	0.02	0.05	0.96
Skin_Sun_Exposed_Lower_leg	ENSG00000215866.3	-929261	473	233	6.92E-01	0.02	0.06	0.96
Stomach	ENSG00000173947.9	575094	262	107	6.92E-01	-0.02	0.06	0.96
Adipose_Subcutaneous	ENSG00000184599.9	-799037	442	214	6.93E-01	0.03	0.06	0.96
Nerve_Tibial	ENSG00000231246.1	-439146	414	202	6.93E-01	-0.02	0.06	0.96
Heart_Atrial_Appendage	ENSG00000134255.9	781156	297	147	6.93E-01	0.02	0.05	0.96
Vagina	ENSG00000260948.1	488314	115	59	6.93E-01	-0.05	0.13	0.96
Brain_Spinal_cord_cervical_c-1	ENSG00000064886.9	720611	91	49	6.95E-01	-0.05	0.13	0.96

Cells_EBV-transformed_lymphocytes	ENSG00000155366.12	-786052	130	68	6.95E-01	-0.03	0.08	0.96
Cells_EBV-transformed_lymphocytes	ENSG00000085465.11	493605	130	68	6.96E-01	-0.04	0.10	0.96
Cells_Transformed_fibroblasts	ENSG00000116489.8	-698415	343	175	6.96E-01	0.01	0.02	0.96
Skin_Not_Sun_Exposed_Suprapubic	ENSG00000227811.2	181541	387	200	6.96E-01	0.03	0.08	0.96
Skin_Not_Sun_Exposed_Suprapubic	ENSG00000064703.7	166137	387	200	6.97E-01	0.02	0.05	0.96
Prostate	ENSG00000116489.8	-698415	152	75	6.98E-01	-0.03	0.09	0.96
Brain_Cerebellum	ENSG00000162777.12	716847	173	96	6.98E-01	-0.03	0.08	0.96
Brain_Amygdala	ENSG00000134245.13	-545159	100	47	6.98E-01	0.05	0.13	0.96
Brain_Nucleus_accumbens_basal_ganglia	ENSG00000173947.9	575094	147	77	6.99E-01	0.03	0.09	0.96
Cells_Transformed_fibroblasts	ENSG00000134255.9	781156	343	175	6.99E-01	0.02	0.05	0.96
Liver	ENSG00000121933.13	357420	175	88	7.00E-01	0.03	0.07	0.96
Uterus	ENSG00000156171.10	781166	111	63	7.01E-01	-0.04	0.11	0.96
Artery_Aorta	ENSG00000116455.9	472519	299	152	7.01E-01	-0.01	0.03	0.96
Stomach	ENSG00000171385.5	-67773	262	107	7.02E-01	0.02	0.05	0.96
Heart_Left_Ventricle	ENSG00000231246.1	-439146	303	154	7.03E-01	-0.03	0.08	0.96
Whole_Blood	ENSG00000064886.9	720611	407	195	7.03E-01	0.02	0.06	0.96
Ovary	ENSG00000156171.10	781166	133	71	7.04E-01	-0.03	0.09	0.96
Pituitary	ENSG00000134255.9	781156	183	95	7.05E-01	-0.03	0.07	0.96
Artery_Coronary	ENSG00000155366.12	-786052	173	81	7.05E-01	-0.02	0.05	0.96
Stomach	ENSG00000227811.2	181541	262	107	7.05E-01	0.03	0.07	0.96
Brain_Hippocampus	ENSG00000155366.12	-786052	123	63	7.06E-01	-0.02	0.05	0.96
Muscle_Skeletal	ENSG00000243960.1	482557	564	276	7.06E-01	-0.02	0.07	0.96
Cells_EBV-transformed_lymphocytes	ENSG00000134216.14	630520	130	68	7.07E-01	-0.05	0.13	0.96
Cells_EBV-transformed_lymphocytes	ENSG00000134245.13	-545159	130	68	7.08E-01	-0.04	0.10	0.96
Heart_Atrial_Appendage	ENSG00000231437.3	-68388	297	147	7.09E-01	0.03	0.07	0.96
Thyroid	ENSG00000162777.12	716847	446	224	7.10E-01	0.01	0.04	0.96
Esophagus_Gastroesophageal_Junction	ENSG00000121933.13	357420	244	110	7.10E-01	0.02	0.06	0.96
Muscle_Skeletal	ENSG00000231437.3	-68388	564	276	7.11E-01	-0.02	0.05	0.96
Brain_Hypothalamus	ENSG00000224167.1	-955558	121	61	7.12E-01	0.05	0.14	0.96
Ovary	ENSG00000116489.8	-698415	133	71	7.13E-01	0.03	0.09	0.96
Whole_Blood	ENSG00000227811.2	181541	407	195	7.14E-01	-0.02	0.06	0.96
Adipose_Visceral_Omentum	ENSG00000156171.10	781166	355	172	7.14E-01	-0.02	0.05	0.96
Brain_Cortex	ENSG00000273010.1	958080	158	79	7.15E-01	-0.05	0.13	0.96
Brain_Caudate_basal_ganglia	ENSG00000227811.2	181541	160	88	7.15E-01	0.05	0.13	0.96
Whole_Blood	ENSG00000273010.1	958080	407	195	7.15E-01	-0.03	0.08	0.96
Thyroid	ENSG00000121931.11	968421	446	224	7.15E-01	-0.01	0.04	0.96
Artery_Coronary	ENSG00000231437.3	-68388	173	81	7.15E-01	0.03	0.07	0.96
Artery_Aorta	ENSG00000155366.12	-786052	299	152	7.16E-01	0.01	0.03	0.96
Skin_Not_Sun_Exposed_Suprapubic	ENSG00000261654.1	979029	387	200	7.17E-01	0.02	0.07	0.96
Ovary	ENSG00000143110.7	447590	133	71	7.17E-01	-0.03	0.09	0.96
Adipose_Visceral_Omentum	ENSG00000197852.8	168128	355	172	7.17E-01	0.01	0.03	0.96
Brain_Caudate_basal_ganglia	ENSG00000116489.8	-698415	160	88	7.17E-01	-0.02	0.05	0.96
Uterus	ENSG00000134245.13	-545159	111	63	7.19E-01	0.04	0.12	0.96
Adipose_Subcutaneous	ENSG00000143079.10	-474799	442	214	7.19E-01	0.01	0.03	0.96
Brain_Cerebellar_Hemisphere	ENSG00000233337.1	483868	136	74	7.19E-01	0.04	0.10	0.96
Vagina	ENSG00000156171.10	781166	115	59	7.20E-01	-0.04	0.10	0.96
Whole_Blood	ENSG00000233337.1	483868	407	195	7.21E-01	0.03	0.08	0.96

Brain_Amygdala	ENSG00000155366.12	-786052	100	47	7.21E-01	0.02	0.07	0.96
Adipose_Subcutaneous	ENSG00000232811.1	977915	442	214	7.21E-01	0.03	0.08	0.96
Spleen	ENSG00000134245.13	-545159	162	55	7.22E-01	-0.04	0.10	0.96
Brain_Amygdala	ENSG00000085465.11	493605	100	47	7.22E-01	0.04	0.11	0.96
Uterus	ENSG00000225075.1	-775239	111	63	7.23E-01	-0.05	0.13	0.96
Lung	ENSG00000162777.12	716847	427	222	7.23E-01	-0.01	0.03	0.96
Breast_Mammary_Tissue	ENSG00000116473.10	379164	290	148	7.23E-01	-0.01	0.03	0.96
Testis	ENSG00000064886.9	720611	259	128	7.24E-01	0.03	0.08	0.96
Pituitary	ENSG00000134245.13	-545159	183	95	7.24E-01	0.04	0.11	0.96
Brain_Putamen_basal_ganglia	ENSG00000173947.9	575094	124	71	7.25E-01	0.04	0.11	0.96
Minor_Salivary_Gland	ENSG00000116455.9	472519	97	49	7.25E-01	-0.03	0.08	0.96
Thyroid	ENSG00000085465.11	493605	446	224	7.26E-01	0.01	0.03	0.96
Colon_Transverse	ENSG00000155366.12	-786052	274	119	7.26E-01	-0.01	0.04	0.96
Cells_EBV-transformed_lymphocytes	ENSG00000227811.2	181541	130	68	7.26E-01	0.05	0.14	0.96
Skin_Not_Sun_Exposed_Suprapubic	ENSG00000231437.3	-68388	387	200	7.27E-01	-0.02	0.05	0.96
Esophagus_Gastroesophageal_Junction	ENSG00000260948.1	488314	244	110	7.27E-01	-0.03	0.09	0.96
Brain_Nucleus_accumbens_basal_ganglia	ENSG00000231437.3	-68388	147	77	7.28E-01	0.03	0.08	0.96
Adipose_Visceral_Omentum	ENSG00000173947.9	575094	355	172	7.28E-01	-0.02	0.05	0.96
Brain_Amygdala	ENSG00000260948.1	488314	100	47	7.29E-01	-0.06	0.17	0.96
Prostate	ENSG00000134245.13	-545159	152	75	7.30E-01	0.03	0.09	0.96
Artery_Coronary	ENSG00000007341.14	-699443	173	81	7.31E-01	-0.03	0.09	0.96
Vagina	ENSG00000261654.1	979029	115	59	7.32E-01	-0.04	0.13	0.96
Vagina	ENSG00000273483.1	-597059	115	59	7.32E-01	-0.04	0.11	0.96
Nerve_Tibial	ENSG00000197852.8	168128	414	202	7.33E-01	0.01	0.03	0.96
Prostate	ENSG00000134255.9	781156	152	75	7.33E-01	-0.03	0.07	0.96
Esophagus_Mucosa	ENSG00000155363.14	-751759	407	208	7.34E-01	-0.01	0.03	0.96
Cells_EBV-transformed_lymphocytes	ENSG00000260948.1	488314	130	68	7.34E-01	0.04	0.10	0.96
Testis	ENSG00000225075.1	-775239	259	128	7.36E-01	0.03	0.08	0.96
Brain_Anterior_cingulate_cortex_BA24	ENSG00000116473.10	379164	121	65	7.36E-01	-0.02	0.05	0.96
Esophagus_Muscularis	ENSG00000173947.9	575094	370	188	7.37E-01	0.01	0.04	0.96
Vagina	ENSG00000171385.5	-67773	115	59	7.37E-01	0.04	0.13	0.96
Lung	ENSG00000231437.3	-68388	427	222	7.38E-01	0.02	0.05	0.96
Adipose_Subcutaneous	ENSG00000064703.7	166137	442	214	7.38E-01	-0.01	0.04	0.96
Colon_Sigmoid	ENSG00000116459.6	472490	233	107	7.39E-01	-0.01	0.03	0.96
Uterus	ENSG00000231246.1	-439146	111	63	7.39E-01	0.04	0.11	0.96
Minor_Salivary_Gland	ENSG00000116473.10	379164	97	49	7.39E-01	-0.04	0.10	0.96
Skin_Sun_Exposed_Lower_leg	ENSG00000162777.12	716847	473	233	7.39E-01	-0.01	0.02	0.96
Cells_EBV-transformed_lymphocytes	ENSG00000155367.11	-794095	130	68	7.40E-01	-0.03	0.09	0.96
Colon_Transverse	ENSG00000064886.9	720611	274	119	7.41E-01	-0.02	0.06	0.96
Artery_Tibial	ENSG00000231246.1	-439146	441	219	7.41E-01	-0.02	0.06	0.96
Brain_Caudate_basal_ganglia	ENSG00000116455.9	472519	160	88	7.41E-01	0.03	0.10	0.96
Cells_Transformed_fibroblasts	ENSG00000171385.5	-67773	343	175	7.42E-01	0.01	0.04	0.96
Brain_Putamen_basal_ganglia	ENSG00000184599.9	-799037	124	71	7.42E-01	-0.05	0.15	0.96
Vagina	ENSG00000121933.13	357420	115	59	7.42E-01	-0.03	0.09	0.96
Brain_Substantia_nigra	ENSG00000064886.9	720611	88	49	7.43E-01	0.04	0.12	0.96
Esophagus_Muscularis	ENSG00000261654.1	979029	370	188	7.44E-01	-0.02	0.07	0.96
Skin_Not_Sun_Exposed_Suprapubic	ENSG00000121933.13	357420	387	200	7.44E-01	0.01	0.04	0.96

Ovary	ENSG00000121933.13	357420	133	71	7.44E-01	-0.02	0.07	0.96
Liver	ENSG00000116459.6	472490	175	88	7.45E-01	0.01	0.04	0.96
Lung	ENSG00000203878.7	641323	427	222	7.46E-01	0.02	0.06	0.96
Artery_Coronary	ENSG00000155367.11	-794095	173	81	7.48E-01	0.02	0.06	0.96
Artery_Aorta	ENSG00000233337.1	483868	299	152	7.48E-01	0.03	0.09	0.96
Colon_Transverse	ENSG00000155367.11	-794095	274	119	7.48E-01	0.02	0.07	0.96
Heart_Left_Ventricle	ENSG00000231346.1	312659	303	154	7.51E-01	0.02	0.06	0.96
Muscle_Skeletal	ENSG00000143110.7	447590	564	276	7.51E-01	-0.01	0.04	0.96
Whole_Blood	ENSG00000231437.3	-68388	407	195	7.52E-01	0.02	0.07	0.96
Brain_Cortex	ENSG00000085465.11	493605	158	79	7.53E-01	0.02	0.07	0.96
Artery_Coronary	ENSG00000085465.11	493605	173	81	7.53E-01	-0.02	0.06	0.96
Nerve_Tibial	ENSG00000155363.14	-751759	414	202	7.53E-01	-0.01	0.03	0.96
Whole_Blood	ENSG00000085465.11	493605	407	195	7.54E-01	-0.01	0.03	0.96
Stomach	ENSG00000064886.9	720611	262	107	7.54E-01	-0.02	0.06	0.96
Skin_Sun_Exposed_Lower_leg	ENSG00000260948.1	488314	473	233	7.55E-01	0.01	0.03	0.96
Esophagus_Muscularis	ENSG00000121931.11	968421	370	188	7.58E-01	-0.01	0.05	0.96
Brain_Spinal_cord_cervical_c-1	ENSG00000116489.8	-698415	91	49	7.58E-01	0.03	0.10	0.96
Brain_Hypothalamus	ENSG00000232811.1	977915	121	61	7.58E-01	-0.04	0.14	0.96
Whole_Blood	ENSG00000155367.11	-794095	407	195	7.59E-01	-0.02	0.06	0.96
Cells_EBV-transformed_lymphocytes	ENSG00000162777.12	716847	130	68	7.60E-01	0.03	0.10	0.96
Heart_Left_Ventricle	ENSG00000231437.3	-68388	303	154	7.60E-01	0.02	0.06	0.96
Small_Intestine_Terminal_Ileum	ENSG00000231246.1	-439146	137	54	7.60E-01	-0.04	0.13	0.96
Small_Intestine_Terminal_Ileum	ENSG00000260948.1	488314	137	54	7.61E-01	-0.02	0.08	0.96
Muscle_Skeletal	ENSG00000064703.7	166137	564	276	7.61E-01	-0.01	0.03	0.96
Heart_Left_Ventricle	ENSG00000121931.11	968421	303	154	7.62E-01	-0.01	0.05	0.96
Brain_Spinal_cord_cervical_c-1	ENSG00000116455.9	472519	91	49	7.63E-01	-0.04	0.15	0.96
Ovary	ENSG00000231346.1	312659	133	71	7.64E-01	-0.03	0.09	0.96
Brain_Substantia_nigra	ENSG00000143079.10	-474799	88	49	7.64E-01	0.03	0.10	0.96
Esophagus_Mucosa	ENSG00000162777.12	716847	407	208	7.64E-01	0.01	0.03	0.96
Brain_Hypothalamus	ENSG00000273010.1	958080	121	61	7.65E-01	0.04	0.14	0.96
Heart_Atrial_Appendage	ENSG00000232811.1	977915	297	147	7.65E-01	-0.03	0.09	0.96
Nerve_Tibial	ENSG00000085465.11	493605	414	202	7.66E-01	-0.01	0.03	0.96
Artery_Coronary	ENSG00000232811.1	977915	173	81	7.66E-01	0.03	0.12	0.96
Uterus	ENSG00000064886.9	720611	111	63	7.68E-01	-0.04	0.14	0.96
Vagina	ENSG00000184599.9	-799037	115	59	7.68E-01	-0.04	0.13	0.96
Stomach	ENSG00000134255.9	781156	262	107	7.69E-01	-0.01	0.04	0.96
Artery_Coronary	ENSG00000243960.1	482557	173	81	7.70E-01	-0.04	0.13	0.96
Lung	ENSG00000233337.1	483868	427	222	7.70E-01	-0.02	0.06	0.96
Brain_Putamen_basal_ganglia	ENSG00000121931.11	968421	124	71	7.70E-01	0.03	0.11	0.96
Cells_Transformed_fibroblasts	ENSG00000155367.11	-794095	343	175	7.71E-01	0.01	0.05	0.96
Brain_Nucleus_accumbens_basal_ganglia	ENSG00000273010.1	958080	147	77	7.71E-01	0.04	0.13	0.96
Heart_Atrial_Appendage	ENSG00000231246.1	-439146	297	147	7.72E-01	-0.02	0.07	0.96
Brain_Anterior_cingulate_cortex_BA24	ENSG00000116455.9	472519	121	65	7.74E-01	-0.03	0.10	0.96
Adipose_Visceral_Omentum	ENSG00000085465.11	493605	355	172	7.74E-01	0.01	0.04	0.96
Brain_Cerebellar_Hemisphere	ENSG00000173947.9	575094	136	74	7.74E-01	-0.03	0.10	0.96
Artery_Aorta	ENSG00000162777.12	716847	299	152	7.74E-01	0.01	0.04	0.96
Esophagus_Muscularis	ENSG00000121933.13	357420	370	188	7.75E-01	-0.01	0.04	0.96

Brain_Anterior_cingulate_cortex_BA24	ENSG00000243960.1	482557	121	65	7.75E-01	-0.04	0.14	0.96
Brain_Putamen_basal_ganglia	ENSG00000156171.10	781166	124	71	7.75E-01	-0.04	0.12	0.96
Adrenal_Gland	ENSG00000134255.9	781156	190	96	7.76E-01	0.02	0.06	0.96
Liver	ENSG00000155366.12	-786052	175	88	7.76E-01	0.02	0.06	0.96
Brain_Caudate_basal_ganglia	ENSG00000233337.1	483868	160	88	7.77E-01	0.03	0.11	0.96
Pancreas	ENSG00000064886.9	720611	248	108	7.79E-01	-0.03	0.09	0.96
Skin_Sun_Exposed_Lower_leg	ENSG00000134255.9	781156	473	233	7.79E-01	0.01	0.04	0.96
Brain_Substantia_nigra	ENSG00000007341.14	-699443	88	49	7.79E-01	-0.04	0.14	0.96
Spleen	ENSG00000064886.9	720611	162	55	7.79E-01	-0.04	0.14	0.96
Spleen	ENSG00000134255.9	781156	162	55	7.79E-01	-0.02	0.09	0.96
Esophagus_Muscularis	ENSG00000116459.6	472490	370	188	7.80E-01	0.01	0.02	0.96
Artery_Aorta	ENSG00000260948.1	488314	299	152	7.80E-01	-0.02	0.07	0.96
Brain_Cortex	ENSG00000215866.3	-929261	158	79	7.81E-01	0.04	0.14	0.96
Brain_Cortex	ENSG00000121931.11	968421	158	79	7.81E-01	0.03	0.10	0.96
Colon_Transverse	ENSG00000231246.1	-439146	274	119	7.82E-01	-0.02	0.08	0.96
Colon_Sigmoid	ENSG00000227811.2	181541	233	107	7.82E-01	0.03	0.12	0.96
Brain_Cortex	ENSG00000224167.1	-955558	158	79	7.82E-01	-0.04	0.14	0.96
Nerve_Tibial	ENSG00000116459.6	472490	414	202	7.83E-01	-0.01	0.02	0.96
Brain_Frontal_Cortex_BA9	ENSG00000261654.1	979029	129	73	7.83E-01	0.03	0.11	0.96
Thyroid	ENSG00000243960.1	482557	446	224	7.83E-01	0.02	0.07	0.96
Stomach	ENSG00000116455.9	472519	262	107	7.83E-01	0.01	0.05	0.96
Brain_Substantia_nigra	ENSG00000064703.7	166137	88	49	7.84E-01	0.03	0.12	0.96
Brain_Hypothalamus	ENSG00000197852.8	168128	121	61	7.84E-01	0.03	0.11	0.96
Adrenal_Gland	ENSG00000273010.1	958080	190	96	7.85E-01	-0.03	0.11	0.96
Esophagus_Muscularis	ENSG00000231246.1	-439146	370	188	7.87E-01	-0.02	0.07	0.96
Brain_Cerebellum	ENSG00000085465.11	493605	173	96	7.87E-01	-0.02	0.06	0.96
Nerve_Tibial	ENSG00000184599.9	-799037	414	202	7.87E-01	0.02	0.06	0.96
Artery_Coronary	ENSG00000231246.1	-439146	173	81	7.87E-01	0.03	0.10	0.96
Esophagus_Mucosa	ENSG00000231246.1	-439146	407	208	7.87E-01	-0.02	0.07	0.96
Spleen	ENSG00000085465.11	493605	162	55	7.88E-01	0.03	0.09	0.97
Brain_Caudate_basal_ganglia	ENSG00000243960.1	482557	160	88	7.89E-01	-0.04	0.13	0.97
Artery_Tibial	ENSG00000134245.13	-545159	441	219	7.91E-01	0.01	0.04	0.97
Small_Intestine_Terminal_Ileum	ENSG00000233337.1	483868	137	54	7.91E-01	-0.03	0.11	0.97
Colon_Transverse	ENSG00000225075.1	-775239	274	119	7.93E-01	0.02	0.09	0.97
Lung	ENSG00000007341.14	-699443	427	222	7.94E-01	-0.01	0.06	0.97
Brain_Anterior_cingulate_cortex_BA24	ENSG00000224167.1	-955558	121	65	7.94E-01	0.04	0.14	0.97
Pancreas	ENSG00000155366.12	-786052	248	108	7.94E-01	-0.01	0.04	0.97
Skin_Sun_Exposed_Lower_leg	ENSG00000155367.11	-794095	473	233	7.95E-01	0.01	0.03	0.97
Prostate	ENSG00000231246.1	-439146	152	75	7.95E-01	-0.03	0.10	0.97
Artery_Aorta	ENSG00000143110.7	447590	299	152	7.99E-01	-0.01	0.04	0.97
Adrenal_Gland	ENSG00000162777.12	716847	190	96	8.00E-01	-0.02	0.07	0.97
Artery_Coronary	ENSG00000116459.6	472490	173	81	8.01E-01	-0.01	0.03	0.97
Brain_Caudate_basal_ganglia	ENSG00000260948.1	488314	160	88	8.03E-01	-0.02	0.10	0.97
Spleen	ENSG00000116455.9	472519	162	55	8.04E-01	0.02	0.09	0.97
Prostate	ENSG00000231437.3	-68388	152	75	8.05E-01	0.02	0.08	0.97
Muscle_Skeletal	ENSG00000121931.11	968421	564	276	8.06E-01	0.01	0.04	0.97
Brain_Nucleus_accumbens_basal_ganglia	ENSG00000171385.5	-67773	147	77	8.06E-01	-0.01	0.04	0.97

Brain_Frontal_Cortex_BA9	ENSG00000171385.5	-67773	129	73	8.06E-01	0.01	0.06	0.97
Esophagus_Muscularis	ENSG00000227811.2	181541	370	188	8.08E-01	0.02	0.08	0.97
Ovary	ENSG00000231246.1	-439146	133	71	8.08E-01	0.03	0.10	0.97
Prostate	ENSG00000227811.2	181541	152	75	8.09E-01	0.03	0.12	0.97
Esophagus_Gastroesophageal_Junction	ENSG00000231437.3	-68388	244	110	8.09E-01	-0.02	0.07	0.97
Skin_Sun_Exposed_Lower_leg	ENSG00000155363.14	-751759	473	233	8.09E-01	0.01	0.03	0.97
Testis	ENSG00000261654.1	979029	259	128	8.10E-01	-0.02	0.08	0.97
Lung	ENSG00000273483.1	-597059	427	222	8.10E-01	0.01	0.06	0.97
Brain_Putamen_basal_ganglia	ENSG00000116489.8	-698415	124	71	8.11E-01	0.02	0.08	0.97
Brain_Putamen_basal_ganglia	ENSG00000064703.7	166137	124	71	8.12E-01	0.02	0.10	0.97
Brain_Caudate_basal_ganglia	ENSG00000224167.1	-955558	160	88	8.13E-01	-0.03	0.13	0.97
Heart_Left_Ventricle	ENSG00000243960.1	482557	303	154	8.14E-01	-0.02	0.10	0.97
Brain_Cerebellar_Hemisphere	ENSG00000171385.5	-67773	136	74	8.16E-01	-0.02	0.07	0.97
Skin_Sun_Exposed_Lower_leg	ENSG00000121933.13	357420	473	233	8.17E-01	0.01	0.04	0.97
Pituitary	ENSG00000134216.14	630520	183	95	8.18E-01	-0.02	0.09	0.97
Adrenal_Gland	ENSG00000121933.13	357420	190	96	8.18E-01	0.01	0.06	0.97
Vagina	ENSG00000155367.11	-794095	115	59	8.19E-01	0.02	0.07	0.97
Brain_Anterior_cingulate_cortex_BA24	ENSG00000121931.11	968421	121	65	8.19E-01	0.02	0.10	0.97
Brain_Anterior_cingulate_cortex_BA24	ENSG00000215866.3	-929261	121	65	8.20E-01	-0.03	0.13	0.97
Breast_Mammary_Tissue	ENSG00000121931.11	968421	290	148	8.20E-01	0.01	0.06	0.97
Breast_Mammary_Tissue	ENSG00000155367.11	-794095	290	148	8.21E-01	0.01	0.04	0.97
Whole_Blood	ENSG00000121931.11	968421	407	195	8.21E-01	0.01	0.02	0.97
Heart_Left_Ventricle	ENSG00000233337.1	483868	303	154	8.22E-01	-0.02	0.10	0.97
Muscle_Skeletal	ENSG00000155363.14	-751759	564	276	8.22E-01	-0.01	0.03	0.97
Adipose_Subcutaneous	ENSG00000197852.8	168128	442	214	8.22E-01	0.01	0.04	0.97
Skin_Sun_Exposed_Lower_leg	ENSG00000233337.1	483868	473	233	8.23E-01	0.01	0.05	0.97
Stomach	ENSG00000273483.1	-597059	262	107	8.23E-01	0.02	0.09	0.97
Cells_EBV-transformed_lymphocytes	ENSG00000233337.1	483868	130	68	8.23E-01	0.03	0.12	0.97
Vagina	ENSG00000143110.7	447590	115	59	8.24E-01	0.02	0.08	0.97
Ovary	ENSG00000273483.1	-597059	133	71	8.24E-01	0.02	0.08	0.97
Artery_Coronary	ENSG00000173947.9	575094	173	81	8.24E-01	0.02	0.09	0.97
Brain_Anterior_cingulate_cortex_BA24	ENSG00000156171.10	781166	121	65	8.24E-01	-0.02	0.09	0.97
Artery_Coronary	ENSG00000171385.5	-67773	173	81	8.24E-01	0.02	0.09	0.97
Uterus	ENSG00000121933.13	357420	111	63	8.27E-01	-0.02	0.10	0.98
Brain_Anterior_cingulate_cortex_BA24	ENSG00000143110.7	447590	121	65	8.28E-01	-0.01	0.06	0.98
Brain_Cerebellar_Hemisphere	ENSG00000231346.1	312659	136	74	8.29E-01	-0.03	0.12	0.98
Cells_Transformed_fibroblasts	ENSG00000064703.7	166137	343	175	8.30E-01	-0.01	0.03	0.98
Brain_Cerebellum	ENSG00000121933.13	357420	173	96	8.30E-01	-0.02	0.09	0.98
Prostate	ENSG00000243960.1	482557	152	75	8.31E-01	0.02	0.11	0.98
Prostate	ENSG00000233337.1	483868	152	75	8.32E-01	0.02	0.09	0.98
Small_Intestine_Terminal_Ileum	ENSG00000155366.12	-786052	137	54	8.33E-01	-0.01	0.03	0.98
Lung	ENSG00000273010.1	958080	427	222	8.33E-01	-0.02	0.07	0.98
Breast_Mammary_Tissue	ENSG00000231246.1	-439146	290	148	8.33E-01	0.01	0.06	0.98
Brain_Frontal_Cortex_BA9	ENSG00000155366.12	-786052	129	73	8.33E-01	0.01	0.03	0.98
Thyroid	ENSG00000260948.1	488314	446	224	8.34E-01	0.01	0.05	0.98
Pituitary	ENSG00000085465.11	493605	183	95	8.34E-01	0.02	0.10	0.98
Pancreas	ENSG00000007341.14	-699443	248	108	8.38E-01	0.02	0.08	0.98

Skin_Not_Sun_Exposed_Suprapubic	ENSG00000184599.9	-799037	387	200	8.39E-01	0.01	0.06	0.98
Adipose_Subcutaneous	ENSG00000261654.1	979029	442	214	8.42E-01	0.01	0.07	0.98
Brain_Nucleus_accumbens_basal_ganglia	ENSG00000233337.1	483868	147	77	8.44E-01	-0.03	0.13	0.98
Colon_Transverse	ENSG00000162777.12	716847	274	119	8.46E-01	-0.01	0.03	0.98
Brain_Putamen_basal_ganglia	ENSG00000155366.12	-786052	124	71	8.46E-01	-0.01	0.05	0.98
Cells_EBV-transformed_lymphocytes	ENSG00000215867.4	271838	130	68	8.46E-01	0.02	0.12	0.98
Whole_Blood	ENSG00000134255.9	781156	407	195	8.47E-01	0.00	0.02	0.98
Prostate	ENSG00000215866.3	-929261	152	75	8.47E-01	-0.02	0.11	0.98
Minor_Salivary_Gland	ENSG00000225075.1	-775239	97	49	8.47E-01	-0.03	0.15	0.98
Adipose_Visceral_Omentum	ENSG00000232811.1	977915	355	172	8.47E-01	-0.01	0.07	0.98
Artery_Tibial	ENSG00000085465.11	493605	441	219	8.48E-01	0.01	0.03	0.98
Adipose_Visceral_Omentum	ENSG00000227179.2	529563	355	172	8.48E-01	-0.01	0.06	0.98
Artery_Aorta	ENSG00000173947.9	575094	299	152	8.48E-01	0.01	0.08	0.98
Brain_Hypothalamus	ENSG00000273483.1	-597059	121	61	8.48E-01	-0.02	0.09	0.98
Adipose_Visceral_Omentum	ENSG00000233337.1	483868	355	172	8.48E-01	-0.01	0.07	0.98
Ovary	ENSG00000121931.11	968421	133	71	8.49E-01	-0.02	0.08	0.98
Breast_Mammary_Tissue	ENSG00000184599.9	-799037	290	148	8.50E-01	0.01	0.06	0.98
Liver	ENSG00000134255.9	781156	175	88	8.51E-01	-0.02	0.08	0.98
Brain_Anterior_cingulate_cortex_BA24	ENSG00000231437.3	-68388	121	65	8.51E-01	0.02	0.11	0.98
Pancreas	ENSG00000116459.6	472490	248	108	8.53E-01	0.01	0.05	0.98
Cells_Transformed_fibroblasts	ENSG00000227811.2	181541	343	175	8.54E-01	0.01	0.07	0.98
Brain_Putamen_basal_ganglia	ENSG00000116459.6	472490	124	71	8.55E-01	-0.01	0.07	0.98
Nerve_Tibial	ENSG00000116455.9	472519	414	202	8.55E-01	-0.01	0.03	0.98
Uterus	ENSG00000238975.1	-731206	111	63	8.55E-01	-0.03	0.14	0.98
Brain_Amygdala	ENSG00000007341.14	-699443	100	47	8.57E-01	0.03	0.17	0.99
Liver	ENSG00000233337.1	483868	175	88	8.57E-01	-0.02	0.10	0.99
Esophagus_Muscularis	ENSG00000231437.3	-68388	370	188	8.58E-01	-0.01	0.05	0.99
Skin_Not_Sun_Exposed_Suprapubic	ENSG00000134245.13	-545159	387	200	8.59E-01	-0.01	0.03	0.99
Adrenal_Gland	ENSG00000171385.5	-67773	190	96	8.60E-01	0.01	0.08	0.99
Spleen	ENSG00000261654.1	979029	162	55	8.61E-01	-0.02	0.11	0.99
Brain_Cortex	ENSG00000134255.9	781156	158	79	8.62E-01	0.02	0.09	0.99
Brain_Frontal_Cortex_BA9	ENSG00000156171.10	781166	129	73	8.65E-01	0.01	0.09	0.99
Pituitary	ENSG00000233337.1	483868	183	95	8.65E-01	-0.02	0.11	0.99
Skin_Not_Sun_Exposed_Suprapubic	ENSG00000173947.9	575094	387	200	8.66E-01	0.01	0.06	0.99
Liver	ENSG00000085465.11	493605	175	88	8.66E-01	-0.01	0.07	0.99
Brain_Cortex	ENSG00000064703.7	166137	158	79	8.66E-01	-0.02	0.10	0.99
Brain_Caudate_basal_ganglia	ENSG00000116473.10	379164	160	88	8.67E-01	-0.01	0.04	0.99
Colon_Transverse	ENSG00000231346.1	312659	274	119	8.68E-01	-0.01	0.05	0.99
Pituitary	ENSG00000155363.14	-751759	183	95	8.69E-01	-0.01	0.08	0.99
Artery_Coronary	ENSG00000273483.1	-597059	173	81	8.69E-01	-0.02	0.10	0.99
Brain_Hippocampus	ENSG00000215866.3	-929261	123	63	8.70E-01	-0.02	0.12	0.99
Brain_Cerebellum	ENSG00000116489.8	-698415	173	96	8.71E-01	0.01	0.06	0.99
Brain_Spinal_cord_cervical_c-1	ENSG00000162777.12	716847	91	49	8.71E-01	0.02	0.12	0.99
Lung	ENSG00000116489.8	-698415	427	222	8.73E-01	0.00	0.02	0.99
Adipose_Visceral_Omentum	ENSG00000155366.12	-786052	355	172	8.74E-01	0.01	0.03	0.99
Testis	ENSG00000173947.9	575094	259	128	8.74E-01	0.00	0.03	0.99
Ovary	ENSG00000116459.6	472490	133	71	8.75E-01	-0.01	0.06	0.99

Pituitary	ENSG00000261654.1	979029	183	95	8.78E-01	-0.02	0.13	0.99
Esophagus_Muscularis	ENSG00000085465.11	493605	370	188	8.78E-01	-0.01	0.04	0.99
Prostate	ENSG00000231346.1	312659	152	75	8.79E-01	0.01	0.08	0.99
Brain_Frontal_Cortex_BA9	ENSG00000121931.11	968421	129	73	8.80E-01	-0.01	0.07	0.99
Adrenal_Gland	ENSG00000155367.11	-794095	190	96	8.80E-01	0.01	0.07	0.99
Breast_Mammary_Tissue	ENSG00000134245.13	-545159	290	148	8.80E-01	0.01	0.06	0.99
Whole_Blood	ENSG00000116455.9	472519	407	195	8.80E-01	-0.01	0.04	0.99
Thyroid	ENSG00000184599.9	-799037	446	224	8.82E-01	-0.01	0.05	0.99
Artery_Coronary	ENSG00000227811.2	181541	173	81	8.82E-01	-0.02	0.13	0.99
Brain_Frontal_Cortex_BA9	ENSG00000162777.12	716847	129	73	8.82E-01	-0.02	0.11	0.99
Stomach	ENSG00000156171.10	781166	262	107	8.82E-01	0.01	0.06	0.99
Brain_Hypothalamus	ENSG00000260948.1	488314	121	61	8.83E-01	0.02	0.12	0.99
Small_Intestine_Terminal_Ileum	ENSG00000007341.14	-699443	137	54	8.83E-01	-0.02	0.11	0.99
Heart_Left_Ventricle	ENSG00000197852.8	168128	303	154	8.83E-01	0.01	0.04	0.99
Lung	ENSG00000171385.5	-67773	427	222	8.84E-01	0.01	0.04	0.99
Skin_Not_Sun_Exposed_Suprapubic	ENSG00000007341.14	-699443	387	200	8.84E-01	0.01	0.04	0.99
Brain_Hippocampus	ENSG00000197852.8	168128	123	63	8.84E-01	0.01	0.06	0.99
Brain_Nucleus_accumbens_basal_ganglia	ENSG00000184599.9	-799037	147	77	8.84E-01	0.02	0.12	0.99
Pituitary	ENSG00000260948.1	488314	183	95	8.86E-01	-0.02	0.12	0.99
Adrenal_Gland	ENSG00000064886.9	720611	190	96	8.87E-01	-0.01	0.08	0.99
Spleen	ENSG00000156171.10	781166	162	55	8.87E-01	0.02	0.11	0.99
Brain_Cerebellum	ENSG00000260948.1	488314	173	96	8.88E-01	-0.01	0.06	0.99
Artery_Aorta	ENSG00000231246.1	-439146	299	152	8.88E-01	-0.01	0.08	0.99
Skin_Not_Sun_Exposed_Suprapubic	ENSG00000171385.5	-67773	387	200	8.88E-01	-0.01	0.05	0.99
Heart_Left_Ventricle	ENSG00000007341.14	-699443	303	154	8.88E-01	0.01	0.07	0.99
Artery_Tibial	ENSG00000121933.13	357420	441	219	8.88E-01	-0.01	0.04	0.99
Breast_Mammary_Tissue	ENSG00000260948.1	488314	290	148	8.90E-01	-0.01	0.07	0.99
Brain_Cerebellum	ENSG00000233337.1	483868	173	96	8.90E-01	0.01	0.10	0.99
Minor_Salivary_Gland	ENSG00000231246.1	-439146	97	49	8.90E-01	-0.02	0.12	0.99
Brain_Nucleus_accumbens_basal_ganglia	ENSG00000143079.10	-474799	147	77	8.91E-01	0.01	0.08	0.99
Heart_Left_Ventricle	ENSG00000116459.6	472490	303	154	8.91E-01	0.00	0.02	0.99
Heart_Atrial_Appendage	ENSG00000143110.7	447590	297	147	8.92E-01	0.01	0.04	0.99
Adipose_Subcutaneous	ENSG00000227811.2	181541	442	214	8.93E-01	0.01	0.07	0.99
Adipose_Subcutaneous	ENSG00000231246.1	-439146	442	214	8.94E-01	0.01	0.07	0.99
Brain_Cortex	ENSG00000173947.9	575094	158	79	8.96E-01	-0.01	0.07	0.99
Brain_Cerebellum	ENSG00000116459.6	472490	173	96	8.96E-01	-0.01	0.05	0.99
Heart_Left_Ventricle	ENSG00000116455.9	472519	303	154	8.96E-01	0.00	0.03	0.99
Brain_Cerebellar_Hemisphere	ENSG00000143079.10	-474799	136	74	8.97E-01	-0.01	0.09	0.99
Adrenal_Gland	ENSG00000261654.1	979029	190	96	8.98E-01	-0.01	0.12	0.99
Brain_Anterior_cingulate_cortex_BA24	ENSG00000173947.9	575094	121	65	8.98E-01	0.02	0.12	0.99
Artery_Tibial	ENSG00000116459.6	472490	441	219	8.98E-01	0.00	0.02	0.99
Stomach	ENSG00000203878.7	641323	262	107	9.01E-01	0.01	0.07	0.99
Colon_Sigmoid	ENSG00000143079.10	-474799	233	107	9.01E-01	0.01	0.06	0.99
Heart_Atrial_Appendage	ENSG00000273483.1	-597059	297	147	9.02E-01	-0.01	0.07	0.99
Adipose_Subcutaneous	ENSG00000233337.1	483868	442	214	9.02E-01	-0.01	0.07	0.99
Heart_Atrial_Appendage	ENSG00000155367.11	-794095	297	147	9.04E-01	0.01	0.05	0.99

Esophagus_Mucosa	ENSG00000121933.13	357420	407	208	9.05E-01	0.00	0.04	0.99
Brain_Amygdala	ENSG00000121931.11	968421	100	47	9.05E-01	0.02	0.14	0.99
Brain_Substantia_nigra	ENSG00000143110.7	447590	88	49	9.05E-01	-0.01	0.08	0.99
Uterus	ENSG00000231437.3	-68388	111	63	9.05E-01	-0.02	0.14	0.99
Cells_Transformed_fibroblasts	ENSG00000116455.9	472519	343	175	9.07E-01	0.00	0.02	0.99
Breast_Mammary_Tissue	ENSG00000085465.11	493605	290	148	9.07E-01	-0.01	0.05	0.99
Esophagus_Mucosa	ENSG00000238975.1	-731206	407	208	9.07E-01	-0.01	0.07	0.99
Brain_Cortex	ENSG00000243960.1	482557	158	79	9.07E-01	-0.02	0.14	0.99
Thyroid	ENSG00000232811.1	977915	446	224	9.08E-01	0.01	0.06	0.99
Spleen	ENSG00000143079.10	-474799	162	55	9.08E-01	-0.01	0.08	0.99
Brain_Putamen_basal_ganglia	ENSG00000197852.8	168128	124	71	9.08E-01	0.01	0.13	0.99
Adipose_Visceral_Omentum	ENSG00000273010.1	958080	355	172	9.09E-01	-0.01	0.07	0.99
Testis	ENSG00000121931.11	968421	259	128	9.10E-01	0.00	0.03	0.99
Brain_Spinal_cord_cervical_c-1	ENSG00000155367.11	-794095	91	49	9.10E-01	0.01	0.10	0.99
Brain_Caudate_basal_ganglia	ENSG00000155363.14	-751759	160	88	9.10E-01	0.01	0.06	0.99
Testis	ENSG00000224167.1	-955558	259	128	9.10E-01	0.01	0.08	0.99
Brain_Anterior_cingulate_cortex_BA24	ENSG00000231246.1	-439146	121	65	9.11E-01	0.01	0.13	0.99
Adipose_Subcutaneous	ENSG00000273483.1	-597059	442	214	9.12E-01	0.01	0.07	0.99
Skin_Not_Sun_Exposed_Suprapubic	ENSG00000232811.1	977915	387	200	9.12E-01	-0.01	0.07	0.99
Esophagus_Gastroesophageal_Junction	ENSG00000273483.1	-597059	244	110	9.12E-01	0.01	0.07	0.99
Ovary	ENSG00000238975.1	-731206	133	71	9.12E-01	0.01	0.12	0.99
Colon_Transverse	ENSG00000260948.1	488314	274	119	9.13E-01	-0.01	0.07	0.99
Thyroid	ENSG00000233337.1	483868	446	224	9.14E-01	-0.01	0.06	0.99
Adipose_Subcutaneous	ENSG00000238975.1	-731206	442	214	9.14E-01	-0.01	0.07	0.99
Breast_Mammary_Tissue	ENSG00000197852.8	168128	290	148	9.15E-01	0.00	0.04	0.99
Esophagus_Mucosa	ENSG00000116459.6	472490	407	208	9.16E-01	0.00	0.02	0.99
Esophagus_Mucosa	ENSG00000233337.1	483868	407	208	9.16E-01	0.01	0.08	0.99
Whole_Blood	ENSG00000121933.13	357420	407	195	9.17E-01	0.00	0.04	0.99
Skin_Sun_Exposed_Lower_leg	ENSG00000085465.11	493605	473	233	9.18E-01	0.00	0.02	0.99
Skin_Not_Sun_Exposed_Suprapubic	ENSG00000064886.9	720611	387	200	9.19E-01	0.00	0.05	0.99
Brain_Hypothalamus	ENSG00000155366.12	-786052	121	61	9.19E-01	-0.01	0.05	0.99
Heart_Atrial_Appendage	ENSG00000231346.1	312659	297	147	9.21E-01	-0.01	0.06	0.99
Colon_Sigmoid	ENSG00000231437.3	-68388	233	107	9.23E-01	0.01	0.06	0.99
Minor_Salivary_Gland	ENSG00000121933.13	357420	97	49	9.23E-01	-0.01	0.09	0.99
Brain_Cerebellum	ENSG00000007341.14	-699443	173	96	9.24E-01	-0.01	0.09	0.99
Adipose_Subcutaneous	ENSG00000143110.7	447590	442	214	9.24E-01	0.00	0.02	0.99
Thyroid	ENSG00000225075.1	-775239	446	224	9.24E-01	-0.01	0.06	0.99
Liver	ENSG00000225075.1	-775239	175	88	9.25E-01	-0.01	0.12	0.99
Whole_Blood	ENSG00000238975.1	-731206	407	195	9.25E-01	0.00	0.05	0.99
Brain_Amygdala	ENSG00000143110.7	447590	100	47	9.28E-01	0.01	0.10	0.99
Pancreas	ENSG00000116489.8	-698415	248	108	9.28E-01	0.00	0.05	0.99
Testis	ENSG00000227179.2	529563	259	128	9.28E-01	0.01	0.08	0.99
Colon_Transverse	ENSG00000085465.11	493605	274	119	9.29E-01	0.00	0.05	0.99
Thyroid	ENSG00000134255.9	781156	446	224	9.29E-01	0.00	0.03	0.99
Brain_Hypothalamus	ENSG00000173947.9	575094	121	61	9.30E-01	-0.01	0.07	0.99
Spleen	ENSG00000233337.1	483868	162	55	9.31E-01	0.01	0.13	0.99
Lung	ENSG00000155363.14	-751759	427	222	9.31E-01	0.00	0.02	0.99

Artery_Coronary	ENSG00000225075.1	-775239	173	81	9.31E-01	0.01	0.12	0.99
Brain_Cortex	ENSG00000232811.1	977915	158	79	9.31E-01	-0.01	0.13	0.99
Artery_Coronary	ENSG00000273010.1	958080	173	81	9.31E-01	0.01	0.13	0.99
Testis	ENSG00000116489.8	-698415	259	128	9.32E-01	0.00	0.04	0.99
Adipose_Visceral_Omentum	ENSG00000238975.1	-731206	355	172	9.32E-01	0.01	0.07	0.99
Thyroid	ENSG00000156171.10	781166	446	224	9.33E-01	0.00	0.04	0.99
Brain_Caudate_basal_ganglia	ENSG00000231246.1	-439146	160	88	9.33E-01	-0.01	0.11	0.99
Muscle_Skeletal	ENSG00000260948.1	488314	564	276	9.33E-01	0.00	0.04	0.99
Lung	ENSG00000143110.7	447590	427	222	9.34E-01	0.00	0.03	0.99
Esophagus_Mucosa	ENSG00000171385.5	-67773	407	208	9.36E-01	0.00	0.04	0.99
Liver	ENSG00000273483.1	-597059	175	88	9.36E-01	0.01	0.08	0.99
Spleen	ENSG00000215867.4	271838	162	55	9.40E-01	-0.01	0.16	0.99
Esophagus_Mucosa	ENSG00000273010.1	958080	407	208	9.40E-01	0.01	0.09	0.99
Uterus	ENSG00000116489.8	-698415	111	63	9.40E-01	-0.01	0.09	0.99
Adipose_Subcutaneous	ENSG00000231346.1	312659	442	214	9.41E-01	0.00	0.05	0.99
Testis	ENSG00000143079.10	-474799	259	128	9.41E-01	0.00	0.04	0.99
Whole_Blood	ENSG00000235299.2	-704842	407	195	9.41E-01	0.00	0.07	0.99
Testis	ENSG00000231346.1	312659	259	128	9.41E-01	0.00	0.06	0.99
Brain_Frontal_Cortex_BA9	ENSG00000085465.11	493605	129	73	9.43E-01	0.00	0.06	0.99
Ovary	ENSG00000184599.9	-799037	133	71	9.44E-01	-0.01	0.09	0.99
Liver	ENSG00000231346.1	312659	175	88	9.44E-01	0.01	0.09	0.99
Skin_Not_Sun_Exposed_Suprapubic	ENSG00000260948.1	488314	387	200	9.44E-01	0.00	0.05	0.99
Adipose_Subcutaneous	ENSG00000064886.9	720611	442	214	9.45E-01	0.00	0.06	0.99
Brain_Anterior_cingulate_cortex_BA24	ENSG00000064886.9	720611	121	65	9.45E-01	0.01	0.10	0.99
Brain_Cerebellum	ENSG00000231346.1	312659	173	96	9.46E-01	-0.01	0.11	0.99
Esophagus_Muscularis	ENSG00000162777.12	716847	370	188	9.47E-01	0.00	0.04	0.99
Artery_Aorta	ENSG00000243960.1	482557	299	152	9.47E-01	0.01	0.09	0.99
Thyroid	ENSG00000155366.12	-786052	446	224	9.48E-01	0.00	0.03	0.99
Ovary	ENSG00000155367.11	-794095	133	71	9.48E-01	0.01	0.10	0.99
Prostate	ENSG00000227179.2	529563	152	75	9.49E-01	-0.01	0.10	0.99
Skin_Not_Sun_Exposed_Suprapubic	ENSG00000116489.8	-698415	387	200	9.51E-01	0.00	0.03	0.99
Artery_Coronary	ENSG00000064703.7	166137	173	81	9.52E-01	0.00	0.05	0.99
Brain_Nucleus_accumbens_basal_ganglia	ENSG00000162777.12	716847	147	77	9.52E-01	0.01	0.09	0.99
Whole_Blood	ENSG00000197852.8	168128	407	195	9.52E-01	0.00	0.02	0.99
Esophagus_Mucosa	ENSG00000155366.12	-786052	407	208	9.54E-01	0.00	0.04	1.00
Colon_Transverse	ENSG00000116473.10	379164	274	119	9.55E-01	0.00	0.03	1.00
Esophagus_Gastroesophageal_Junction	ENSG00000197852.8	168128	244	110	9.56E-01	0.00	0.06	1.00
Small_Intestine_Terminal_Ileum	ENSG00000231346.1	312659	137	54	9.56E-01	0.00	0.09	1.00
Brain_Frontal_Cortex_BA9	ENSG00000155363.14	-751759	129	73	9.56E-01	0.00	0.06	1.00
Artery_Tibial	ENSG00000233337.1	483868	441	219	9.57E-01	0.00	0.07	1.00
Skin_Sun_Exposed_Lower_leg	ENSG00000121931.11	968421	473	233	9.57E-01	0.00	0.04	1.00
Uterus	ENSG00000121931.11	968421	111	63	9.58E-01	-0.01	0.11	1.00
Colon_Transverse	ENSG00000121933.13	357420	274	119	9.59E-01	0.00	0.06	1.00
Skin_Sun_Exposed_Lower_leg	ENSG00000116473.10	379164	473	233	9.60E-01	0.00	0.03	1.00
Cells_EBV-transformed_lymphocytes	ENSG00000121933.13	357420	130	68	9.60E-01	-0.01	0.13	1.00
Heart_Atrial_Appendage	ENSG00000121931.11	968421	297	147	9.61E-01	0.00	0.05	1.00
Liver	ENSG00000064703.7	166137	175	88	9.62E-01	0.00	0.08	1.00

Nerve_Tibial	ENSG00000231346.1	312659	414	202	9.62E-01	0.00	0.06	1.00
Brain_Putamen_basal_ganglia	ENSG00000224167.1	-955558	124	71	9.62E-01	0.01	0.15	1.00
Brain_Caudate_basal_ganglia	ENSG00000155366.12	-786052	160	88	9.63E-01	0.00	0.04	1.00
Pancreas	ENSG00000155363.14	-751759	248	108	9.64E-01	0.00	0.04	1.00
Spleen	ENSG00000260948.1	488314	162	55	9.65E-01	-0.01	0.12	1.00
Breast_Mammary_Tissue	ENSG00000227179.2	529563	290	148	9.65E-01	0.00	0.07	1.00
Brain_Cerebellar_Hemisphere	ENSG00000116455.9	472519	136	74	9.66E-01	0.00	0.09	1.00
Colon_Sigmoid	ENSG00000064886.9	720611	233	107	9.67E-01	0.00	0.07	1.00
Adipose_Visceral_Omentum	ENSG00000064703.7	166137	355	172	9.68E-01	0.00	0.04	1.00
Testis	ENSG00000134216.14	630520	259	128	9.68E-01	0.00	0.09	1.00
Pancreas	ENSG00000116473.10	379164	248	108	9.68E-01	0.00	0.05	1.00
Artery_Tibial	ENSG00000116489.8	-698415	441	219	9.68E-01	0.00	0.04	1.00
Whole_Blood	ENSG00000116459.6	472490	407	195	9.68E-01	0.00	0.02	1.00
Stomach	ENSG00000064703.7	166137	262	107	9.71E-01	0.00	0.05	1.00
Uterus	ENSG00000085465.11	493605	111	63	9.72E-01	0.00	0.10	1.00
Uterus	ENSG00000162777.12	716847	111	63	9.72E-01	0.00	0.10	1.00
Adipose_Visceral_Omentum	ENSG00000121931.11	968421	355	172	9.73E-01	0.00	0.05	1.00
Brain_Cerebellum	ENSG00000143110.7	447590	173	96	9.73E-01	0.00	0.07	1.00
Adipose_Subcutaneous	ENSG00000203878.7	641323	442	214	9.74E-01	0.00	0.06	1.00
Adipose_Visceral_Omentum	ENSG00000184599.9	-799037	355	172	9.74E-01	0.00	0.06	1.00
Ovary	ENSG00000143079.10	-474799	133	71	9.76E-01	0.00	0.07	1.00
Esophagus_Gastroesophageal_Junction	ENSG00000134255.9	781156	244	110	9.76E-01	0.00	0.05	1.00
Brain_Cerebellum	ENSG00000134255.9	781156	173	96	9.76E-01	0.00	0.06	1.00
Small_Intestine_Terminal_Ileum	ENSG00000085465.11	493605	137	54	9.77E-01	0.00	0.07	1.00
Brain_Substantia_nigra	ENSG00000121931.11	968421	88	49	9.80E-01	0.00	0.12	1.00
Breast_Mammary_Tissue	ENSG00000173947.9	575094	290	148	9.80E-01	0.00	0.04	1.00
Nerve_Tibial	ENSG00000134245.13	-545159	414	202	9.81E-01	0.00	0.04	1.00
Muscle_Skeletal	ENSG00000121933.13	357420	564	276	9.81E-01	0.00	0.04	1.00
Brain_Caudate_basal_ganglia	ENSG00000232811.1	977915	160	88	9.81E-01	0.00	0.12	1.00
Colon_Transverse	ENSG00000232811.1	977915	274	119	9.83E-01	0.00	0.08	1.00
Brain_Anterior_cingulate_cortex_BA24	ENSG00000171385.5	-67773	121	65	9.84E-01	0.00	0.06	1.00
Adipose_Subcutaneous	ENSG00000134255.9	781156	442	214	9.85E-01	0.00	0.03	1.00
Stomach	ENSG00000162777.12	716847	262	107	9.86E-01	0.00	0.04	1.00
Brain_Anterior_cingulate_cortex_BA24	ENSG00000064703.7	166137	121	65	9.86E-01	0.00	0.10	1.00
Skin_Not_Sun_Exposed_Suprapubic	ENSG00000243960.1	482557	387	200	9.86E-01	0.00	0.07	1.00
Liver	ENSG00000260948.1	488314	175	88	9.86E-01	0.00	0.11	1.00
Esophagus_Muscularis	ENSG00000134245.13	-545159	370	188	9.87E-01	0.00	0.06	1.00
Testis	ENSG00000007341.14	-699443	259	128	9.88E-01	0.00	0.05	1.00
Brain_Cerebellar_Hemisphere	ENSG00000143110.7	447590	136	74	9.88E-01	0.00	0.06	1.00
Pituitary	ENSG00000273010.1	958080	183	95	9.88E-01	0.00	0.12	1.00
Esophagus_Gastroesophageal_Junction	ENSG00000243960.1	482557	244	110	9.89E-01	0.00	0.11	1.00
Uterus	ENSG00000173947.9	575094	111	63	9.90E-01	0.00	0.09	1.00
Minor_Salivary_Gland	ENSG00000197852.8	168128	97	49	9.90E-01	0.00	0.12	1.00
Brain_Cortex	ENSG00000116489.8	-698415	158	79	9.91E-01	0.00	0.06	1.00
Nerve_Tibial	ENSG00000233337.1	483868	414	202	9.92E-01	0.00	0.06	1.00
Artery_Tibial	ENSG00000162777.12	716847	441	219	9.92E-01	0.00	0.03	1.00
Adipose_Subcutaneous	ENSG00000155366.12	-786052	442	214	9.93E-01	0.00	0.03	1.00

Vagina	ENSG00000064703.7	166137	115	59	9.94E-01	0.00	0.08	1.00
Heart_Left_Ventricle	ENSG00000116473.10	379164	303	154	9.95E-01	0.00	0.03	1.00
Adipose_Subcutaneous	ENSG00000121933.13	357420	442	214	9.95E-01	0.00	0.03	1.00
Small_Intestine_Terminal_Ileum	ENSG00000261654.1	979029	137	54	9.95E-01	0.00	0.10	1.00
Skin_Sun_Exposed_Lower_leg	ENSG00000225075.1	-775239	473	233	9.96E-01	0.00	0.07	1.00
Brain_Nucleus_accumbens_basal_ganglia	ENSG00000260948.1	488314	147	77	9.97E-01	0.00	0.10	1.00
Skin_Sun_Exposed_Lower_leg	ENSG00000184599.9	-799037	473	233	9.97E-01	0.00	0.06	1.00
Brain_Cortex	ENSG00000156171.10	781166	158	79	9.97E-01	0.00	0.10	1.00
Prostate	ENSG00000171385.5	-67773	152	75	9.98E-01	0.00	0.09	1.00
Small_Intestine_Terminal_Ileum	ENSG00000215866.3	-929261	137	54	9.99E-01	0.00	0.15	1.00
Brain_Anterior_cingulate_cortex_BA24	ENSG00000227811.2	181541	121	65	9.99E-01	0.00	0.12	1.00
Small_Intestine_Terminal_Ileum	ENSG00000273483.1	-597059	137	54	9.99E-01	0.00	0.12	1.00

tss_distance: distance in bp to transcription start site; slope_se: standard error of the estimated regression slope; MAC: minor allele count; FDR: false discovery rate

Supplementary Table 5: Co-localization results for all tissues with significant cis eQTL associations of rs1545300

Heart Left Ventricle						
**transcript**	**Gene**	**b_SMR**	**se_SMR**	**p_SMR**	**p_HEIDI**	**nsnp_HEIDI**
ENSG00000232811.1	*RP11-96K19.2*	4.01	8.08	6.20E-01	NA	NA
ENSG00000121931.11	*LRIF1*	13.53	44.82	7.63E-01	NA	NA
ENSG00000273010.1	*RP11-96K19.5*	-2.51	2.82	3.73E-01	NA	NA
ENSG00000156171.10	*DRAM2*	2.66	2.17	2.20E-01	NA	NA
ENSG00000134255.9	*CEPT1*	2.86	2.36	2.26E-01	NA	NA
ENSG00000064886.9	*CHI3L2*	-4.24	6.68	5.26E-01	NA	NA
ENSG00000162777.12	*DENND2D*	3.76	3.93	3.39E-01	NA	NA
ENSG00000173947.9	*PIFO*	9.21	18.14	6.12E-01	NA	NA
ENSG00000085465.11	*OVGP1*	2.50	1.57	1.10E-01	NA	NA
ENSG00000260948.1	*RP11-552M11.8*	-2.21	1.99	2.65E-01	NA	NA
ENSG00000233337.1	*UBE2FP3*	8.53	37.89	8.22E-01	NA	NA
ENSG00000243960.1	*RP11-552M11.4*	8.27	35.09	8.14E-01	NA	NA
ENSG00000116455.9	*WDR77*	-50.98	390.71	8.96E-01	NA	NA
ENSG00000116459.6	*ATP5F1*	-58.83	429.76	8.91E-01	NA	NA
ENSG00000143110.7	*C1orf162*	1.33	0.52	1.10E-02	NA	NA
ENSG00000116473.10	*RAP1A*	-1019.00	151743.00	9.95E-01	NA	NA
ENSG00000121933.13	*ADORA3*	4.91	6.73	4.66E-01	NA	NA
ENSG00000231346.1	*RP5-836N10.1*	-10.55	33.21	7.51E-01	NA	NA
ENSG00000227811.2	*RP4-773A18.4*	-1.64	0.96	8.84E-02	NA	NA
ENSG00000197852.8	*FAM212B*	-31.29	212.48	8.83E-01	NA	NA
ENSG00000064703.7	*DDX20*	-8.29	16.20	6.09E-01	NA	NA
**ENSG00000171385.5**	***KCND3***	**1.69**	**0.53**	**1.37E-03**	**1.87E-01**	**19**
ENSG00000231437.3	*RP11-88H9.2*	-10.26	33.65	7.60E-01	NA	3
ENSG00000231246.1	*RP5-965F6.2*	6.37	16.71	7.03E-01	NA	NA
ENSG00000143079.10	*CTTNBP2NL*	-8.98	16.40	5.84E-01	NA	NA
ENSG00000134245.13	*WNT2B*	3.79	4.35	3.84E-01	NA	NA
ENSG00000273483.1	*RP4-671G15.2*	-7.66	18.89	6.85E-01	NA	NA
ENSG00000116489.8	*CAPZA1*	7.45	8.68	3.91E-01	NA	NA
ENSG00000007341.14	*ST7L*	-19.04	135.17	8.88E-01	NA	NA
ENSG00000155363.14	*MOV10*	10.16	17.21	5.55E-01	NA	NA
ENSG00000225075.1	*RP11-426L16.3*	-3.62	6.40	5.72E-01	NA	NA
ENSG00000155366.12	*RHOC*	6.08	5.97	3.08E-01	NA	NA
ENSG00000155367.11	*PPM1J*	-4.03	4.17	3.34E-01	NA	NA

Artery Tibial						
**transcript**	**Gene**	**b_SMR**	**se_SMR**	**p_SMR**	**p_HEIDI**	**nsnp_HEIDI**
ENSG00000261654.1	*RP11-96K19.4*	2.44	2.03	2.30E-01	NA	NA
ENSG00000232811.1	*RP11-96K19.2*	1.72	1.11	1.19E-01	NA	NA
ENSG00000121931.11	*LRIF1*	5.32	5.56	3.39E-01	NA	NA
ENSG00000156171.10	*DRAM2*	3.60	2.63	1.71E-01	NA	NA
ENSG00000134255.9	*CEPT1*	5.08	4.92	3.02E-01	NA	NA
ENSG00000064886.9	*CHI3L2*	4.08	4.51	3.65E-01	NA	NA
ENSG00000162777.12	*DENND2D*	625.65	63124.30	9.92E-01	NA	NA
ENSG00000173947.9	*PIFO*	6.96	13.02	5.93E-01	NA	NA
ENSG00000085465.11	*OVGP1*	-30.16	156.96	8.48E-01	NA	NA
ENSG00000260948.1	*RP11-552M11.8*	3.00	2.40	2.11E-01	NA	NA
ENSG00000233337.1	*UBE2FP3*	48.75	893.91	9.57E-01	NA	NA
ENSG00000243960.1	*RP11-552M11.4*	-5.26	10.68	6.22E-01	NA	NA
ENSG00000116455.9	*WDR77*	-15.72	37.74	6.77E-01	NA	NA
ENSG00000116459.6	*ATP5F1*	-63.05	493.02	8.98E-01	NA	NA
ENSG00000143110.7	*C1orf162*	12.96	28.64	6.51E-01	NA	NA
ENSG00000116473.10	*RAP1A*	7.10	6.51	2.76E-01	NA	NA
ENSG00000121933.13	*ADORA3*	32.65	232.74	8.88E-01	NA	NA
ENSG00000231346.1	*RP5-836N10.1*	-4.12	3.08	1.81E-01	NA	NA
ENSG00000227811.2	*RP4-773A18.4*	-1.90	1.40	1.74E-01	NA	NA
ENSG00000197852.8	*FAM212B*	5.15	5.05	3.08E-01	NA	NA
ENSG00000064703.7	*DDX20*	-5.71	5.05	2.58E-01	NA	NA
**ENSG00000171385.5**	***KCND3***	**1.02**	**0.26**	**1.15E-04**	**4.41E-02**	**12**
ENSG00000231437.3	*RP11-88H9.2*	7.84	16.99	6.44E-01	NA	NA
ENSG00000231246.1	*RP5-965F6.2*	9.26	28.04	7.41E-01	NA	NA
ENSG00000143079.10	*CTTNBP2NL*	5.90	6.23	3.43E-01	NA	NA
ENSG00000134245.13	*WNT2B*	-17.94	67.70	7.91E-01	NA	NA
ENSG00000273483.1	*RP4-671G15.2*	3.58	4.36	4.11E-01	NA	NA
ENSG00000116489.8	*CAPZA1*	140.56	3549.05	9.68E-01	NA	NA
ENSG00000007341.14	*ST7L*	1.67	0.87	5.50E-02	NA	NA
ENSG00000155363.14	*MOV10*	4.60	3.59	2.01E-01	NA	NA
ENSG00000155366.12	*RHOC*	6.92	5.57	2.14E-01	NA	NA
ENSG00000155367.11	*PPM1J*	-3.18	2.17	1.43E-01	NA	NA

Minor Salivary Gland **transcript**	**Gene**	**b_SMR**	**se_SMR**	**p_SMR**	**p_HEIDI**	**nsnp_HEIDI**
ENSG00000261654.1	*RP11-96K19.4*	-0.60	0.28	3.31E-02	NA	NA
ENSG00000232811.1	*RP11-96K19.2*	-0.58	0.27	3.06E-02	NA	NA
ENSG00000121931.11	*LRIF1*	-1.28	0.94	1.76E-01	NA	NA
ENSG00000273010.1	*RP11-96K19.5*	1.67	1.99	4.03E-01	NA	NA
ENSG00000156171.10	*DRAM2*	0.73	0.33	2.48E-02	2.69E-02	3
**ENSG00000134255.9**	***CEPT1***	**0.57**	**0.18**	**1.51E-03**	**1.70E-03**	**12**
ENSG00000064886.9	*CHI3L2*	1.73	1.11	1.18E-01	NA	NA
ENSG00000162777.12	*DENND2D*	1.85	1.57	2.38E-01	5.91E-02	6
ENSG00000173947.9	*PIFO*	-0.87	0.36	1.44E-02	NA	NA
ENSG00000085465.11	*OVGP1*	-1.70	1.34	2.04E-01	NA	NA
ENSG00000260948.1	*RP11-552M11.8*	-0.80	0.37	2.87E-02	NA	NA
ENSG00000233337.1	*UBE2FP3*	-1.53	1.13	1.74E-01	NA	NA
ENSG00000243960.1	*RP11-552M11.4*	-2.03	2.40	3.97E-01	NA	NA
ENSG00000116455.9	*WDR77*	6.57	18.71	7.26E-01	NA	NA
ENSG00000116459.6	*ATP5F1*	-6.38	13.12	6.27E-01	NA	NA
ENSG00000143110.7	*C1orf162*	-2.04	1.92	2.87E-01	NA	NA
ENSG00000116473.10	*RAP1A*	5.59	16.79	7.39E-01	NA	NA
ENSG00000121933.13	*ADORA3*	23.76	245.92	9.23E-01	NA	NA
ENSG00000231346.1	*RP5-836N10.1*	0.77	0.39	4.74E-02	NA	NA
ENSG00000197852.8	*FAM212B*	132.35	10650.60	9.90E-01	NA	NA
ENSG00000064703.7	*DDX20*	-3.78	8.65	6.62E-01	NA	NA
ENSG00000171385.5	*KCND3*	1.51	1.14	1.83E-01	NA	NA
ENSG00000231437.3	*RP11-88H9.2*	1.72	1.87	3.58E-01	NA	NA
ENSG00000231246.1	*RP5-965F6.2*	11.43	82.98	8.90E-01	NA	NA
ENSG00000143079.10	*CTTNBP2NL*	5.68	8.57	5.07E-01	NA	NA
ENSG00000134245.13	*WNT2B*	1.97	2.02	3.29E-01	NA	NA
ENSG00000273483.1	*RP4-671G15.2*	-0.89	0.57	1.21E-01	NA	NA
ENSG00000116489.8	*CAPZA1*	4.24	5.45	4.36E-01	NA	NA
ENSG00000007341.14	*ST7L*	0.71	0.42	9.49E-02	NA	NA
ENSG00000238975.1	*snoU13*	2.05	3.03	4.97E-01	NA	NA
ENSG00000155363.14	*MOV10*	1.97	1.70	2.46E-01	NA	NA
ENSG00000225075.1	*RP11-426L16.3*	6.87	35.70	8.47E-01	NA	NA
ENSG00000155366.12	*RHOC*	-3.20	3.09	3.01E-01	NA	NA
ENSG00000155367.11	*PPM1J*	3.40	6.75	6.14E-01	NA	NA
ENSG00000184599.9	*FAM19A3*	-1.49	1.60	3.53E-01	NA	NA

b_SMR, se_SMR, p_SMR: effect, standard error, p-value of the SMR test; p_HEIDI, nsnp_HEIDI: p-value, number of SNPs of the HEIDI test for heterogeneity. Co-localizations with p_SMR <0.01 are shown in bold.

Supplementary Table 6: Study genotyping information

	Study	BRIGHT	CHRIS	GHS1	GHS2	GRAPHIC
	**Ethnicity**	European	European	European	European	European
	**Country**	UK	Italy	Germany	Germany	United Kingdom
	**Study design**	Hypertensive cases	Population-based	Population-based	Population-based	Nuclear families (Offspring excluded)
**GENOTYPING**	**Genotyping centre**	Affymetrix, Inc., USA and Wellcome Trust Sanger Inst.(UK)	Life and Brain, Bonn (Germany)	Affymetrix, Inc., USA	Affymetrix, Inc., USA	
	**Genotyping Array**	Affymetrix GeneChip 500k array	Illumina Human OmniExpressExome	Affymetrix SNP 6.0	Affymetrix SNP 6.0	Illumina human omniexpress 12 V1
	**Genotyping calling algorithm**	CHIAMO	GenomeStudio, Z call	Birdseed2	Birdseed2	Illumina Genomestudio
**SAMPLE GENOTYPING QC**	**Sample call rate**	≥0.97	>99%	>95%	>95%	
	**Other exclusions**		gender mismatch, excess of heterozygosity, excess of mendelian errors	gender mismatch, excess of IBS (> 95%), excess of heterozygosity (hetFDR > 0.01)	gender mismatch, excess of IBS (> 95%), excess of heterozygosity (hetFDR > 0.01)	
**SNP QC**	**MAF (required)**	0%	NA	NA	NA	>1%
	**HWE (required)**	≥1E-7	P>10^-6	10^-4	10^-4	p>10-6
	**Call rate**	≥95%	>99%	>98%	>98%	<95% (or<99% if SNP has MAF<5%)
	**Exclude duplicates**	NA	NA	NA	NA	NA
	**Other**	NA	NA	NA	NA	NA
	**SNPs for imputation**	446,472	610,883	662,405	673,914	612,432
**Imputation**	**Reference Panel**	1000G March 2012	1000G Ph1 v3	1000G Ph1 v2	1000G Ph1 v2	1000Gv3
	**Build**	37	37	37	37	37
	**Software for imputation**	Minimac	minimac 2	MACH, minimac	MACH, minimac	IMPUTE v2
	**Filters**	Imputation quality >0.1	Rsq < 0.3	none	none	MAF>0
	**SNPs for analysis**		14,898,673	15,725,774	15,616,273	14,551,978
**Data Analysis**	**Adjustments**	sex, age	sex, age	sex, age	sex, age	sex, age
	**Software for analysis**	mach2qtl	SAIGE	SNPtest	SNPtest	SNPTEST
	**Lambda GC**	1.01	1.01	0.93	0.85	0.81
**REFERENCES**	**Reference study description (PMID)**	12826435	26541195, 28374192			18443236
	**Study link/ website**	NA	http://www.eurac.edu/en/research/health/biomed/projects/Pages/default.aspx	NA	NA	www2.le.ac.uk/projects/bru/our-research/research-themes/genetics-and-biomarkers/graphic-2

	Study	Lifelines	Rotterdam Study I	Rotterdam Study II	Rotterdam Study III
	Ethnicity	European	European	European	European
	Country	The Netherlands	The Netherlands	The Netherlands	The Netherlands
	Study design	Population-based	General Population-Based	General Population-Based	General Population-Based
GENOTYPING	Genotyping centre	UMCG	Erasmus University Medical Center (Rotterdam, the Netherlands)	Erasmus University Medical Center (Rotterdam, the Netherlands)	Erasmus University Medical Center (Rotterdam, the Netherlands)
	Genotyping Array	Illumina Cyto SNP12 v2	Illumina 550 duo	Illumina 550 duo	Illumina 610 quad
	Genotyping calling algorithm	Illumina Genomestudio	Beadstudio Genecall	Beadstudio Genecall	Beadstudio Genecall
SAMPLE GENOTYPING QC	Sample call rate	>95%	≥97.5%	≥97.5%	≥97.5%
	Other exclusions	close relatives (pi-hat>0.4), non-caucasian, sex mismatch	1) Missing DNA<br>2) Gender mismatch with typed X-linked markers<br>3) Excess autosomal heterozygosity > 0.336~FDR>0.1%<br>4) Duplicates and/or 1st or 2nd degree relatives using IBS probabilities >97% from PLINK<br>5) Ethnic outliers using IBS distances > 3SD		
SNP QC	MAF (required)	>1%	>1%	>1%	>1%
	HWE (required)	10-4	P>10-6	P>10-6	P>10-6
	CALL RATE (required)	>95%	≥97.5%	≥97.5%	≥97.5%
	Exclude duplicates	NA	NA	NA	NA
	Other	NA	NA	NA	NA
	SNPs for imputation	257,581	512,849	466,389	514,073
Imputation	Reference Panel	1000G v3	1000Gv3	1000Gv3	1000Gv3
	Build	37	37	37	37
	Software for imputation	BEAGLE v.3.1.0	MACH	MACH	MACH
	Filters	MAF>0	none	none	none
	SNPs for analysis	28.681.763	29,079,054	29,079,054	29,079,054
Data Analysis	Adjustments	sex, age, PC1-6	sex, age	sex, age	sex, age
	Software for analysis	PLINK	ProbABEL	ProbABEL	ProbABEL
	Lambda GC	0.99	0.88	0.92	0.89
REFERENCES	Reference study description (PMID)	18075776, 25502107	1833235, 29064009	1833235, 29064009	1833235, 29064009
	Study link/ website	www.lifelines.nl	http://www.epib.nl/research/ergo.htm	http://www.epib.nl/research/ergo.htm	http://www.epib.nl/research/ergo.htm

	Study	SHIP	SHIP-Trend	TwinsUK
	Ethnicity	European	European	European
	Country	Germany	Germany	United Kingdom
	Study design	Population-based	Population-based	Twins
GENOTYPING	Genotyping centre	Affymetrix, Inc., USA	Helmholtz Zentrum München, Germany	Wellcome Trust Sanger Inst.(UK), CIDR (USA), Centre National de Genotypage (France), Duke University (USA), Helsinki University (Finland)
	Genotyping Array	Affymetrix SNP 6.0	Illumina Omni 2.5	Illumina TruSeq, Illumina Hap300, Hap550, Hap610
	Genotyping calling algorithm	Birdseed2	GenCall v1.0	Illuminus
SAMPLE GENOTYPING QC	Sample call rate	>92%	≥94%	≥95%
	Other exclusions	duplicate samples (by IBS) or reported/genotyped gender mismatch	duplicate samples (by IBS) or reported/genotyped gender mismatch	gender mismatch with X chromosomes genotypes
SNP QC	MAF (required)	NA	>0%	>0 %
	HWE (required)	P>0.0001	P>0.0001	P>10-6
	CALL RATE (required)	>80%	>90%	>95%
	Exclude duplicates	chr:position:type	chr:position:type	NA
	Other	position mapping problem from b36 to b37	position mapping problem from b36 to b37	NA
	SNPs for imputation	905,910	1,824,743	>303,940
Imputation	Reference Panel	1000Gv3	1000Gv3	1000Gv3
	Build	37	37	37
	Software for imputation	IMPUTE v2.2.2	IMPUTE v2.2.2	IMPUTE2
	Filters	eur.maf=0	eur.maf=0	Info score >0.4 (imputed)
	SNPs for analysis	17,533,349	17,585,496	37,426,733
Data Analysis	Adjustments	sex, age	sex, age	sex, age, assay
	Software for analysis	QUICKTEST v0.95	QUICKTEST v0.95	SNPTEST2
	Lambda GC	0.90	0.86	1.03
REFERENCES	Reference study description (PMID)	20167617	20167617	17254428
	Study link/ website	http://ship.community-medicine.de/	http://ship.community-medicine.de/	http://www.twinsuk.ac.uk

Supplementary Figures

Supplementary Figure 1: Manhattan plot of the combined GWAS meta-analysis

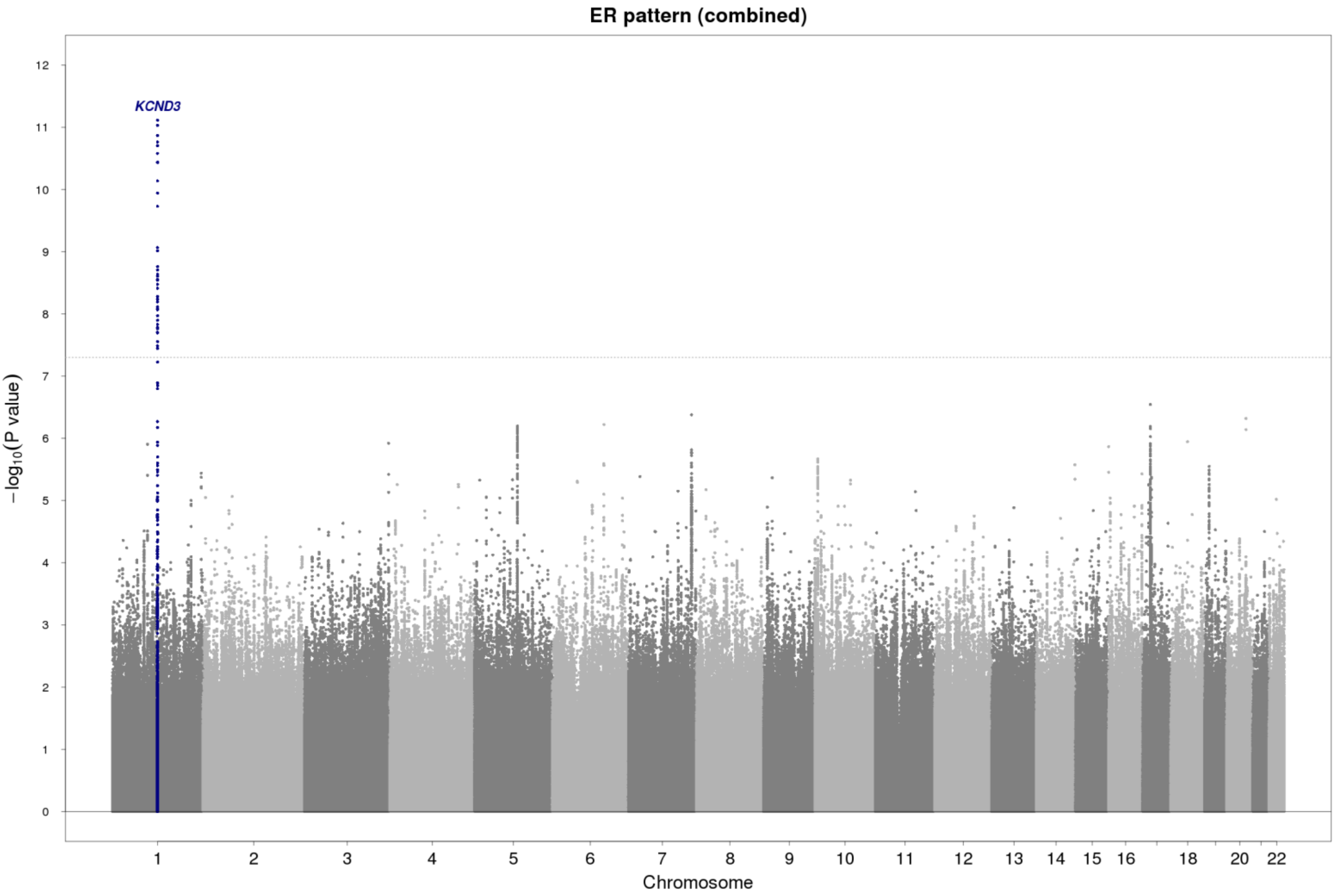

SNPs are plotted on the x-axis according to their position on each chromosome with the -$\log_{10}$(p-value) of the association test on the y-axis. The solid horizontal line indicates the threshold for genome-wide significance, $5x10^{-8}$. The labels show the closest gene.

Supplementary Figure 2: QQ plot of the combined GWAS meta-analysis

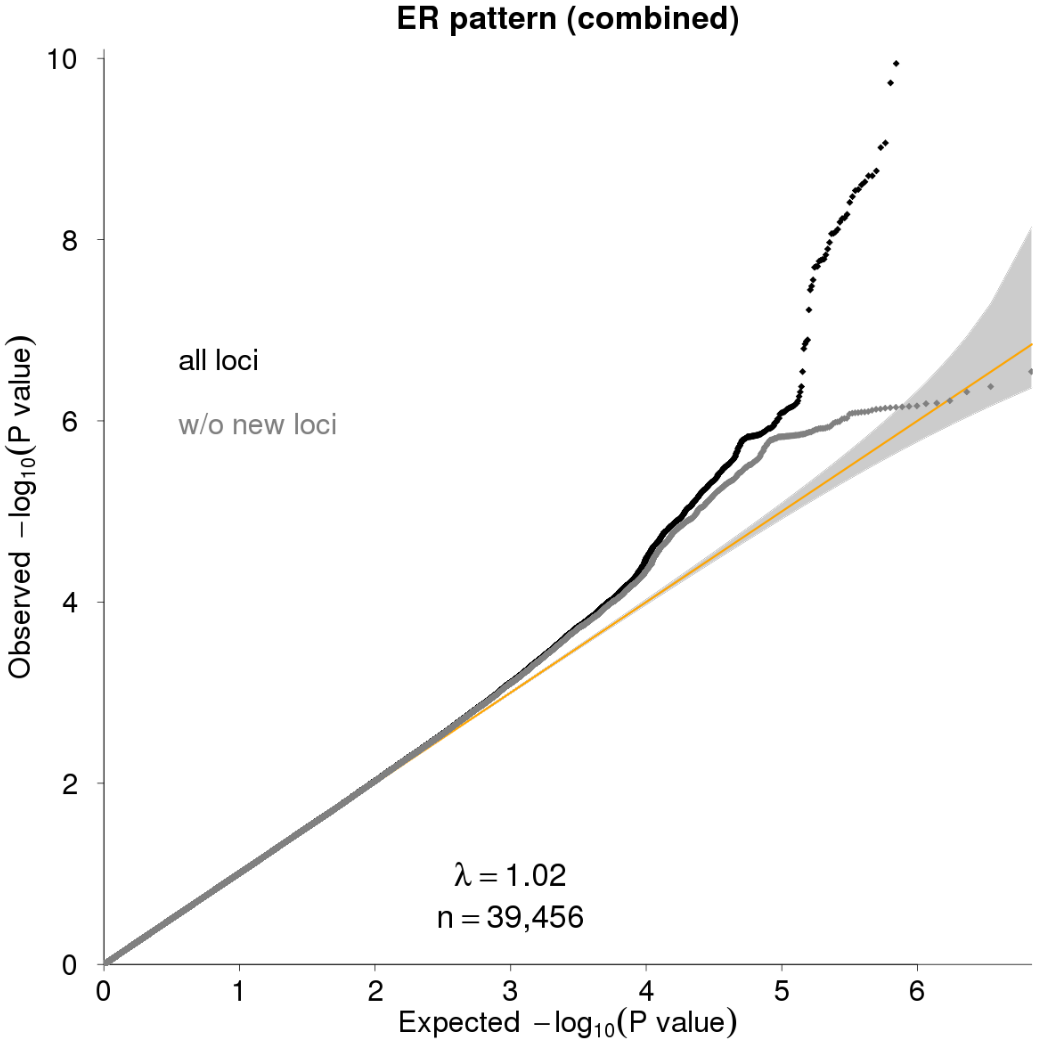

The observed p-values of the association test are plotted on the y-axis against their expected distribution under the null hypothesis of no association on the x-axis. Results for all SNPs are shown in black, and results after removal of loci (±500kb of the lead SNP) genome-wide significantly ($p<5x10^{-8}$) associated with the trait are shown in gray. Gray bands represent 95% confidence intervals. λ: lambda, genomic control parameter; n: sample size.

Supplementary Figure 3: Regional association plot of rs17029069

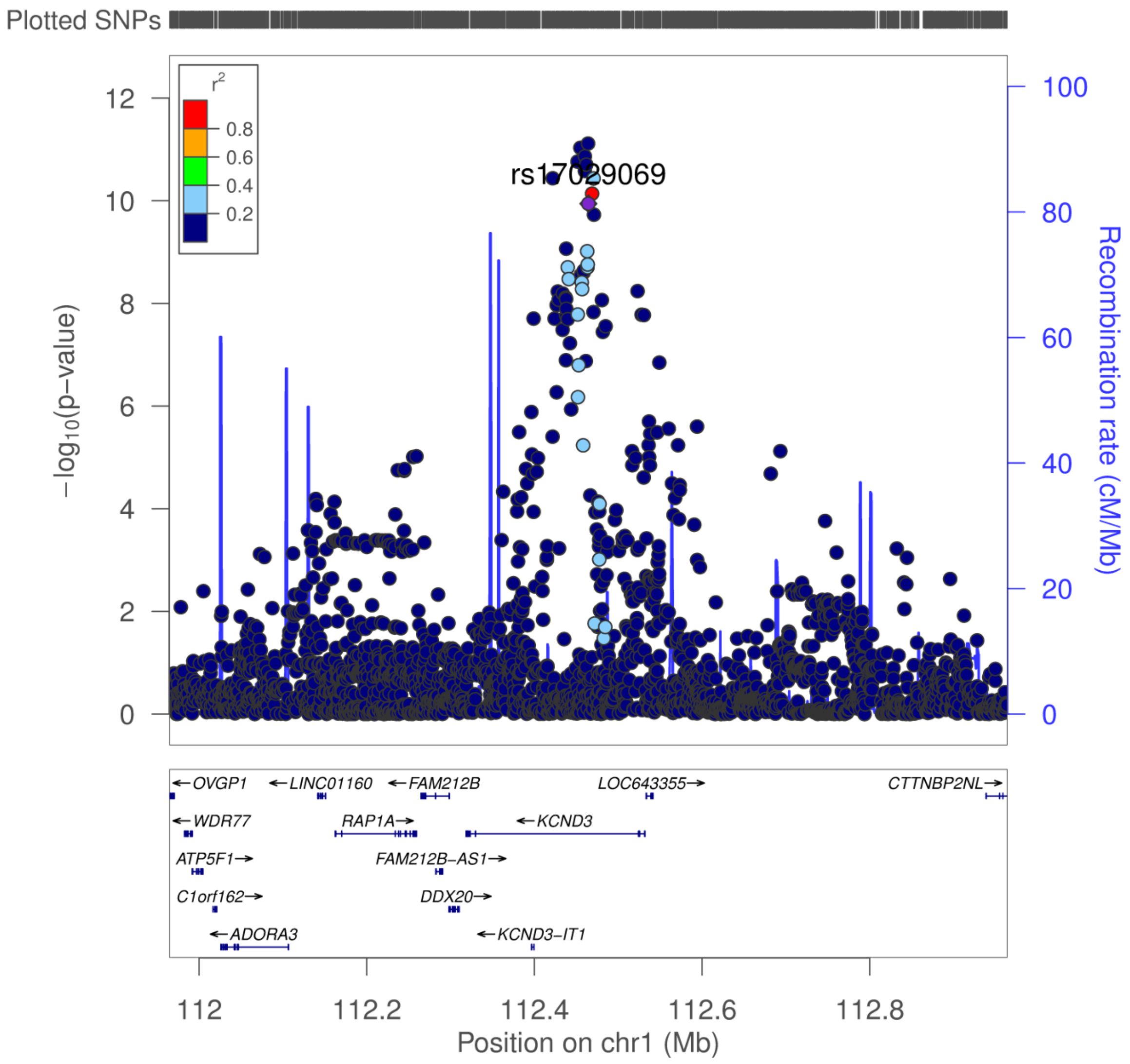

Regional association plots are shown for a reported candidate SNP rs17029069 of a previous GWAS. Correlation with the SNP (purple) is estimated based on the 1000 Genomes EUR reference samples. Plots were generated using the website of LocusZoom (Pruim, R. J. *et al.* Bioinformatics, 2010). Genetic positions refer to GRCh37/hg19 coordinates.
